# Molecular signatures of local adaptation to light in Norway spruce

**DOI:** 10.1007/s00425-020-03517-9

**Published:** 2021-01-28

**Authors:** Sonali Sachin Ranade, María Rosario García-Gil

**Affiliations:** 1 Department of Forest Genetics and Plant Physiology, Umeå Plant Science Centre, Swedish University of Agricultural Sciences, 901 83 Umeå, Sweden; 2 Department of Plant Physiology, Umeå Plant Science Centre, University of Umeå, 901 87 Umeå, Sweden

**Keywords:** Conifers, Exome capture, Immunity, Latitudinal cline, Lignin, Local adaptation, Missense variation, Norway spruce, R:FR ratio, Response to shade, SNP

## Abstract

**Main conclusion:**

Transcriptomic and exome capture analysis reveal an adaptive cline for shade tolerance in Norway spruce. Genes involved in the lignin pathway and immunity seem to play a potential role in contributing towards local adaptation to light.

**Abstract:**

The study of natural variation is an efficient method to elucidate how plants adapt to local climatic conditions, a key process for the evolution of a species. Norway spruce is a shade-tolerant conifer in which the requirement of far-red light for growth increases latitudinally northwards. The objective of the study is to characterize the genetic control of local adaptation to light enriched in far-red in Norway spruce, motivated by a latitudinal gradient for the Red:Far-red (R:FR) ratio to which Norway spruce has been proven to be genetically adapted. We have established the genomic signatures of local adaptation by conducting transcriptomic (total RNA-sequencing) and genomic analyses (exome capture), for the identification of genes differentially regulated along the cline. RNA-sequencing revealed 274 differentially expressed genes in response to SHADE (low R:FR light), between the southern and northern natural populations in Sweden. Exome capture included analysis of a uniquely large data set (1654 trees) that revealed missense variations in coding regions of nine differentially expressed candidate genes, which followed a latitudinal cline in allele and genotype frequencies. These genes included five transcription factors involved in vital processes like bud-set/bud-flush, lignin pathway, and cold acclimation and other genes that take part in cell-wall remodeling, secondary cell-wall thickening, response to starvation, and immunity. Based on these results, we suggest that the northern populations might not only be able to adjust their growing season in response to low R:FR light, but they may also be better adapted towards disease resistance by up-regulation of the lignin pathway that is linked to immunity. This forms a concrete basis for local adaptation to light quality in Norway spruce, one of the most economically important conifer tree species in Sweden.

**Supplementary Information:**

The online version contains supplementary material available at 10.1007/s00425-020-03517-9.

## Introduction

Research indicates that local adaptation in conifer populations will be affected by major disturbances due to an increase in average temperature, change in precipitation regimes and rise in new biotic stresses, which could compromise forest survival and sustainability at their present locations (Leinonen 1996; Millar and Stephenson 2015). Given the prognosis of a rapid climatic change (IPCC 2019), forest breeders and conservationists are urging for a better understanding of the genetic and genomic basis underlying local adaptation. This aspect is crucial to design effective spatial migration actions to transfer forest trees into their optimal ecological niches or to assist the trees to adapt to the new conditions at their current location (Aitken et al. 2008). Understanding the basis of local adaptation at the molecular level has been at the forefront of genetics research aiming for clues on genomic regions of functional relevance (Hoban et al. 2016).

In the current study, we aimed to identify the genomic signals of local adaptation in response to low Red:Far-red (R:FR) ratio (SHADE) in Norway spruce which is shade tolerant. R:FR ratio is considered a reliable indicator of the degree of shade and low values of it can activate shade-avoidance and shade-tolerance responses (Ranade et al. 2019). There is an increase in requirement of FR (lower R:FR) with latitude from the south towards the north in Norway spruce, in order to maintain growth (Clapham et al. 1998; Mølmann et al. 2006). In other words, in response to light deficiency in FR, trees of northern origin stopped growing and entered dormancy whereas southern trees continued to grow. A similar latitudinal response has been described in *Salix pentandra* L. and Scots pine (*Pinus sylvestris* L.) (Juntilla and Kaurin 1985; Clapham et al. 2002; Mølmann et al. 2006). This latitudinal variation has been suggested to be an adaptive response to the prolonged end-of-day (EOD) FR-enriched light condition (twilight) (Juntilla and Kaurin 1985), which characterizes the northern latitudes during the summer solstice (Nilsen 1985). Thus, trees in the north may have adopted a light system that utilizes FR as a primary cue for growth regulation, while at the southern latitudes duration of darkness may be a more critical factor (Juntilla and Kaurin 1985; Clapham et al. 1998). Thus, northern trees have optimized growth under low R:FR ratios during the time of the day called twilight. EOD FR-enriched tolerance and shade tolerance are both triggered by low R:FR ratio and by the existence of common molecular components regulating both mechanisms (Johnson et al. 1994; Muller-Moule et al. 2016).

Twilight is a consequence of the 24 h-cycle during which light changes as a function of the Earth’s rotation in both intensity and spectral composition, is characterized by a daily reduction in R:FR at dawn and dusk due to an increase in FR light following atmospheric refraction (Smith 1981). The length of the twilight periods and the extent of R:FR reduction increases towards the northern latitudes. As a proxy to twilight exposure, in the current work, seedlings were treated with two contrasting values of bi-chromatic R:FR ratios representing day-light (SUN, R:FR = 1.2) or SHADE (R:FR = 0.2, as a proxy of twilight). Thus, we first studied the effect of SHADE on the morphological trait (hypocotyl length) in seedlings of Norway spruce sampled from natural populations across four latitudes in Sweden. RNA-sequencing (RNA-Seq) analysis was conducted in seedlings originating from two populations farthest apart (south, 56° 2′ N and north, 67° 2′ N) as the phenotypic (hypocotyl length) difference in response to SHADE was the highest at these two latitudes, leading to the identification of Differentially Expressed Genes (DEGs) under SHADE. In the second step with the aid of exome capture, we estimated changes in the allele/genotype frequency with reference to the DEGs in six populations latitudinally distributed across Sweden (1654 trees).

This study aims to answer the following specific questions: (i) Do we observe a phenotypic cline in the Norway spruce seedlings in response to low R:FR or SHADE?—this will be answered by comparing the hypocotyl elongation under SHADE and SUN conditions across the latitudes. (ii) Knowing that Norway spruce seems to be adapted to the local light quality conditions (Clapham et al. 1998), what are the genomic signs of local adaptation for the exposure to SHADE?—this will be answered by RNA-Seq and exome capture methodologies.

## Materials and methods

In this study, morphological trait study (germination of seed and measurement of the length of hypocotyl) in response to shade was carried out at four latitudes across Sweden and RNA-Seq was performed with the seedlings from two farthest latitudes to detect the differentially expressed genes in response to shade following the latitudinal cline. Next, 1654 trees (unrelated parents) originating from several different latitudes across Sweden, were analysed for the presence of the SNP variation in the differentially expressed genes by exome capture.

### Seed germination under SUN/SHADE and hypocotyl length measurements

Norway spruce seeds were collected from 70 unrelated trees from natural populations from four different latitudes across Sweden—Pellonhuhta (67° 2′ N, 22° 1′ E), Hallen (64° 3′ N, 14° 6′ E), Sör Amsberg (60° 3′ N, 15° 2′ E), and the southernmost population between latitude 56° and 58° (designated as 56°). These populations were selected based on previous studies in Norway spruce (Clapham et al. 1998) in Sweden that reported an adaptive latitudinal cline in response to light quality. To ensure low consanguinity and to capture a representation of the population diversity, trees were sampled at a distance of minimum 50 m from each other. Cones were dried with warm air to force release the seeds. Sound seeds were separated from the empty seeds by flotation. The percentage of germination was obtained by germinating soaked seeds on paper discs on a warm bench with controlled humidity and temperature. The percentage of germination was 98% in a batch of 200 seeds (five seeds per tree).

Stratified seeds (soaked in water at 4 °C overnight) were sown on water-saturated sterile vermiculite in growth boxes (Saveen Werner) and maintained at a constant temperature of 22 °C in Percival (LED-30 Elite series) growth cabinets. 70 sound seeds per treatment and latitude (one seed per sampled tree) were germinated under two continuous light treatments: Treatment A represented shade-like (SHADE) conditions having a R:FR ratio of 0.2 and total light intensity of 36 µmol m^−2^ s^−1^ (R, 6 µmol m^−2^ s^−1^: FR, 30 µmol m^−2^ s^−1^), and treatment B (control) represented sun-like (SUN) conditions having R:FR ratio equal to 1.2 and a total light intensity of 65 µmol m^−2^ s^−1^ (R, 35 µmol m^−2^ s^−1^: FR, 30 µmol m^−2^ s^−1^). We considered only the R and FR light qualities in this experiment, as these are the two main responsible elements that plants use to determine shade conditions and respond accordingly. Plants sense the shade as a decrease in R (or decrease in R:FR); R is primarily absorbed by the pigments and FR is reflected thereby decreasing the R:FR ratio. To mimic the natural conditions, FR intensity was kept constant under SHADE and SUN conditions and R intensity was lowered under SHADE. This experimental design was able to trigger the shade avoidance and the shade tolerance response in Scots pine and Norway spruce respectively, in our earlier work (Ranade et al. 2019).

Hypocotyl was used as a model system to study the cline under the SHADE conditions in Norway spruce. Length of hypocotyl was scored at the mm precision for each seedling at the seedling developmental stage where the hypocotyl was fully developed (Ranade et al. 2013), thus seedlings were harvested at the same developmental stage (Supplementary Fig. S1). The conifers have multiple cotyledons (5–8) and these are held together at the tip by the seed, the hypocotyl is considered to be fully developed when the seed drops off and cotyledons are set free (Supplementary Fig. S1). The number of days from sowing of the seeds to fully developed hypocotyl was approximately 17 ± 2 days, under both the light treatments. Seedling morphology data under SHADE was converted into the percentage of change with respect to SUN (100%). Thus the hypocotyl length is represented as the percentage of change in length in response to SHADE with respect to SUN and then the hypocotyl growth in the seedlings from the four latitudes included in the study is compared under SHADE, where SUN treatment is the control. Analysis of variance (ANOVA) was applied to estimate the significance of the SHADE treatment effect. All statistical analyses were conducted using R software, version 3.5.2 (R Development Core Team 2015).

### RNA-sequencing (RNA-Seq) and differential expression analysis

RNA-sequencing was performed with the samples from latitude 67° 2′ N and 56° 2′ N. Three biological replicates were prepared for each of the light treatments: A (SHADE) and B (SUN), for RNA extraction. The biological replicates were prepared by pooling three seedlings per sample to reduce variation between replicates and to increase the statistical power of the analysis (i.e. increased sensitivity to detect genes that were differentially expressed between conditions). Whole seedling was used for the extraction of RNA. Isolation of total RNA, RNA-Seq and pre-processing of RNA-Seq data was carried as described in our previous work (Ranade et al. 2019). In short, total RNA was isolated using Spectrum Plant Total RNA Kit (Sigma) following the manufacturer’s instructions. The mRNA concentration and quality were determined using NanoDrop-2000 spectrophotometer (Thermo Fisher Scientific Inc.) and Bioanalyzer 2100 (Agilent Technologies Inc.), respectively. RNA library preparation and subsequent sequencing (HiSeq 2500, Illumina) were performed at SciLifeLab (Stockholm, Sweden). The data pre-processing was performed as described here: http://www.epigenesys.eu/en/protocols/bio-informatics/1283-guidelines-for-rna-seq-data-analysis. Reads were aligned to v1.0 of the *P. abies* genome (retrieved from the PopGenIE resource; Sundell et al. 2015). The RNA-Seq data was deposited to the ENA and is accessible under the accession number PRJEB19683 (https://www.ebi.ac.uk/ena/data/view/PRJEB19683).

Statistical analysis of single-gene differential expression between the two latitudes in response to SHADE was determined using SUN as control. Analysis was performed using the Bioconductor (v3.3; Gentleman et al. 2004) DESeq2 package (v1.12.0; Love et al. 2014). FDR adjusted *P *values were used to assess significance; a common threshold of 1% was used throughout. For the data quality assessment (QA) and visualisation, the read counts were normalised using a variance stabilizing transformation as implemented in DESeq2. The metabolic pathway maps were constructed with the DEGs using MapMan 3.5.1R2. The GO categories of the DEGs involved in defence response (GO:0006952, GO:0010204, GO:0042742, GO:0050832, GO:0009817, GO:0009870, GO:0002229, GO:0002764, GO:0009627) was determined using Congenie (http://congenie.org/) and statistical analysis was performed for the number of defense-related genes expressed under SHADE in the two latitudes. Like-wise, GO categories considered for analysis of transcription factors were GO:0000981 and GO:0003700.

### Estimation of nucleotide variation (SNP) in DEGs following the latitudinal cline

A total of 1654 individuals (unrelated parents) originating from different latitudes across Sweden, were included in this study for analysis of the SNP variation following a latitudinal cline. The 1654 individuals belong to progeny trials established from the half-sib families from wild trees growing at the same latitudes as the plantations, which implies that these plantations are not domesticated trees and therefore they reflect the natural variation. Only one tree per progeny was sampled and the mothers of each progeny were unrelated. For the current analysis, trees were divided into six populations, S1-S6. S1 comprised 245 trees from latitudes 55–57, S2—213 trees from latitude 58, S3—187 trees from latitudes 59–60, S4—213 trees from latitudes 61–62, S5—573 trees from latitudes 63–64 and S6—223 trees from latitudes 65–67. This categorisation of the population was done taking into consideration the fact that the quality of light differs latitude-wise from south to north in Sweden; during the growth season (summer), the northern latitudes receive more FR as compared to the southern ones.

Details regarding the DNA extraction, exome capture, genotyping, and SNP annotation have been previously described in Baison et al. (2019). In short, total genomic DNA was extracted from 1654 trees, from dormant buds, using the Qiagen Plant DNA extraction (Qiagen, Hilden, Germany) following the manufacturer's protocol and DNA quantification was performed using the Qubit ds DNA Broad Range (BR) Assay Kit (Eugene, OR, USA). DNA library preparation and exome capture sequencing were performed at the RAPiD Genomics (Gainesville, FL, USA). Sequence capture was performed using the 40, 018 diploid probes designed and evaluated for *P. abies* (Vidalis et al. 2018). Sequencing was performed using an Illumina HiSeq 2500 instrument (San Diego, CA, USA) on the 2 × 100 bp sequencing mode. Raw reads were mapped against the *P. abies* reference genome v.1.0 and variant calling was performed using GATK HAPLOTYPECALLER v.3.6 (Van der Auwera et al. 2013). The resultant SNPs were annotated using default parameters for SNPEFF 4 (Cingolani et al. 2012).

The vcf file was filtered using settings: —min-alleles 2 —max-alleles 2 —maf 0.01 —remove-indels —minQ 10 —max-missing 0.9. Allele frequencies, genotype frequencies and Hardy–Weinberg equilibrium (HWE) *P*-values were determined using SNPassoc statistical package (Gonzalez et al. 2007). ANOVA and Tukey’s post-hoc tests (Bonferroni *P *values) were applied to determine the statistical significance in the difference in the allele and genotype frequencies across the populations included in the study. Genetic diversity among the six different populations (pairwise *F*_*ST*_ estimates) was estimated using DnaSP 6 (Rozas et al. 2017) that included both the synonymous + missense SNPs of the 54 DEGs, and synonymous + missense SNPs of the ten non-DEGs that were randomly chosen. Allele frequencies in each population with reference to the nine candidate genes (DEGs) and ten control genes (non-DEGs) were calculated and then regressed on population latitude. *R*^2^ of the linear regression was computed as the proportion of total variance of latitude explained by the frequency of each marker (Berry and Kreitman 1993). *R*^2^ is the goodness-of-fit of the linear regression model.

## Results and discussion

A low R:FR ratio is known to trigger both EOD-FR response and response to vegetative shade. In addition, these FR-related processes have been shown to share common molecular components (Johnson et al. 1994; Muller-Moule et al. 2016). These two facts suggest the speculation about a latitudinal cline for the level of shade tolerance (equivalent to that for EOD-FR). Our study has revealed a latitudinal cline for hypocotyl elongation in response to shade in Norway spruce, which indicates that previously reported clines for growth regulation by FR-enriched light (Clapham et al. 1998) directly translates into a cline for shade tolerance. In addition, we report genomic signatures in candidate genes differentially regulated under the shade that also follow a cline, which may be involved in the local adaptation to light quality.

### Effect of SHADE on seedling morphology

**Fig. 1:**
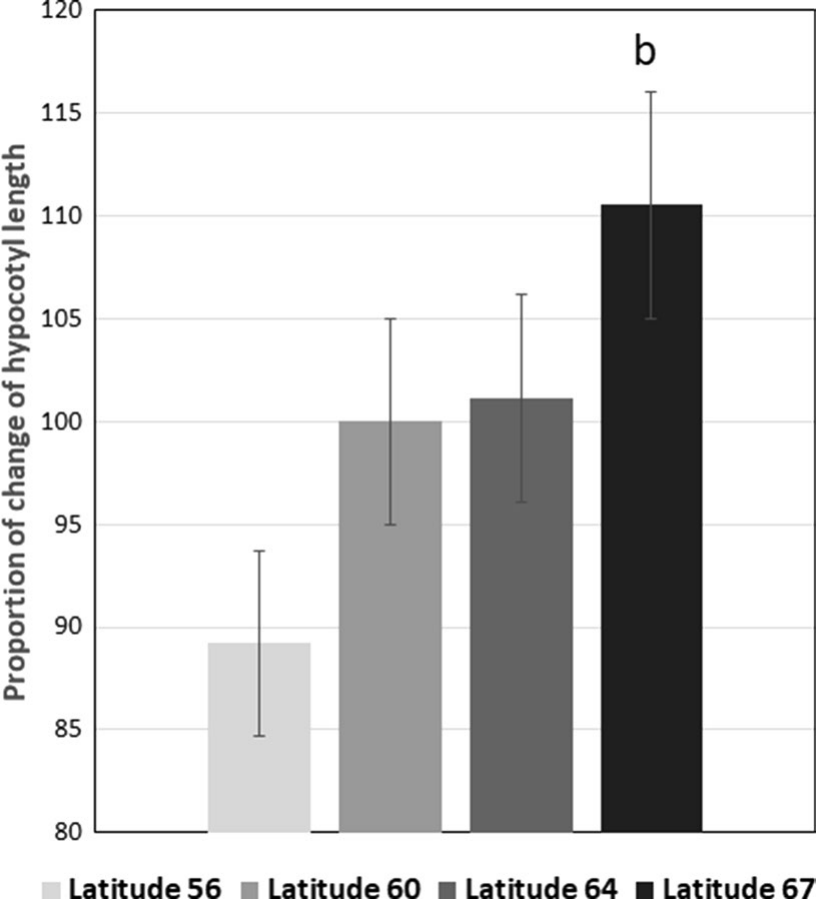
Bar plot with mean and ± SE of the proportion of change of hypocotyl length in response to SHADE as compared to SUN condition across four latitudes in Sweden that shows a clinal variation (*P* value < 0.01, Tukey’s *post-hoc* categorization is indicated above the bars). Hypocotyl length is represented as a percentage of change in length in response to SHADE with respect to SUN

There was a significant increase in the length of the hypocotyl (*P* value < 0.01, Fig. 1) towards the northern latitudes in response to SHADE. Norway spruce is considered to be shade tolerant based on an absence of effect of shade on hypocotyl elongation. The hypocotyl shows similar patterns of elongation under sun and shade (Ranade et al. 2019). In the current study, we report a cline for hypocotyl elongation in response to SHADE in Norway spruce where the hypocotyl is found to be significantly elongated under SHADE from the southern latitude to the northern ones. This indicates that the level of shade tolerance in Norway spruce may be a property of the population and shaped by the light local conditions, which is in agreement with previous studies (Ranade et al. 2019).

### Differentially expressed genes (DEGs) and latitudinal cline in SNPs

RNA-Seq analysis showed differential gene expression in response to SHADE in seedlings from the southern (56° 2′ N) and northern populations (67° 2′ N) of Norway spruce. We compared the expression data between the two latitudes in response to SHADE using SUN as the control. 141 genes were found for which the levels of expression were significantly higher in the 67° N Norway spruce population as compared to the 56°N population in response to SHADE (SHADE-67 > SHADE-56) (Supplementary Table S1). Likewise, 133 genes were detected for which the levels of expression were significantly higher in the 56°N Norway spruce population as compared to the 67°N population in response to SHADE (SHADE-67 < SHADE-56) (Supplementary Table S2).

Gene ontology (GO) analysis revealed that there were significantly (*P* value = 0.03) higher number of defense-related genes (16 genes) that were up-regulated in the northern population under SHADE as compared to the southern population (6 genes). The metabolic pathway maps (Supplementary Fig. S2–S6) that were constructed with reference to the DEGs in the two populations showed that a higher number of genes related to cell wall metabolism were up-regulated at latitude 67° 2´N as compared to latitude 56° 2′ N (Supplementary Fig. S2). Likewise, a higher number of genes involved in the phenylpropanoid pathway were up-regulated at latitude 67° 2′N (Supplementary Fig. S2–S4). Differential expression of an equal number of transcription factors (TF) was detected in both latitudes (*P* value > 0.05; 10 and eight TFs were up-regulated in the north and south, respectively, Supplementary Fig. S5).

A light intensity/shade responsive genes were detected to be up-regulated under SHADE in the northern population—*PHYTOCHROME RAPIDLY REGULATED 2* (*PAR2*) that encodes a protein which is up-regulated under the shade and acts as a negative regulator of shade avoidance response (Roig-Villanova et al. 2006). *DRACULA2 (DRA2)*, a gene with a role in regulating shade responses, thereby involved in hypocotyl elongation and control of shade induced gene expression (Gallemi et al. 2016), was detected to be up-regulated under SHADE in the northern population. One light-regulated gene was detected to be up-regulated in the southern population—*TEOSINTE BRANCHED 1*, *CYCLOIDEA AND PCF TRANSCRIPTION FACTOR 2* (*TCP2*); *TCP2* is a positive regulator of photomorphogenesis and is involved in response to blue light (He et al. 2016). There might be many other light-regulated genes in spruce that respond to SHADE, but these genes were not be detected in this study as only those genes that were differentially expressed in response to SHADE in the north and south latitudes were fetched by the current analysis. Cell wall-related genes—*CELLULOSE SYNTHASE A4* (*CESA4*) involved in secondary cell wall biosynthesis (Endler and Persson 2011) and *CINNAMOYL COA REDUCTASE* (*CCR2*) involved in lignin biosynthesis (Wadenback et al. 2008) were found to be up-regulated in the northern trees. Likewise, an immunity-specific gene was detected to be up-regulated at the northern latitude—*RESISTANT TO P. SYRINGAE 2* (*RPS2*) (Kunkel et al. 1993).

We considered bi-allelic SNPs for the study. A latitudinal cline in SNP represents an increase in the frequency of either allele along with a decrease in the frequency of the other with latitude, which is also reflected in the genotype frequencies of the particular SNP, meaning that there is increase/decrease of either of the homozygotes and the corresponding change in the frequency of heterozygotes with latitude. RNA-Seq analysis revealed 274 genes that were differentially regulated in response to SHADE between the two populations from two latitudes (56° 2´N and 67° 2´ N). Exome sequencing confirmed the presence of SNPs in 58 DEGs, out which 54 DEGs were detected with at least one missense SNP. 388 SNPs were detected in the analysis; 222 were missense and 166 were synonymous SNPs. Missense variations in coding regions of nine differentially expressed candidate genes followed a latitudinal cline in allele and genotype frequencies. A total of 62 SNPs were detected in the nine candidate DEGs; 44 were missense mutations and 18 were synonymous; 20 missense SNPs showed significant cline and eight synonymous mutations showed mild cline. Ten randomly chosen genes that were not differentially regulated under SHADE in the two Norway spruce populations were included as control genes in the study. With reference to the ten control genes, 30 SNPs were detected, 19 of which were missense mutations and 11 were synonymous; none of the SNPs detected in the control genes showed cline. We focused only on the missense mutations as those would have an effect on the protein conformation, which may contribute to the differential response to SHADE (or low R:FR), therefore we included the 54 DEGs with at least one missense SNP in the analysis. The vcf file of 54 DEGs and the vcf file of ten non-DEGs (control genes) that contains data of the exome sequencing results from 1654 trees included in the study is deposited in Zenodo, which is the open-access repository developed under the European OpenAIRE program and operated by CERN (http://doi.org/10.5281/zenodo.4038115) (Ranade and García-Gil 2020). In this article, we have discussed the nine relevant differentially expressed genes (Table 1) that showed statistically significant and clear-cut precise latitudinal cline in the missense nucleotide variations, and which are also well characterized in the literature. The nine candidate genes do not belong to a single pathway. Therefore, in this article, we have discussed the probable pathways separately that involve the candidate genes and link them wherever it was applicable. While doing so, we referred to the earlier work carried out in the context of a particular gene.

**Table 1:** Differentially expressed genes in response to SHADE in the southern and northern Norway spruce populations, which showed latitudinal cline in SNPs of the genes differentially regulated under SHADE

Gene name	Spruce gene id	TAIR id	Expression	Variation in nucleotide	Function	Reference
*MYB DOMAIN PROTEIN 3* (*MYB3*)	MA_7115g0010	AT1G22640	SHADE-67 < SHADE-56	T132R: Reference C, alternate G ACA(ACA, Thr, T)→AGA(AGA, Arg, R) Polar hydrophilic neutral→ Polar hydrophilic basic	Transcription factor—represses phenylpropanoid biosynthesis	Zhou et al. (2017), Deng and Lu (2017)
*LONG VEGETATIVE PHASE 1* (*LOV1*)	MA_16619g0010	AT2G02450	SHADE-67 > SHADE-56	R156K: Reference G, alternate A AGG(AGG, Arg, R)→AAG(AAG, Lys, K) Polar hydrophilic basic→ Polar hydrophilic basic D190N: Reference G, alternate A GAT(GAU, Asp, D)→AAT(AAU, Asn, N) Polar hydrophilic acidic→ Polar hydrophilic neutral	Transcription factor—regulates flowering time and lignin content, cold tolerance	Xu et al. (2012), Yoo et al. (2007)
*SCREAM2* (*SCRM2*)	MA_10435231g0010	AT1G12860	SHADE-67 < SHADE-56	T208M: Reference C, alternate T ACG(ACG, Thr, T)→ ATG(AUG, Met, M) Polar hydrophilic neutral→ Non-polar hydrophobic S238N: Reference G, alternate A AGT(AGU, Ser, S)→ AAT(AAU, Asn, N) Polar hydrophilic neutral→ Polar hydrophilic neutral	Transcription factor—regulates stomatal development and involved in cold acclimation	Kanaoka et al. (2008), Fursova et al. (2009)
*TEOSINTE BRANCHED 1*, *CYCLOIDEA AND PCF TRANSCRIPTION FACTOR 2* (*TCP2*)	MA_92659g0010	AT4G18390	SHADE-67 < SHADE-56	F4S: Reference T, alternate C TTT(UUU, Phe, F)→TCT(UCU, Ser, S) Non-polar hydrophobic→ Polar hydrophilic neutral V25A: Reference T, alternate C GTA(GUA, Val, V)→ GCA(GCA, Ala, A) Non-polar hydrophobic→ Non-polar hydrophobic E32G: Reference A, alternate G GAG(GAG, Glu, E)→ GGG(GGG, Gly, G) Polar hydrophilic acidic→ Non-polar hydrophobic E51K: Reference G, alternate A GAA(GAA, Glu, E)→ AAA(AAA, Lys, K) Polar hydrophilic acidic→ Polar hydrophilic basic I53F: Reference A, alternate T ATT(AUU, Ile, I)→ TTT(UUU, Phe, F) Non-polar hydrophobic→→ Non-polar hydrophobic	Transcription factor—promotes light-regulated transcription of HY5, HYH; positively regulates photomorphogenesis	He et al. (2016)
*NAC DOMAIN CONTAINING PROTEIN 36* (*NAC036*/*ANAC036*)	MA_101849g0010	AT2G17040	SHADE-67 > SHADE-56	D206E: Reference T, alternate A GAT(GAU, Asp, D)→GAA(GAA, Glu, E) Polar hydrophilic acidic→ Polar hydrophilic acidic T237M: Reference C, alternate T ACG(ACG, Thr, T)→ ATG (AUG, Met, M) Polar hydrophilic neutral→ Non-polar hydrophobic	Transcription factor—involved in leaf and inflorescence stem morphogenesis	Kato et al. (2010)
*EXPANSIN B3* (*EXPB3*)	MA_7354451g0010	AT4G28250	SHADE-67 > SHADE-56	K3R: Reference A, alternate G AAG(AAG, Lys, K)→ AGG(AGG, Arg, R) Polar hydrophilic basic→ Polar hydrophilic basic R6S: Reference G, alternate T AGG(AGG, Arg, R)→ AGT(AGU, Ser, S) Polar hydrophilic basic→ Polar hydrophilic neutral C32R: Reference T, alternate C TGC(UGC, Cys, C)→CGC (CGC, Arg, R) Polar hydrophilic neutral→ Polar hydrophilic basic	Cell wall loosening—pH depended, non-enzymatic Internode elongation and root development	Marowa et al.( 2016)
*FCS LIKE ZINC FINGER 6* (*FLZ6*)	MA_14341g0010	AT1G78020	SHADE-67 < SHADE-56	L88V: Reference C, alternate G CTG(CUG, Leu, L)→ GTG(GUG, Val, V) Non-polar hydrophobic→ Non-polar hydrophobic	Plays role in plant growth by repressing the SnRK1 signaling cascade	Jamsheer et al. (2018), Jamsheer and Laxmi (2015)
*VASCULAR-RELATED RLK 1* (*VRLK1*)	MA_587505g0010	AT1G79620	SHADE-67 < SHADE-56	A265V: Reference C, alternate T GCT(GCU, Ala, A)→GTT(GUU, Val, V) Non-polar hydrophobic→ Non-polar hydrophobic V283L: Reference G, alternate C GTG(GUG, Val, V)→ CTG(CUG, Leu, L) Non-polar hydrophobic→ Non-polar hydrophobic	Involved in switching between cell elongation and secondary cell wall thickening	Huang et al. (2018)
*RESISTANT TO P. SYRINGAE 2* (*RPS2*)	MA_475302g0010	AT4G26090	SHADE-67 > SHADE-56	S123N: Reference G, alternate A AGT(AGU, Ser, S)→AAT(AAU, Asn, N) Polar hydrophilic neutral→ Polar hydrophilic neutral E166A: Reference A, alternate C GAA(GAA, Glu, E)→ GCA(GCA, Ala, A) Polar hydrophilic acidic→ Non-polar hydrophobic	Involved in disease resistance	Kunkel et al. (1993)

**Fig. 2:**
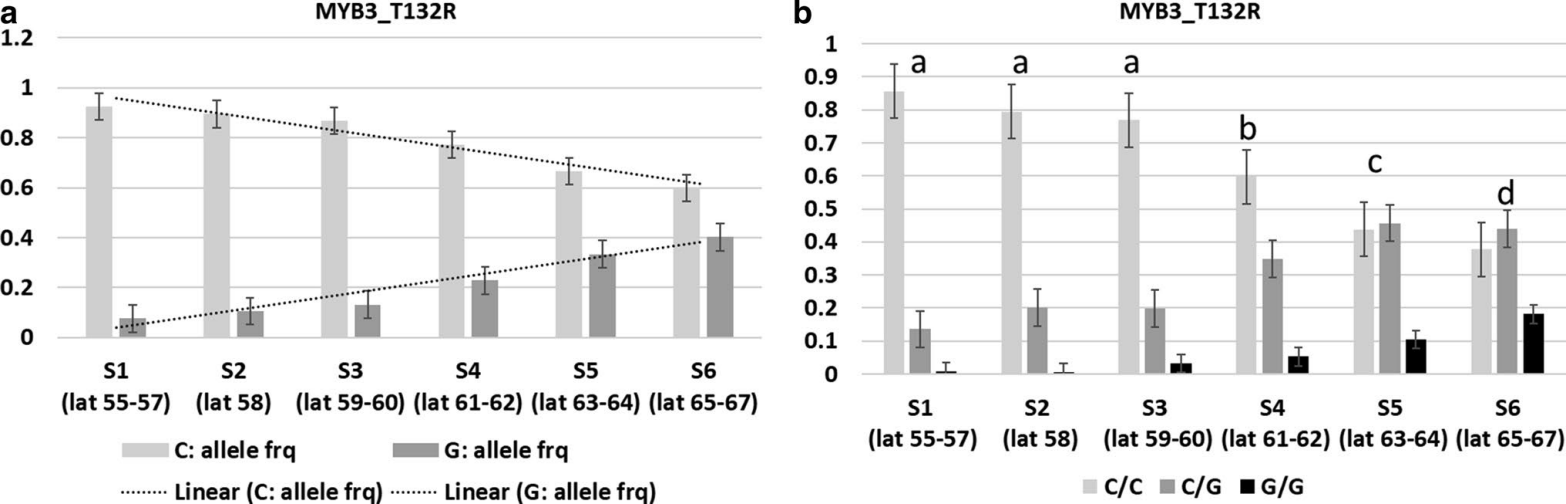
Cline with reference to variation in allele and genotype frequencies of SNP in the *MYB3* gene in Norway spruce populations across Sweden. **a** Allele frequencies of T132R. **b** Genotype frequencies of T132R, Tukey’s post-hoc categorization is indicated above the bars

The allele frequencies and genotype frequencies of missense SNPs in nine DEGs followed a latitudinal cline which included five transcription factors involved in photomorphogenesis (*TCP2*), lignin pathway (*MYB DOMAIN PROTEIN 3*, *MYB3*; *LONG VEGETATIVE PHASE 1*, *LOV1*), stomatal development, and cold acclimation (*SCREAM 2*, *SCRM2*) and, leaf and inflorescence stem morphogenesis (*NAC DOMAIN CONTAINING PROTEIN 36*, *NAC036*). Other DEGs that showed clinal variation in sequence variations were – *EXPANSIN B3* (*EXPB3*) that controls the cell wall remodeling, *FCS LIKE ZINC FINGER 6* (*FLZ6*) which is involved in response to starvation, and *VASCULAR-RELATED RLK 1* (*VRLK1*) that regulates secondary cell wall thickening and a gene potentially involved in plant immunity [*RESISTANT TO P. SYRINGAE 2* (*RPS2*)] (Tables 1, 2, Fig. 2, Supplementary Fig. S7–S14).

**Table 2:** Population-wise allele frequency of missense SNPs showing latitudinal cline in candidate genes differentially (DEGs) regulated under SHADE

Gene name	SNP	Allele	Population-wise allele frequency					
			S1	S2	S3	S4	S5	S6
LOV1	R156K	Reference (G)	0.7	0.65	0.68	0.68	0.65	0.53
		Alternate (A)	0.3	0.35	0.32	0.32	0.35	0.47
LOV1	D190N	Reference (G)	0.33	0.38	0.35	0.41	0.45	0.56
		Alternate (A)	0.67	0.62	0.65	0.59	0.55	0.44
SCRM2	T208M	Reference (C)	0.21	0.24	0.31	0.36	0.44	0.5
		Alternate (T)	0.79	0.76	0.69	0.64	0.56	0.5
SCRM2	S238N	Reference (G)	0.19	0.17	0.28	0.34	0.44	0.48
		Alternate (A)	0.81	0.83	0.72	0.66	0.56	0.52
TCP2	F4S	Reference (T)	0.91	0.84	0.79	0.69	0.67	0.52
		Alternate (C)	0.09	0.16	0.21	0.31	0.33	0.48
TCP2	V25A	Reference (T)	0.87	0.91	0.92	0.95	0.96	0.97
		Alternate (C)	0.13	0.09	0.08	0.05	0.04	0.03
TCP2	E32G	Reference (A)	0.87	0.91	0.92	0.95	0.96	0.97
		Alternate (G)	0.13	0.09	0.08	0.05	0.04	0.03
TCP2	E51K	Reference (G)	0.71	0.73	0.83	0.9	0.93	0.9
		Alternate (A)	0.29	0.27	0.18	0.1	0.07	0.1
TCP2	I53F	Reference (A)	0.71	0.74	0.82	0.9	0.93	0.9
		Alternate (T)	0.29	0.26	0.18	0.1	0.07	0.1
NAC036	D206E	Reference (T)	0.98	0.97	0.95	0.92	0.9	0.89
		Alternate (A)	0.02	0.03	0.05	0.08	0.1	0.11
NAC036	T237M	Reference (C)	0.87	0.85	0.85	0.84	0.82	0.79
		Alternate (T)	0.13	0.15	0.15	0.16	0.18	0.21
EXPB3	K3R	Reference (A)	0.37	0.37	0.34	0.26	0.25	0.22
		Alternate (G)	0.63	0.63	0.66	0.74	0.75	0.78
EXPB3	R6S	Reference (G)	0.46	0.42	0.4	0.31	0.27	0.24
		Alternate (T)	0.54	0.58	0.6	0.69	0.73	0.76
EXPB3	C32R	Reference (T)	0.95	0.96	0.98	0.98	0.99	0.99
		Alternate (C)	0.05	0.04	0.02	0.02	0.01	0.01
FLZ6	L88V	Reference (C)	0.39	0.4	0.51	0.51	0.55	0.57
		Alternate (G)	0.61	0.6	0.49	0.49	0.45	0.43
VRLK1	A265V	Reference (C)	0.88	0.89	0.86	0.90	0.93	0.92
		Alternate (T)	0.12	0.11	0.14	0.10	0.07	0.08
VRLK1	V283L	Reference (G)	0.95	0.95	0.94	0.87	0.87	0.87
		Alternate (C)	0.05	0.05	0.06	0.13	0.13	0.13
RPS2	S123N	Reference (G)	0.13	0.10	0.26	0.39	0.41	0.44
		Alternate (A)	0.87	0.90	0.74	0.61	0.59	0.56
RPS2	E166A	Reference (A)	0.16	0.15	0.30	0.44	0.41	0.44
		Alternate (C)	0.84	0.85	0.70	0.56	0.59	0.56

The allele frequencies of the missense SNPs for the nine candidate genes were found to be significantly different across all the populations included in the study except in the case of a few SNPs; however, the genotype frequencies were observed to be significantly different in all populations (Supplementary Table S3), which were further subjected to Tukey’s *post-hoc* (Supplementary Table S4). The location of the missense mutations in the nine candidate genes with reference to a particular domain of the respective gene is included in the Supplementary Material (Supplementary Figs. S15–S23).

**Fig. 3:**
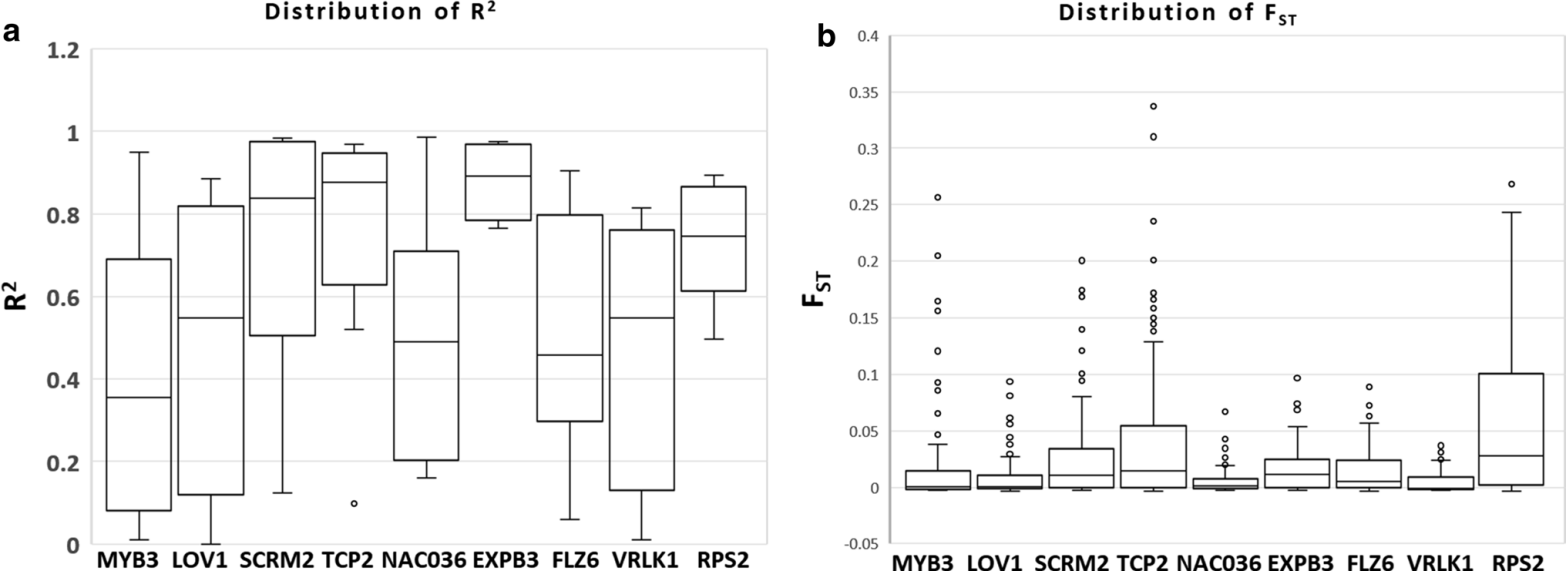
The distribution of **a** *R*^2^ with reference to allele frequencies and **b** *F*_*ST*_, across the nine candidate genes showing clines in allele frequencies and which are differentially regulated in response to SHADE in southern and northern Norway spruce populations Sweden. *R*^2^ and *F*_*ST*_ were calculated considering the missense and the synonymous SNPs detected in the particular candidate gene

**Fig. 4:**
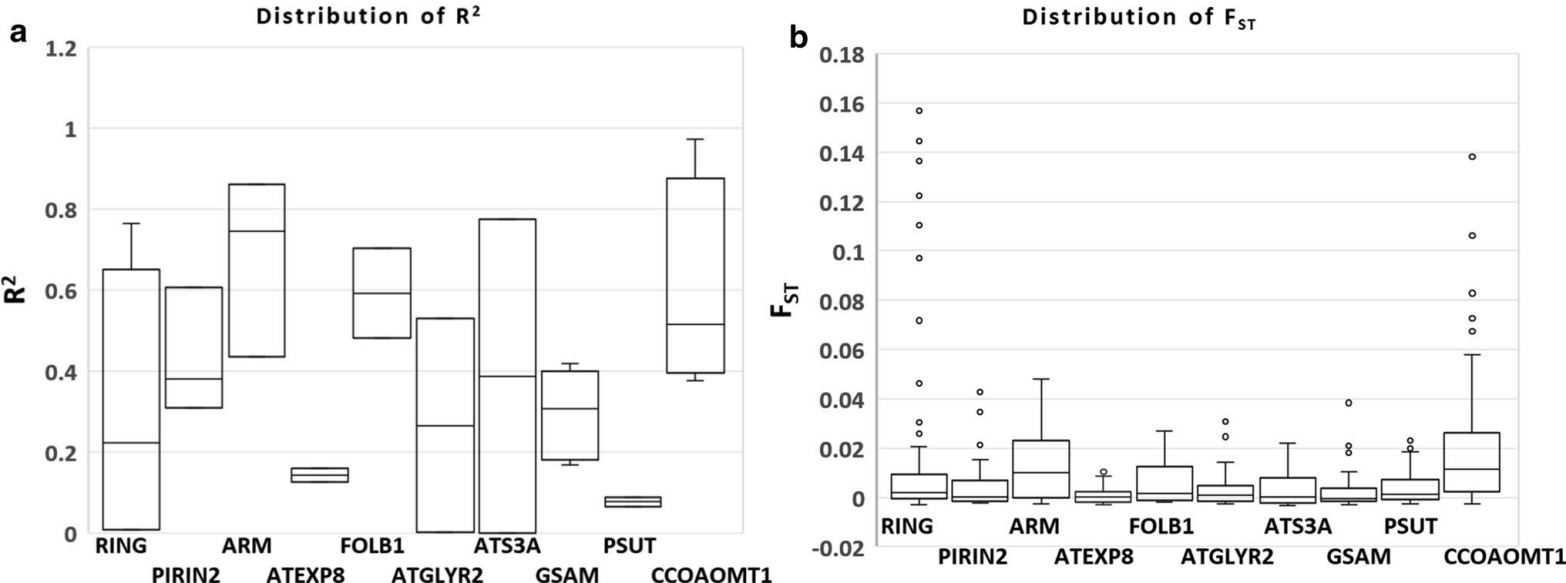
The distribution of **a** *R*^2^ with reference to allele frequencies and **b** *F*_*ST*_, across the ten control genes which are not differentially regulated in response to SHADE in southern and northern Norway spruce populations Sweden. *R*^2^ and *F*_*ST*_ were calculated considering the missense and the synonymous SNPs detected in the particular control gene

The majority of the loci were found to be in Hardy Weinberg Equilibrium (HWE) with reference to the missense SNPs detected in the DEGs, with few exceptions in the case of *LOV1* (D190N), *TCP2* (F4S), *TCP2* (T518K), *EXPB3* (R6S), *FLZ6* (L88V) and *VRLK1* (V283L) where a deviation from HWE was observed (Supplementary Table S5). Pairwise *F*_*ST*_ estimates for all the six populations, calculated considering both the synonymous + missense SNPs of the 54 DEGs (Table 3) and the control genes that were not differentially regulated under SHADE (Table 4), suggest that differentiation increases with the geographic distance. 24 missense mutations in the nine candidate DEGs in response to SHADE in Norway spruce did not show cline (Supplementary Table S6). Mild clines across latitudes were also observed in eight synonymous substitutions with reference to the nine candidate genes: TCP2 (two SNPs)*, LOV1* (one SNP)*, FLZ6* (one SNP), and *NAC036* (four SNPs) (Supplementary Table S7). Cline was not observed in ten synonymous substitutions in the nine candidate genes (Supplementary Table S8). The distribution of *R*^2^ and *F*_*ST*_ across the nine candidate genes (DEGs) and the ten control genes (non-DEGs) is represented in Figs. 3, 4, respectively. We have included the details regarding the allele frequencies of the missense mutations in the DEGs other than the nine candidate genes in the supplementary material (Supplementary Table S9). We have considered all the 54 genes to estimate genetic diversity across the populations included in the study. These genes were differentially regulated in response to SHADE and therefore might be novel regulators in this context. Gene expression of the ten non-DEGs (controls) in response to SHADE in Norway spruce is included in Supplementary Table S10 and respective allele frequencies of the ten non-DEGs (controls) are included in Supplementary Table S11.

**Table 3:** Pairwise *F*_*ST*_ estimates for six populations across Sweden involved in the analysis for detection of latitudinal clines in allele frequencies of the SNPs of the genes differentially regulated under SHADE (synonymous + missense SNPs of the 54 DEGs)

Population	S2	S3	S4	S5	S6
S1	0.00	0.003034	0.020345	0.006756	0.028848
S2		0.001117	0.00528	0.000451	0.025112
S3			0.00	0.00	0.00828
S4				0.000412	0.00
S5					0.001509

**Table 4:** Pairwise *F*_*ST*_ estimates for six populations across Sweden involved in the analysis for detection of latitudinal clines in allele frequencies of the SNPs of the control genes (non- differentially regulated) under SHADE (synonymous + missense SNPs of the ten non-DEGs)

Population	S2	S3	S4	S5	S6
S1	0.00	0.00186	0.00453	0.00143	0.00155
S2		0.00	0.00175	n.a	0.00224
S3			0.00	0.00918	0.00355
S4				n.a	n.a
S5					0.0005

### Types of the missense polymorphisms and alteration in protein conformation in the nine candidate genes

The majority of the missense polymorphisms in the candidate genes resulted in alteration of the amino acid with similar chemical property (e.g. polar/hydrophilic to polar/hydrophilic), with a few exceptions (Table 1). MYB3, LOV1, EXPB3, FLZ6, and VRLK1 showed alterations to an amino acid with similar chemical properties whereas one of the polymorphisms in SCRM2, NAC036 and RPS2, and two in TCP2 showed changes in the amino acids that resulted in a change in the chemical property, e.g. from polar/hydrophilic to non-polar/hydrophobic and vice-versa.

The missense polymorphism T208M in the *SCRM2* gene involves a change in the amino acid from polar/hydrophilic to non-polar/hydrophobic similar to that of the polymorphisms in *TCP2* (E32G), *NAC036* (T237M) and *RPS2* (E166A) (Table 1). Likewise, there is a change in the amino acid from non-polar/hydrophobic to polar/hydrophilic in *TCP2* gene (F4S). These polymorphisms are likely to alter the protein folding and conformation, consequently influencing their binding-capacity/interaction, leading to an alteration in their mode of action. Although the missense polymorphisms reported in this study were not located in the specific conserved domains of the genes (Supplementary Figs. S15–S23), e.g. the DNA binding domain of the particular transcription factor, it will still affect the mode of action of the protein by altering its conformation.

### Latitudinal clines in the allele and genotype frequencies of the DEGs

Pairwise *F*_*ST*_ estimates (Table 3) increase with the geographic (latitudinal) distance between populations, although in general, the values are low, which agree with previous reports in Norway spruce (Chen et al. 2016). This trend corresponds to a significant latitudinal change in the allele/genotype frequencies of missense polymorphisms that suggests the involvement of the DEGs in the population’s adaptation to the local light conditions. The synonymous SNPs detected in the DEGs and, the missense and synonymous SNPs in the non-DEGs did not show significant cline. Our results together with previous studies in population differentiation in Norway spruce suggest that natural selection and to a less extent isolation by distance (Chen et al. 2014) to be the main force shaping the observed latitudinal cline at the DEGs. As compared to previous studies (Pyhajarvi et al. 2007; Chen et al. 2014, 2016; Kallman et al. 2014), we applied transcriptomic analysis to identify potential candidate genes to explain the genetic basis of latitudinal adaptation. It is relevant to highlight that exome capture does not aim to sequence the entire coding sequence of the gene; therefore, it is possible that we have missed some SNPs that are under selection for the simple reason that they are located in the non-sequenced exons. Moreover, some of the neutral SNPs showing a latitudinal cline may be in linkage disequilibrium with SNPs under selection and therefore, they may appear associated to the adaptive cline. The four genes with the highest *F*_*ST*_ values (*SCRM2, TCP2, EXPB3, RPS2*) also exhibit higher and less dispersed R^2^ values as a result of a steeper latitudinal cline (Fig. 3). As expected from the global *F*_*ST*_ estimates (including 54 DEGs, Table 3), the *F*_*ST*_ values are low and show different levels of dispersion upwards, which can be explained by an increase in differentiation with pair-wise geographic distance.

Multiple studies in Scots pine and Norway spruce support a very low population genetic structure based on the highly polymorphic microsatellite or SSR markers (Belletti et al. 2012; Androsiuk et al. 2013; Wójkiewicza and Wachowiak 2016; Stojnić et al. 2019). These studies have reported low *F*_*ST*_. A low value of *F*_*ST*_ indicates low population genetic differentiation, which in the case of conifers can be accounted by an efficient gene (pollen) flow. The low *F*_*ST*_ values for Scots pine and Norway spruce populations contrast with a high population differentiation at traits of adaptive value such as the timing of budburst or timing of bud-set (Savolainen et al. 2004; Notivol et al. 2007). This scenario supports that the two conifer species form excellent experimental systems to genetically dissect complex traits of adaptive value. The genes that follow a latitudinal cline (R^2^ higher than 0.8 for SNP frequency variation) also shown higher *F*_*ST*_ values. Otherwise, *F*_*ST*_ values are low which agrees with previous reports in conifers. Likewise, the control genes also showed low *F*_*ST*_ values (Table 4, Fig. 4), although the SNPs detected in the control genes do not follow a latitudinal cline.

### Cell wall and lignin pathway

Comparison of the expression pattern (RNA-Seq) under SHADE treatment between the southern and northern Norway spruce populations where SUN was used as control treatment led to the identification of DEGs in response to SHADE. Clines were observed with reference to variation in allele and genotype frequencies of SNPs in nine DEGs, of which two genes represented transcription factors related to lignin, *MYB3* involved in lignin biosynthesis (Zhou et al. 2017) and *LOV1* which regulates flowering time and lignin content and composition (Xu et al. 2012). MYB3 represses the cinnamate 4-hydroxylase (C4H) gene expression, which catalyzes the second step of the main phenylpropanoid pathway leading to the synthesis of lignin and pigments (Liu et al. 2018). *LOV1* controls flowering time within the photoperiod pathway in *Arabidopsis thaliana* by negatively regulating the expression of *CONSTANS* (*CO*) which is a floral promoter (Yoo et al. 2007). Overexpression of *LOV1* leads to delayed flowering time and increased lignin content in switch-grass (Xu et al. 2012). One gene involved in cell wall synthesis (*VRLK1*) also showed clinal variation in the detected SNPs, while the other two genes—*CESA4* and *CCR2* were differentially regulated under SHADE, although they did not show clinal variation with reference to SNPs. *VRLK1* encodes a leucine-rich repeat receptor-like kinase which is expressed specifically in cells undergoing secondary cell wall thickening (Torii 2004). The secondary cell wall contains additional lignin as compared to the primary cell wall. *VRLK1* regulates secondary cell wall thickening; down-regulation of *VRLK1* promotes secondary cell wall thickening and up-regulation of *VRLK1* inhibits it in *A. thaliana* (Huang et al. 2018). *CESA4* is up-regulated in the northern trees, suggesting enhanced secondary cell wall biosynthesis. In the context of differential expression between the southern and northern Norway spruce populations in response to SHADE, *MYB3* was down-regulated at latitude 67^o^2´N as compared to latitude 56^o^2´N and *LOV1* was found to be up-regulated at latitude 67^o^2´N as compared to latitude 56^o^2´N. Furthermore, up-regulation of *CCR2* at latitude 67^o^2´N supports the increase in lignin biosynthesis as suppression of CCR results in a reduction in lignin content in Norway spruce (Wadenback et al. 2008). This suggests that the lignin pathway was enhanced at latitude 67^o^2´N as compared to latitude 56^o^2´N under SHADE, which may be co-related with the SNPs detected in the respective DEG. Additionally, *VRLK1* expression was lower in the northern trees than the southern ones under SHADE, which again indicates that secondary cell wall thickening (lignin deposition) was enhanced in the northern populations. A higher number of cell wall genes were up-regulated at latitude 67^o^2´N (Supplementary Fig. S2). This phenomenon can be attributed due to the variation in the SNPs in the respective genes observed in this study that follows a latitudinal cline across Sweden. Although shade stress decreases lignin leading to weak stems in many of the plant species (Wang et al. 2012; Wu et al. 2017; Hussain et al. 2019; Liu et al. 2019b), the northern Norway spruce populations appear to have adapted to SHADE (or low R:FR ratio) or higher requirement of FR to maintain the growth and other regular plant processes (Clapham et al. 1998, 2002; Ranade and García-Gil 2013), which may correspond to the observed up-regulation of cell wall-related genes. It is worth mentioning that none of the populations deviated from Hardy Weinberg Equilibrium (HWE) in case of *MYB3*, which suggests that the particular genetic variation in *MYB3* gene (T132R) will remain constant (i.e., not evolving) in the entire population from one generation to the next in absence of disturbing forces.

### Cold tolerance

*SCRM2* encodes *INDUCER OF CBF EXPRESSION 2* (*ICE2*) which participates in the response to deep freezing; overexpression of *ICE2* results in increased tolerance to deep freezing stress after cold acclimation (Fursova et al. 2009). Gain of function of *LOV1* gene confers cold tolerance in *A. thaliana* (Yoo et al. 2007). *SCRM2* was down-regulated and *LOV1* was up-regulated in response to SHADE at latitude 67^o^2´N as compared to latitude 56^o^2´N, and this coupled with the clinal variation in the respective gene sequences conveys that the trees at different latitudes may have different molecular mechanisms to adapt to the cold environmental conditions prevailing in the northern latitudes.

### Photomorphogenesis and bud-burst

*TCP2* is a transcription factor that positively regulates photomorphogenesis; it promotes light-regulated transcription of photomorphogenesis-related genes e.g. *ELONGATED HYPOCOTYL 5* (*HY5*) and *HY5* homolog (*HYH*) in *A. thaliana* (He et al. 2016). *TCP2* controls cell-proliferation/division and growth, including floral meristem (Martin-Trillo and Cubas 2010). *TCP2* is also associated with bud-burst, it was found to be up-regulated from quiescent bud to burst bud in tea (Liu et al. 2019a). *TCP2* was detected to be down-regulated at latitude 67^o^2´N as compared to latitude 56^o^2´N in response to SHADE, suggesting that SHADE diminishes cell-division related activities in northern tree populations as compared to the southern ones; instead, the northern trees seem to invest higher resources in cell wall thickening. This might influence the timing of bud-burst and bud-set under SHADE, the two traits that are known to follow a steep latitudinal cline in Norway spruce (Sogaard et al. 2008; Chen et al. 2012). Considering that bud-set and bud-burst are known to be closely linked responses and that the northern trees are more frost resistant (Westin et al. 2000; Calleja-Rodriguez et al. 2019; Sebastian-Azcona et al. 2019), it warrants further research to verify the possible role of the interaction between phenology and cold tolerance related DEGs in adaptation to light quality.

### Stem development

*NAC036* is a transcription factor that regulates leaf and inflorescence stem morphogenesis. Overexpression of NAC036 resulted in a dwarf phenotype in *A. thaliana* (Kato et al. 2010). This gene was found to be associated with the shade-tolerant response in Norway spruce where it was reported to be up-regulated under SHADE conditions (Ranade et al. 2019). In the current work, *NAC036* was up-regulated in response to SHADE in the northern trees as compared to the southern ones. Expansins comprise of a large gene family that codes for cell wall proteins, which mediate the pH-depended, non-enzymatic cell wall loosening and extension that plays a vital role in plant cell growth and development (Marowa et al. 2016). Differential expression of three expansins in response to SHADE was detected in both populations. *EXPANSIN-LIKE A1* (*EXLA1*) and *EXPANSIN A8* (*EXPA8*) were up-regulated at latitude 56^o^2´N as compared to 67^o^2´N, and *EXPB3* was up-regulated at latitude 67^o^2´N as compared to latitude 56^o^2´N (Supplementary Table S1-S2). Out of these, only *EXPB3* showed clinal variation in the sequence. *EXPB3* in rice has been reported to be involved in stem/internode elongation as well as root development (Marowa et al. 2016). Thus, genes responsible for stem development were detected to be differentially regulated in response to SHADE in both populations.

### Regulation of plant growth

Biotic and abiotic stresses lead to energy deficit in the plant cell that triggers the transcription of genes enabling the plants to withstand and survive under low-energy conditions. The SNF1-RELATED KINASE1 (*SnRK1*) signalling cascade is activated to combat low-energy often by stopping the plant growth. This involves a reduction in ribosomal protein synthesis, and a simultaneous accumulation of protective metabolites or defense compounds coupled with the tuning of the metabolic processes in response to starvation (Baena-Gonzalez et al. 2007; Wurzinger et al. 2018). The *SnRK1* signalling cascade involves the interaction of *FLZ* gene family members with the kinase subunits. Expression of *FLZ* genes is both positively and negatively regulated by energy deficit as well as energy-rich conditions (Jamsheer and Laxmi 2015). Starvation or low energy induces expression of *FLZ6*, *SnRK1* also induces *FLZ6* during energy starvation. Through repression of *SnRK1*, *FLZ6* promotes the target of the rapamycin or TOR signalling pathway that induces growth in favourable conditions. *FLZ6* mutants show inhibition and reduced seedling growth under favourable growth conditions due to enhanced *SnRK1* activity that confirms the role of *FLZ6* in plant growth by regulating *SnRK1* signalling (Jamsheer et al. 2018). *FLZ6* was found to be up-regulated under SHADE at latitude 67^o^2´N as compared to latitude 56^o^2´N, which may indicate that the trees in the northern latitudes are better adapted to shade conditions by withstanding the shade stress and continue to grow better compared to the southern populations.

### Shade responsive/related genes

Norway spruce is a shade-tolerant conifer species. It is interesting to mention that in our earlier work we reported that *SCRM2*, *NAC036,* and *FLZ6* were the genes responsible for the shade-tolerant response in Norway spruce (Ranade et al. 2019). Our current study identified a number of missense polymorphisms in these genes that follow a latitudinal cline in response to SHADE. *PAR2* is the shade responsive gene that was up-regulated under SHADE, in the northern population. *DRA2* and *EXPB3*, genes involved in hypocotyl and stem/internode elongation respectively, were up-regulated in the northern population. *DRA2* along with *EXPB3* may contribute to cell elongation, leading to elongation of the hypocotyls in the northern seedlings, which are the longest in response to SHADE among all four latitudes. It may be concluded that although Norway spruce is a shade-tolerant species, yet there is a difference in the hypocotyl elongation in response to the shade that follows a latitudinal cline.

### Plant defense

Up-regulation of a higher number of defense-related genes was observed in the northern population under SHADE. Likewise, the MapMan network indicates higher expression of genes at latitude 67^o^2´N that are included in the phenylpropanoid pathway involved in defense (Deng and Lu 2017) (Supplementary Fig. S2-S4). MYB3 represses phenylpropanoid biosynthesis and down-regulation of *MYB3* (with cline in SNP) in the north indicates that the phenylpropanoid pathway is enhanced at latitude 67^o^2´N. Enhanced secondary cell wall biosynthesis is indicated by the up-regulation of *CESA4* in the northern latitude. Cell wall plays an active role in plant immunity (Bacete et al. 2018). Lignin forms a barrier and restricts pathogen entry thus conferring disease resistance in plants (Lee et al. 2019). Plant immunity specific genes *RPS2* and *LECRK-S.4,* were detected to be up-regulated in the northern trees where *RPS2* also showed a latitudinal cline in SNPs. We propose that northern trees might be better equipped for disease-resistant mechanisms under shade as compared to the southern ones as inferred from the RNA-Seq analysis and the exome capture data. However, further experiments are needed to confirm the details of the molecular basis and underlined physiology of the phenomenon.

## Conclusions

Understanding adaptation to local climatic conditions is one of the crucial factors in forest breeding and conservation. Our study on natural variation along a latitudinal cline has provided insights into the genomic basis for local adaptation to shade in Norway spruce—one of the most economically important conifer tree species in Sweden. Integration of knowledge on local adaption into forest tree breeding programs aims for sustainable forestry. Climate change would lead to an increase in the mean temperatures especially in the northern latitudes, but it will have no effect or negligible effect on the light quality. Therefore, in the context of climate change, populations in the northern latitudes will continue to receive a higher amount of FR light or low R:FR ratio that is equivalent to shade, as well as the absence of night during their entire growth season. Our results would contribute to the efficient design of programs for assisted migration as a solution to mitigate the mal-adaptation of present populations to their local conditions as a consequence of climatic change.

### *Author contribution statement*

SSR contributed to experiment performance, data collection, data analysis and interpretation, and manuscript writing. MRGG contributed to experimental design, data analysis and interpretation, and manuscript writing. Both authors read and approved the manuscript.

## Supplementary Information

Below is the link to the electronic supplementary material.

Supplementary file1 (PDF 2367 KB)

## Abbreviations

DEGs: Differentially expressed genes
EOD: End-of-day
EXPB3: EXPANSIN B3
FLZ6: FCS LIKE ZINC FINGER 6
LOV1: LONG VEGETATIVE PHASE 1
MYB3: MYB DOMAIN PROTEIN 3
NAC036: NAC DOMAIN CONTAINING PROTEIN 36
RFR: Red:Far-red ratio
RPS2: RESISTANT TO P. SYRINGAE 2
SCRM2: SCREAM 2
TCP2: CYCLOIDEA AND PCF TRANSCRIPTION FACTOR 2
VRLK1: VASCULAR-RELATED RLK 1
