# Supplementary material for: Molecular signatures of local adaptation to light in Norway spruce
Source: Planta. 2021 Jan 28;253(2):53. doi: 10.1007/s00425-020-03517-9 (PMC7843583; doi:10.1007/s00425-020-03517-9)
Supplement: Supplementary file 1 — Supplementary file1 (PDF 2367 KB) [file 425_2020_3517_MOESM1_ESM.pdf]

The explanations for all the columns in the output of the differential analysis in Table S1 and Table S2 is as follows from the DESeq2 manual. The columns include: baseMean = the average of the normalized counts taken over all samples; log2FoldChange = log2 fold change between the groups. E.g. value 2 means that the expression has increased 4-fold; lfcSE = standard error of the log2FoldChange estimate; stat = Wald statistic; pvalue = Wald test p-value; padj = Benjamini-Hochberg adjusted p-value

Table S1 Genes for which the levels of expression were significantly higher in the 67°N population as compared to the 56°N population in response to SHADE in Norway spruce

Gene_ID	TAIR	Primary Gene Symbol	Gene Model Description	Expression	baseMean	log2FoldChange	lfcSE	stat	pvalue	padj
MA_93016g0010	AT1G01490		Heavy metal transport/detoxification superfam	shade67>shade56	171.99156	19.31080501	3.3123768	5.8298938	5.55E-09	4.05E-06
MA_10436915g0010	AT1G01630		Sec14p-like phosphatidylinositol transfer famil	shade67>shade56	181.74323	0.890249942	0.2256632	3.9450378	7.98E-05	0.00785557
MA_9801637g0010	AT1G02080		transcription regulator;(source:Araport11)	shade67>shade56	213.33803	24.54497744	4.3990602	5.5795957	2.41E-08	1.52E-05
MA_11144g0010	AT1G02205	ECERIFERUM 1 (CER1)	Expression of the CER1 gene associated with p	shade67>shade56	108.46666	2.068137632	0.5135865	4.0268533	5.65E-05	0.00616581
MA_10107572g0010	AT1G02800	CELLULASE 2 (CEL2)	Encodes a protein with similarity to endo-1,4-b	shade67>shade56	139.88385	8.882139059	1.4882152	5.9683163	2.40E-09	1.91E-06
MA_4984597g0010	AT1G03230		Eukaryotic aspartyl protease family protein;(so	shade67>shade56	411.38787	21.9613859	4.8673892	4.5119436	6.42E-06	0.00109619
MA_388691g0010	AT1G03600	(PSB27)	PSB27 is a chloroplast lumen localized protein	shade67>shade56	742.76598	0.648168009	0.1545924	4.192755	2.76E-05	0.00353428
MA_45121g0010	AT1G05670		Pentatricopeptide repeat (PPR-like) superfamil	shade67>shade56	184.58984	23.3255113	4.6756254	4.9887468	6.08E-07	0.00015401
MA_5724086g0010	AT1G10170	NF-X-LIKE 1 (NFXL1)	Encodes AtNFXL1, a homologue of the putative	shade67>shade56	39.044387	21.84528163	5.5538932	3.9333277	8.38E-05	0.00803983
MA_12361g0010	AT1G10390	DRACULA2 (DRA2)	DRA2 is a homolog of mammalian nucleoporin	shade67>shade56	95.148831	2.635868046	0.5869981	4.4904199	7.11E-06	0.00116898
MA_640601g0010	AT1G10640		Pectin lyase-like superfamily protein;(source:A	shade67>shade56	120.66502	1.686576144	0.3424242	4.9253994	8.42E-07	0.00020244
MA_10430253g0010	AT1G10740		alpha/beta-Hydrolases superfamily protein;(so	shade67>shade56	43.357063	19.20864968	4.2782117	4.4898782	7.13E-06	0.00116898
MA_10432704g0020	AT1G11290	CHLORORESPIRATORY REDUCTI	Pentatricopeptide Repeat Protein containing t	shade67>shade56	388.93827	2.036870563	0.4773711	4.2668492	1.98E-05	0.00270169
MA_500971g0010	AT1G12050	FUMARYLACETOACETATE HYDR	Encodes a fumarylacetoacetase that converts	shade67>shade56	137.11908	30	6.0065614	4.9945381	5.90E-07	0.00015401
MA_10435025g0010	AT1G13520		hypothetical protein (DUF1262);(source:Arapo	shade67>shade56	139.92554	2.605127449	0.6168562	4.223233	2.41E-05	0.00318496
MA_15382g0010	AT1G13570		F-box/RNI-like superfamily protein;(source:Ara	shade67>shade56	63.129848	19.75701806	4.5405589	4.3512304	1.35E-05	0.00200323
MA_179350g0020	AT1G23740	ALKENAL/ONE OXIDOREDUCTAS	AOR is an alkenal/one oxidoreductase that act	shade67>shade56	467.58936	25.98001962	4.9570437	5.2410309	1.60E-07	7.51E-05
MA_470965g0010	AT1G25440	B-BOX DOMAIN PROTEIN 15 (BE	B-box type zinc finger protein with CCT domair	shade67>shade56	455.31386	1.121153225	0.2626287	4.2689661	1.96E-05	0.00268966
MA_89572g0010	AT1G26760	SET DOMAIN PROTEIN 35 (SDG3	SET domain protein 35;(source:Araport11)	shade67>shade56	42.735581	20.65022444	4.4404534	4.6504765	3.31E-06	0.00060152
MA_129800g0010	AT1G27170		transmembrane receptors / ATP binding protei	shade67>shade56	61.243749	23.9648196	5.8925615	4.0669613	4.76E-05	0.00538972
MA_190687g0010	AT1G28570		SGNH hydrolase-type esterase superfamily pro	shade67>shade56	221.37196	19.19121621	4.3179928	4.4444762	8.81E-06	0.00140178
MA_10265740g0010	AT1G34300		lectin protein kinase family protein;(source:Ara	shade67>shade56	13.049835	6.827765434	1.6550327	4.1254566	3.70E-05	0.00443591
MA_9827161g0010	AT1G50510		indigoidine synthase A family protein;(source:A	shade67>shade56	350.65976	24.79895514	5.8899002	4.2104203	2.55E-05	0.00332946
MA_27434g0010	AT1G55790		ferredoxin-fold anticodon-binding domain prot	shade67>shade56	35.061281	22.4663389	5.5747433	4.0300222	5.58E-05	0.00613067
MA_119176g0010	AT1G56145		Leucine-rich repeat transmembrane protein ki	shade67>shade56	212.29721	11.61665618	2.5818513	4.4993514	6.82E-06	0.00114018
MA_242575g0010	AT1G56720		Protein kinase superfamily protein;(source:Ara	shade67>shade56	144.14445	23.65612586	5.7870066	4.0878	4.35E-05	0.00502182
MA_650284g0010	AT1G59740	NRT1/ PTR FAMILY 4.3 (NPF4.3)	Major facilitator superfamily protein;(source:A	shade67>shade56	117.29519	24.4485448	4.7248031	5.1745109	2.29E-07	0.00010074
MA_1734g0010	AT1G65450	GLAUCE (GLC)	Contains dual transcription units and alternati	shade67>shade56	67.713142	2.31918862	0.5679846	4.0831891	4.44E-05	0.00509479
MA_52897g0010	AT1G66920		Protein kinase superfamily protein;(source:Ara	shade67>shade56	214.04324	22.16541059	4.3348556	5.1132985	3.17E-07	0.0001264
MA_9239567g0010	AT1G69930	GLUTATHIONE S-TRANSFERASE	Encodes glutathione transferase belonging to t	shade67>shade56	48.887639	6.951129764	1.4925065	4.6573532	3.20E-06	0.00058802
MA_129851g0010	AT1G72470	EXOCYST SUBUNIT EXO70 FAMI	A member of EXO70 gene family, putative exo	shade67>shade56	68.886724	23.1219094	5.8916095	3.9245489	8.69E-05	0.00824038
MA_553396g0010	AT1G74310	HEAT SHOCK PROTEIN 101 (HSP	Encodes ClpB1, which belongs to the Casein ly	shade67>shade56	229.3189	24.41459867	4.582808	5.3274321	9.96E-08	5.34E-05
MA_88684g0010	AT1G74460		GDSL-motif esterase/acyltransferase/lipase. E	shade67>shade56	198.59424	0.780591597	0.1767749	4.4157374	1.01E-05	0.00158375
MA_180495g0010	AT1G78060		Glycosyl hydrolase family protein;(source:Arap	shade67>shade56	104.55114	1.492501596	0.3435133	4.3448148	1.39E-05	0.00205549
MA_108157g0010	AT1G78800		UDP-Glycosyltransferase superfamily protein;(	shade67>shade56	288.92213	24.91890254	4.5419299	5.4864128	4.10E-08	2.44E-05
MA_10348903g0010	AT1G78895		Reticulon family protein;(source:Araport11)	shade67>shade56	95.841286	17.97643928	3.2894734	5.464838	4.63E-08	2.72E-05
MA_10429874g0010	AT1G80160	GLYOXYLASE I 7 (GLYI7)	Vicinal oxygen chelate (VOC) superfamily mem	shade67>shade56	161.27715	23.12495978	5.8609333	3.9456105	7.96E-05	0.00785557
MA_395055g0010	AT1G80820	CINNAMOYL COA REDUCTASE (	Encodes an cinnamoyl CoA reductase isoform.	shade67>shade56	40.899254	30	6.0094577	4.992131	5.97E-07	0.00015401
MA_19208g0030	AT2G01850	ENDOXYLOGLUCAN TRANSFERA	EXGT-A3 has homology to xyloglucan endotran	shade67>shade56	124.3984	22.68203697	4.60999	4.9201922	8.65E-07	0.00020673
MA_16619g0010	AT2G02450	LONG VEGETATIVE PHASE 1 (LO	NAC domain containing protein 35;(source:Ara	shade67>shade56	76.088784	22.14299267	4.3836582	5.0512589	4.39E-07	0.00015401
MA_78676g0020	AT2G13600	SLOW GROWTH 2 (SLO2)	Encodes a pentatricopeptide repeat protein be	shade67>shade56	196.9592	21.58842311	4.6065824	4.6864294	2.78E-06	0.0005233
MA_101849g0010	AT2G17040	NAC DOMAIN CONTAINING PRO	Member of the NAC transcription factor family	shade67>shade56	461.71542	26.84616555	5.2685729	5.0955289	3.48E-07	0.00013233
MA_7493784g0010	AT2G21730	CINNAMYL ALCOHOL DEHYDRO	cinnamyl alcohol dehydrogenase homolog 2;(s	shade67>shade56	143.36126	22.94004279	4.4972198	5.1009388	3.38E-07	0.00013122
MA_455g0010	AT2G22250	ASPARTATE AMINOTRANSFERAS	Encodes a prokaryotic-type plastidic aspartate	shade67>shade56	154.31616	21.73531843	4.5262512	4.8020575	1.57E-06	0.00033066
MA_7330598g0010	AT2G22590		UDP-Glycosyltransferase superfamily protein;(	shade67>shade56	341.19687	20.19612958	4.4945833	4.4934376	7.01E-06	0.00116312
MA_177197g0020	AT2G24130		Leucine-rich receptor-like protein kinase family	shade67>shade56	107.67522	26.02330665	5.8956843	4.4139586	1.01E-05	0.00158504
MA_10427649g0020	AT2G26700	PINOID2 (PID2)	Member of AGC VIIIa Kinase gene family. Enco	shade67>shade56	145.97226	3.016886255	0.6021253	5.0103961	5.43E-07	0.00015401
MA_20782g0010	AT2G27610		Tetratricopeptide repeat (TPR)-like superfamily	shade67>shade56	185.7094	1.647532558	0.361988	4.5513464	5.33E-06	0.00093994
MA_906876g0010	AT2G29380	HIGHLY ABA-INDUCED PP2C GE	highly ABA-induced PP2C protein 3;(source:Ara	shade67>shade56	101.08365	2.557108004	0.6497897	3.9352856	8.31E-05	0.0080187
MA_116552g0010	AT2G32010	CVP2 LIKE 1 (CVL1)	Encodes an inositol polyphosphate 5?-phosph	shade67>shade56	203.68622	1.477916508	0.3769251	3.9209823	8.82E-05	0.00831233
MA_161036g0010	AT2G33150	PEROXISOMAL 3-KETOACYL-CO	Encodes an organellar (peroxisome, glyoxysom	shade67>shade56	62.413835	22.23258589	5.6952758	3.9036891	9.47E-05	0.008787
MA_10432854g0010	AT2G34930		disease resistance family protein / LRR family p	shade67>shade56	246.28107	19.21591367	4.4020052	4.3652638	1.27E-05	0.00191227
MA_10434829g0020	AT2G35730		Heavy metal transport/detoxification superfam	shade67>shade56	257.51116	1.652742878	0.419015	3.9443524	8.00E-05	0.00785557
MA_26811g0010	AT2G36750	UDP-GLUCOSYL TRANSFERASE 7	UDP-glucosyl transferase 73C1;(source:Araport	shade67>shade56	642.45689	20.92464441	3.062855	6.831745	8.39E-12	2.09E-08
MA_8644998g0010	AT2G36830	GAMMA TONOPLAST INTRINSIC	Encodes a tonoplast intrinsic protein, which fu	shade67>shade56	57.502335	22.85053915	5.8929248	3.8776228	0.00010548	0.00957963
MA_15920g0010	AT2G38300		myb-like HTH transcriptional regulator family p	shade67>shade56	61.559852	20.26489734	3.19972	6.3333346	2.40E-10	2.99E-07
MA_41286g0020	AT2G39210		Major facilitator superfamily protein;(source:A	shade67>shade56	265.38725	9.774556004	2.4734683	3.9517612	7.76E-05	0.00778006
MA_10429137g0020	AT2G44460	BETA GLUCOSIDASE 28 (BGLU28	beta glucosidase 28;(source:Araport11)	shade67>shade56	610.9023	11.28373972	2.8598257	3.945604	7.96E-05	0.00785557
MA_9544285g0010	AT2G45660	AGAMOUS-LIKE 20 (AGL20)	Controls flowering and is required for CO to pr	shade67>shade56	59.14217	24.48104131	5.4303222	4.5082115	6.54E-06	0.00110231
MA_9957507g0010	AT2G45670	LYSOPHOSPHATIDYLETHANOLA	Encodes an acyl-CoA: lysophosphatidylethanol	shade67>shade56	77.389898	1.950904293	0.3984553	4.8961686	9.77E-07	0.000226
MA_10431662g0010	AT3G01160		pre-rRNA-processing ESF1-like protein;(source:	shade67>shade56	39.915146	21.71071847	5.5445025	3.9157199	9.01E-05	0.00843675
MA_187159g0010	AT3G01190		Peroxidase superfamily protein;(source:Arapor	shade67>shade56	72.076916	23.3149957	5.5560126	4.196354	2.71E-05	0.00349982
MA_9076332g0010	AT3G04140		Ankyrin repeat family protein;(source:Araport1	shade67>shade56	16.847116	3.1738832	0.8031452	3.9518174	7.76E-05	0.00778006
MA_8035959g0010	AT3G04720	PATHOGENESIS-RELATED 4 (PR4	Encodes a protein similar to the antifungal chit	shade67>shade56	137.62157	25.66154289	5.4483365	4.7099776	2.48E-06	0.00048095
MA_101858g0010	AT3G11980	MALE STERILITY 2 (MS2)	Similar to fatty acid reductases.	shade67>shade56	21.755398	30	6.0094222	4.9921605	5.97E-07	0.00015401
MA_9825276g0010	AT3G12390		Nascent polypeptide-associated complex (NAC	shade67>shade56	64.092653	25.42688374	5.896436	4.3122462	1.62E-05	0.00230278
MA_85286g0010	AT3G13080	ATP-BINDING CASSETTE C3 (AB	encodes an ATP-dependent MRP-like ABC tran	shade67>shade56	68.252939	25.17279858	5.8945496	4.2705211	1.95E-05	0.00267964
MA_10432070g0010	AT3G17611	RHOMBOID-LIKE PROTEIN 14 (R	RHOMBOID-like protein 14;(source:Araport11)	shade67>shade56	308.39347	9.553088024	1.4137652	6.7571957	1.41E-11	3.31E-08
MA_10208460g0010	AT3G18090	NUCLEAR RNA POLYMERASE D2	Encodes a subunit of RNA polymerase IV (aka	shade67>shade56	358.56828	25.8970636	5.8899104	4.3968519	1.10E-05	0.00170264
MA_9208522g0010	AT3G19640	MAGNESIUM TRANSPORTER 4 (	Transmembrane magnesium transporter. One	shade67>shade56	73.279952	30	5.5599977	5.3956857	6.83E-08	3.85E-05
MA_10342550g0010	AT3G21000		Gag-Pol-related retrotransposon family protein	shade67>shade56	43.666787	30	6.0073801	4.9938575	5.92E-07	0.00015401
MA_226036g0010	AT3G27970		Exonuclease family protein;(source:Araport11)	shade67>shade56	173.81697	10.14610409	1.5841105	6.4049221	1.50E-10	2.20E-07
MA_9736190g0010	AT3G43660		The gene encodes a putative nodulin-like21 pr	shade67>shade56	72.609617	7.897492333	1.4719195	5.3654377	8.08E-08	4.50E-05
MA_218285g0010	AT3G44050		P-loop containing nucleoside triphosphate hyd	shade67>shade56	82.801515	23.49464132	5.6822669	4.1347303	3.55E-05	0.00429705
MA_301190g0010	AT3G47570		Leucine-rich repeat protein kinase family prote	shade67>shade56	247.21192	23.80674082	5.8189401	4.0912504	4.29E-05	0.00497479
MA_551352g0010	AT3G50740	UDP-GLUCOSYL TRANSFERASE 7	UGT72E1 is an UDPG:coniferyl alcohol glucosyl	shade67>shade56	44.297926	9.692525974	1.8236437	5.3149229	1.07E-07	5.51E-05
MA_9132531g0010	AT3G51520	DIACYLGLYCEROL ACYLTRANSFE	Encodes a functional acyl-CoA:diacylglycerol a	shade67>shade56	100.09837	1.27190695	0.3212249	3.9595523	7.51E-05	0.00761091
MA_10430424g0010	AT3G54420	HOMOLOG OF CARROT EP3-3 C	encodes an EP3 chitinase that is expressed dur	shade67>shade56	69.439419	1.904793455	0.4851331	3.9263317	8.63E-05	0.00822125
MA_96570g0010	AT3G58850	PHY RAPIDLY REGULATED 2 (PA	Encodes PHYTOCHROME RAPIDLY REGULATED	shade67>shade56	151.15644	25.46565052	4.6671481	5.4563622	4.86E-08	2.82E-05
MA_227006g0010	AT3G59630		diphthamide synthesis DPH2 family protein;(sc	shade67>shade56	285.32735	23.04775626	3.6101531	6.3841493	1.72E-10	2.43E-07
MA_10435788g0030	AT3G60850		hypothetical protein;(source:Araport11)	shade67>shade56	44.862537	22.27784827	4.2893835	5.1937179	2.06E-07	9.18E-05
MA_129478g0010	AT3G61460	BRASSINOSTEROID-RESPONSIVE	Encodes a novel ring finger protein and forms	shade67>shade56	269.27653	25.02047576	4.7458892	5.2720312	1.35E-07	6.64E-05
MA_14234g0010	AT3G62550		Adenine nucleotide alpha hydrolases-like supe	shade67>shade56	91.451935	2.124069347	0.5318995	3.9933657	6.51E-05	0.00684088
MA_90455g0010	AT4G00290	(MSL1)	Encodes a non-selective mechanosensitive ion	shade67>shade56	78.092407	23.01422025	4.5550231	5.0524925	4.36E-07	0.00015401
MA_62903g0010	AT4G00880	SMALL AUXIN UPREGULATED R	SAUR-like auxin-responsive protein family;(sou	shade67>shade56	186.56969	30	6.0096019	4.9920112	5.98E-07	0.00015401
MA_155037g0010	AT4G01130		GDSL-motif esterase/acyltransferase/lipase. E	shade67>shade56	73.867381	30	6.0075272	4.9937352	5.92E-07	0.00015401
MA_10435309g0010	AT4G02780	GA REQUIRING 1 (GA1)	Catalyzes the conversion of geranylgeranyl pyr	shade67>shade56	1484.8314	21.66426694	5.0131962	4.3214481	1.55E-05	0.00223228
MA_10427046g0010	AT4G13430	ISOPROPYL MALATE ISOMERAS	Encodes a methylthioalkylmalate isomerase in	shade67>shade56	98.540445	1.006114198	0.2302775	4.3691388	1.25E-05	0.00188536

MA_4231108g0010	AT4G16970		Protein kinase superfamily protein;(source:Ara	shade67>shade56	132.69181	23.24712591	5.8316618	3.9863638	6.71E-05	0.00702839
MA_10426824g0010	AT4G17785	MYB DOMAIN PROTEIN 39 (MYB	Encodes a putative transcription factor (MYB39	shade67>shade56	74.263519	2.386550787	0.5503552	4.3363827	1.45E-05	0.0021182
MA_10433704g0010	AT4G18550	DAD1-LIKE SEEDING ESTABLISHM	DSEL is cytosolic acylhydrolase that shows pref	shade67>shade56	190.61376	2.085062526	0.3396734	6.1384327	8.33E-10	8.82E-07
MA_10428179g0010	AT4G19450		Major facilitator superfamily protein;(source:A	shade67>shade56	415.5138	2.267466224	0.5375758	4.2179469	2.47E-05	0.00323386
MA_12346g0010	AT4G23290	CYSTEINE-RICH RLK (RECEPTOR-	Encodes a cysteine-rich receptor-like protein ki	shade67>shade56	22.587358	21.92887916	3.1855846	6.883785	5.83E-12	1.64E-08
MA_178762g0010	AT4G23440		Disease resistance protein (TIR-NBS class);(sou	shade67>shade56	94.75764	30	5.8085164	5.16483	2.41E-07	0.000105
MA_498754g0010	AT4G24130		DUF538 family protein (Protein of unknown fu	shade67>shade56	45.012723	2.359532685	0.5523136	4.2720886	1.94E-05	0.00266954
MA_475302g0010	AT4G26090	RESISTANT TO P. SYRINGAE 2 (R	Encodes a plasma membrane protein with leu	shade67>shade56	69.501895	18.84260247	3.8365974	4.9112795	9.05E-07	0.00021201
MA_5382714g0010	AT4G26330	UNFERTILIZED EMBRYO SAC 17	Subtilisin-like serine endopeptidase family prot	shade67>shade56	217.52282	22.4460546	3.6951903	6.0743975	1.24E-09	1.20E-06
MA_9399920g0010	AT4G26740	SEED GENE 1 (ATS1)	Encodes caleosin, a 27-kDa protein found with	shade67>shade56	122.09961	9.820074023	2.017926	4.8664193	1.14E-06	0.00025446
MA_551848g0020	AT4G26970	ACONITASE 2 (ACO2)	Encodes an aconitase that can catalyze the con	shade67>shade56	60.353095	24.89660816	5.8941381	4.2239608	2.40E-05	0.00318463
MA_7354451g0010	AT4G28250	EXPANSIN B3 (EXPB3)	putative beta-expansin/allergen protein. Nami	shade67>shade56	143.08438	1.612388608	0.3690315	4.3692442	1.25E-05	0.00188536
MA_9930140g0010	AT4G30170		Peroxidase family protein;(source:Araport11)	shade67>shade56	62.037677	21.58697676	4.2647119	5.0617667	4.15E-07	0.00015287
MA_36985g0020	AT4G33300	ADR1-LIKE 1 (ADR1-L1)	Encodes a member of the ADR1 family nucleot	shade67>shade56	45.027778	8.91149957	2.2191578	4.0157124	5.93E-05	0.00639851
MA_501572g0010	AT4G33720	(ATCAPE3)	CAP (Cysteine-rich secretory proteins, Antigen	shade67>shade56	178.30396	19.21367128	4.4740714	4.294449	1.75E-05	0.00244747
MA_15237g0010	AT4G34100	ECERIFERUM 9 (CER9)	Encodes a protein involved in cuticular wax bio	shade67>shade56	34.446299	20.08009195	4.3147584	4.6538161	3.26E-06	0.0005944
MA_10427611g0010	AT4G38190	CELLULOSE SYNTHASE LIKE D4 (	encodes a gene similar to cellulose synthase	shade67>shade56	70.581856	9.382576657	1.4742493	6.3643081	1.96E-10	2.62E-07
MA_27312g0010	AT5G02900	CYTOCHROME P450, FAMILY 96	member of CYP96A	shade67>shade56	41.060459	24.59198292	5.1543598	4.7711033	1.83E-06	0.00037641
MA_10425930g0010	AT5G04895	ABA OVERLY SENSITIVE 6 (ABO6	DEA(D/H)-box RNA helicase family protein;(sou	shade67>shade56	167.21642	30	6.0068133	4.9943287	5.90E-07	0.00015401
MA_10238446g0010	AT5G05170	CONSTITUTIVE EXPRESSION OF	Encodes a cellulose synthase isomer. CESA3 mu	shade67>shade56	166.18441	24.65722666	5.8904557	4.1859625	2.84E-05	0.00363066
MA_9328561g0010	AT5G05850	PLANT INTRACELLULAR RAS GRO	Encodes PIRL1, a member of the Plant Intracell	shade67>shade56	56.744221	30	6.0076142	4.9936629	5.92E-07	0.00015401
MA_422924g0010	AT5G06360		Ribosomal protein S8e family protein;(source:A	shade67>shade56	99.352722	19.00542484	4.5153843	4.2090382	2.56E-05	0.00333958
MA_9786598g0010	AT5G07580	(ERF106)	encodes a member of the ERF (ethylene respon	shade67>shade56	57.692637	21.18347183	4.4742683	4.7345109	2.20E-06	0.00043835
MA_6332848g0010	AT5G08570		Pyruvate kinase family protein;(source:Araport	shade67>shade56	61.577376	7.314099244	1.5687699	4.6623147	3.13E-06	0.00057784
MA_10432569g0010	AT5G09970	CYTOCHROME P450, FAMILY 78	member of CYP78A	shade67>shade56	399.65958	10.03243663	1.4394621	6.9695734	3.18E-12	9.61E-09
MA_8066361g0010	AT5G17540		HXXXD-type acyl-transferase family protein;(so	shade67>shade56	70.935852	23.08690862	5.8129366	3.971643	7.14E-05	0.00729666
MA_10192245g0010	AT5G17920	METHIONINE SYNTHESIS 1 (ATM	Encodes a cytosolic cobalamin-independent m	shade67>shade56	120.13048	23.46768982	5.8905826	3.9839336	6.78E-05	0.00708316
MA_10428851g0010	AT5G18980		ARM repeat superfamily protein;(source:Arapo	shade67>shade56	300.34056	19.39280001	3.3221509	5.8374231	5.30E-09	3.94E-06
MA_960556g0010	AT5G19930	PLASMA MEMBRANE GLUCOSE-	PGR is putative plasma membrane glucose- res	shade67>shade56	111.16564	24.59536423	4.3200391	5.6933199	1.25E-08	8.24E-06
MA_121630g0010	AT5G20480	EF-TU RECEPTOR (EFR)	Encodes a predicted leucine-rich repeat recept	shade67>shade56	26.649066	22.00904044	5.479014	4.016971	5.90E-05	0.00638073
MA_41345g0010	AT5G20950	(BGLC1)	Encodes a beta-glucosidase involved in xyloglu	shade67>shade56	64.774986	9.855500114	1.8217342	5.409955	6.30E-08	3.61E-05
MA_10437273g0010	AT5G24080		Protein kinase superfamily protein;(source:Ara	shade67>shade56	128.58433	25.06420688	5.8916771	4.2541718	2.10E-05	0.00281904
MA_132981g0010	AT5G24760		GroES-like zinc-binding dehydrogenase family	shade67>shade56	11339.414	1.617442195	0.4114815	3.9307775	8.47E-05	0.00810721
MA_10434521g0010	AT5G27100	GLUTAMATE RECEPTOR 2.1 (GL	member of Putative ligand-gated ion channel s	shade67>shade56	195.80201	27.11057913	5.3083363	5.1071706	3.27E-07	0.00012934
MA_510832g0010	AT5G33406		hAT dimerization domain-containing protein /	shade67>shade56	79.296602	3.724433237	0.8859067	4.2040921	2.62E-05	0.00340297
MA_8760260g0010	AT5G36930		Disease resistance protein (TIR-NBS-LRR class)	shade67>shade56	35.361848	23.57751423	5.8966228	3.9984776	6.38E-05	0.00672822
MA_494813g0010	AT5G39320	UDP-GLUCOSE DEHYDROGENAS	UDP-glucose 6-dehydrogenase family protein;(	shade67>shade56	118.97449	21.89497984	4.3769355	5.0023537	5.66E-07	0.00015401
MA_183130g0020	AT5G44030	CELLULOSE SYNTHASE A4 (CESA	Encodes a cellulose synthase involved in secon	shade67>shade56	130.7649	18.39774898	4.2813283	4.2972058	1.73E-05	0.00244001
MA_10428737g0010	AT5G44380	(ATBBE28)	FAD-binding Berberine family protein;(source:	shade67>shade56	93.64181	23.02795435	4.4935545	5.1246635	2.98E-07	0.00012247
MA_986218g0010	AT5G44700	GASSHO 2 (GSO2)	Encodes GASSHO2 (GSO2), a putative leucine-r	shade67>shade56	165.23896	21.02946968	4.4369235	4.7396512	2.14E-06	0.0004294
MA_10319538g0010	AT5G45370	USUALLY MULTIPLE ACIDS MOV	nodulin MtN21-like transporter family protein	shade67>shade56	17.447841	14.92039355	3.6351066	4.1045272	4.05E-05	0.00477606
MA_90634g0010	AT5G46610		aluminum activated malate transporter family	shade67>shade56	64.971654	7.108339214	1.802015	3.9446614	7.99E-05	0.00785557
MA_83446g0020	AT5G50400	PURPLE ACID PHOSPHATASE 27	purple acid phosphatase 27;(source:Araport11	shade67>shade56	317.71611	21.33455177	3.4619624	6.1625602	7.16E-10	7.77E-07
MA_321195g0010	AT5G51780		basic helix-loop-helix (bHLH) DNA-binding supe	shade67>shade56	168.47283	12.79658274	2.427048	5.2724885	1.35E-07	6.64E-05
MA_39500g0010	AT5G53470	ACYL-COA BINDING PROTEIN 1 (	Encodes an acyl-CoA binding protein that is loc	shade67>shade56	109.56587	1.789931901	0.3968894	4.509901	6.49E-06	0.00109794
MA_766568g0010	AT5G54930	METABOLIC NETWORK MODULA	AT hook motif-containing protein;(source:Arap	shade67>shade56	81.183942	10.29709703	2.5140508	4.0958189	4.21E-05	0.00493173
MA_10426326g0020	AT5G55090	MITOGEN-ACTIVATED PROTEIN	member of MEKK subfamily	shade67>shade56	233.8183	9.34552367	1.3981422	6.6842442	2.32E-11	4.82E-08
MA_10428846g0020	AT5G55250	IAA CARBOXYLMETHYLTRANSFE	Encodes an enzyme which specifically converts	shade67>shade56	48.710255	17.97497551	3.8356595	4.6862803	2.78E-06	0.0005233
MA_10426320g0010	AT5G56960		basic helix-loop-helix (bHLH) DNA-binding fami	shade67>shade56	94.262969	23.53511908	5.4073855	4.3524027	1.35E-05	0.00199954
MA_10434319g0010	AT5G58360	OVATE FAMILY PROTEIN 3 (OFP	ovate family protein 3;(source:Araport11)	shade67>shade56	177.21754	1.223426997	0.2913975	4.1984811	2.69E-05	0.00347772
MA_5587g0010	AT5G60700		glycosyltransferase family protein 2;(source:Ar	shade67>shade56	186.46586	1.205843312	0.3092628	3.8990898	9.66E-05	0.00892206
MA_92381g0010	AT5G64140	RIBOSOMAL PROTEIN S28 (RPS2	Encodes a putative ribosomal protein S28.	shade67>shade56	121.24359	23.74769686	5.7573079	4.1247919	3.71E-05	0.00443618
MA_261256g0010	AT5G64570	BETA-D-XYLOSIDASE 4 (XYL4)	Encodes a beta-d-xylosidase that belongs to fa	shade67>shade56	41.297439	22.53010886	5.4595113	4.1267629	3.68E-05	0.00442333

Table S2 Genes for which the levels of expression were significantly higher in the 56°N population as compared to the 67°N population in response to SHADE in Norway spruce

Gene_ID	TAIR	Primary Gene Symbol	Gene Model Description	Expression	baseMean	log2FoldChange	lfcSE	stat	pvalue	padj
MA_10325260g0010	AT1G04580	ALDEHYDE OXIDASE 4 (AO4)	Encodes aldehyde oxidase AAO4 preferentially	shade67<shade56	81.315393	-22.88013074	5.8918542	-3.88335	0.00010303	0.00941731
MA_536645g0010	AT1G05200	GLUTAMATE RECEPTOR 3.4 (GL	Encodes a putative glutamate receptor GLR3 w	shade67<shade56	272.51679	-25.55209379	4.2803869	-5.969576	2.38E-09	1.91E-06
MA_355745g0010	AT1G05270		TraB family protein;(source:Araport11)	shade67<shade56	214.99011	-12.54145848	2.4921623	-5.03236	4.84E-07	0.00015401
MA_213682g0010	AT1G06640		encodes a protein whose sequence is similar to	shade67<shade56	210.29368	-23.77830423	5.8901633	-4.036952	5.42E-05	0.00599576
MA_10427341g0010	AT1G07650		Leucine-rich repeat transmembrane protein kin	shade67<shade56	2798.077	-3.098507079	0.7826282	-3.959105	7.52E-05	0.00761091
MA_496233g0010	AT1G11180	SECRETORY CARRIER MEMBRAN	Secretory carrier membrane protein (SCAMP) f	shade67<shade56	464.85582	-4.803988131	0.7783166	-6.17228	6.73E-10	7.50E-07
MA_10435231g0010	AT1G12860	SCREAM 2 (SCRM2)	Encodes ICE2 (Inducer of CBF Expression 2), a t	shade67<shade56	1036.0692	-2.995103713	0.7645797	-3.917321	8.95E-05	0.00840214
MA_447489g0010	AT1G15100	RING-H2 FINGER A2A (RHA2A)	Encodes a putative RING-H2 finger protein RHA	shade67<shade56	2475.4907	-2.089481029	0.5191656	-4.024691	5.71E-05	0.00619083
MA_1026335g0020	AT1G15780	NON-RECOGNITION-OF-BTH 4 (N	mediator of RNA polymerase II transcription su	shade67<shade56	34.011685	-23.05216854	5.5165931	-4.178697	2.93E-05	0.00369282
MA_10431686g0010	AT1G18090		5-3 exonuclease family protein;(source:Arapor	shade67<shade56	133.77856	-1.707936457	0.3775515	-4.523718	6.08E-06	0.00104111
MA_3340g0010	AT1G20510	OPC-8:0 COA LIGASE1 (OPCL1)	OPC-8:0 CoA ligase1;(source:Araport11)	shade67<shade56	73.538753	-30	6.0072541	-4.993962	5.92E-07	0.00015401
MA_7115g0010	AT1G22640	MYB DOMAIN PROTEIN 3 (MYB3	MYB-type transcription factor (MYB3) that rep	shade67<shade56	172.44193	-23.14887515	5.8902415	-3.930038	8.49E-05	0.00811381
MA_939384g0010	AT1G26500		Pentatricopeptide repeat (PPR) superfamily pro	shade67<shade56	76.036931	-23.46362023	5.8912287	-3.982806	6.81E-05	0.00709934
MA_15946g0010	AT1G30220	INOSITOL TRANSPORTER 2 (INT2	Inositol transporter presenting conserved extra	shade67<shade56	338.30772	-19.37093044	4.3878454	-4.414679	1.01E-05	0.00158504
MA_10433153g0020	AT1G30290		unknown protein	shade67<shade56	262.51475	-27.08410624	4.3695575	-6.198364	5.71E-10	6.71E-07
MA_9974774g0010	AT1G32300	L -GULONO-1,4-LACTONE (L -GU	D-arabinono-1,4-lactone oxidase family protein	shade67<shade56	59.887848	-20.72426353	4.3067491	-4.812043	1.49E-06	0.00031613
MA_10432360g0020	AT1G37130	NITRATE REDUCTASE 2 (NIA2)	Identified as a mutant resistant to chlorate. En	shade67<shade56	1268.3351	-1.297015908	0.25408	-5.104754	3.31E-07	0.00012979
MA_19887g0010	AT1G48500	JASMONATE-ZIM-DOMAIN PROT	jasmonate-zim-domain protein 4;(source:Arap	shade67<shade56	22.796923	-30	6.0105393	-4.991233	6.00E-07	0.00015401
MA_86183g0010	AT1G54200	BIG GRAIN 3 (BG3)	DNA mismatch repair Msh6-like protein;(sourc	shade67<shade56	296.12841	-3.275875616	0.7906427	-4.143307	3.42E-05	0.00416318
MA_564894g0010	AT1G55910	ZINC TRANSPORTER 11 PRECUR	member of Putative zinc transporter ZIP2 - like	shade67<shade56	182.443	-8.543741989	1.4440798	-5.916392	3.29E-09	2.58E-06
MA_464469g0010	AT1G56120		Leucine-rich repeat transmembrane protein kin	shade67<shade56	43.755794	-21.51556729	4.4004257	-4.889429	1.01E-06	0.00023135
MA_401306g0010	AT1G61065		1,3-beta-glucan synthase component (DUF121	shade67<shade56	232.08101	-24.50516034	5.8901333	-4.160375	3.18E-05	0.00389277
MA_16626g0010	AT1G61250	SECRETORY CARRIER 3 (SC3)	Encodes a putative secretory carrier membrane	shade67<shade56	444.83114	-2.131529526	0.5017083	-4.248544	2.15E-05	0.00288164
MA_167711g0010	AT1G64680		beta-carotene isomerase D27;(source:Araport1	shade67<shade56	3312.6325	-1.089032164	0.2432031	-4.477872	7.54E-06	0.00122246
MA_38922g0010	AT1G71050	HEAVY METAL ASSOCIATED ISO	Heavy metal transport/detoxification superfam	shade67<shade56	323.46382	-9.342012204	2.2831081	-4.091796	4.28E-05	0.00497479
MA_10180304g0010	AT1G72840		Disease resistance protein (TIR-NBS-LRR class);	shade67<shade56	165.28471	-24.62843545	5.6158792	-4.385499	1.16E-05	0.00178087
MA_10370672g0010	AT1G77460	CELLULOSE SYNTHASE INTERACT	Encodes a plasma membrane, microtubule ass	shade67<shade56	31.994045	-4.57047869	1.0090288	-4.529582	5.91E-06	0.00101675
MA_441084g0010	AT1G77840		Translation initiation factor IF2/IF5;(source:Ara	shade67<shade56	367.29826	-24.06317044	5.8899652	-4.085452	4.40E-05	0.00505909
MA_14341g0010	AT1G78020	FCS LIKE ZINC FINGER 6 (FLZ6)	FCS like zinc finger 6 is induced during energy s	shade67<shade56	412.05258	-11.32909104	1.5438017	-7.338437	2.16E-13	1.83E-09
MA_587505g0010	AT1G79620	VASCULAR-RELATED RLK 1 (VRL	VRLK1 is a LRR kinase involved in switching bet	shade67<shade56	16.397498	-8.007723581	1.7295361	-4.629984	3.66E-06	0.00065858
MA_8433628g0010	AT2G01610		Plant invertase/pectin methylesterase inhibito	shade67<shade56	47.351166	-22.33584837	5.3966668	-4.138823	3.49E-05	0.00423322
MA_8300751g0010	AT2G04570		GDSL-motif esterase/acyltransferase/lipase. En	shade67<shade56	61.237582	-18.88374371	3.705427	-5.09624	3.46E-07	0.00013233
MA_10429950g0020	AT2G13540	ABA HYPERSENSITIVE 1 (ABH1)	Encodes a nuclear cap-binding protein that for	shade67<shade56	357.93022	-3.055303903	0.7643503	-3.997256	6.41E-05	0.0067462
MA_349646g0010	AT2G14080		Disease resistance protein (TIR-NBS-LRR class)	shade67<shade56	101.04164	-11.15758155	1.5849578	-7.039671	1.93E-12	6.54E-09
MA_17793g0010	AT2G16850	PLASMA MEMBRANE INTRINSIC	plasma membrane intrinsic protein 2;(source:A	shade67<shade56	62.714376	-24.34183127	5.8938296	-4.130053	3.63E-05	0.00437292
MA_8866128g0010	AT2G17570	CIS-PRENYLTRANSFERASE 1 (CPT	Undecaprenyl pyrophosphate synthetase famil	shade67<shade56	486.50293	-3.685883792	0.8846408	-4.166531	3.09E-05	0.00383343
MA_5951905g0010	AT2G18050	HISTONE H1-3 (HIS1-3)	encodes a structurally divergent linker histone	shade67<shade56	236.99984	-26.56529095	5.8901749	-4.510102	6.48E-06	0.00109794
MA_346860g0010	AT2G19130		S-locus lectin protein kinase family protein;(so	shade67<shade56	207.98178	-2.253851841	0.3374051	-6.679957	2.39E-11	4.82E-08
MA_63882g0010	AT2G19790	ADAPTOR PROTEIN COMPLEX 4S	Encodes a component of the AP4 complex and	shade67<shade56	224.03066	-21.39560558	4.2691118	-5.011723	5.39E-07	0.00015401
MA_266310g0010	AT2G23910		NAD(P)-binding Rossmann-fold superfamily pro	shade67<shade56	449.84823	-2.093146683	0.4448531	-4.705253	2.54E-06	0.00048775
MA_10431396g0010	AT2G26490	JINGUBANG (JGB)	JGB contains seven WD40 repeats and is highly	shade67<shade56	17.775076	-24.01944223	5.9027062	-4.069225	4.72E-05	0.00535193
MA_780853g0010	AT2G28160	FER-LIKE IRON DEFICIENCY INDU	Encodes a putative transcription factor that re	shade67<shade56	68.760449	-24.30070684	5.5800542	-4.354923	1.33E-05	0.00198363
MA_9765350g0010	AT2G28800	ALBINO 3 (ALB3)	member of Chloroplast membrane protein ALB	shade67<shade56	180.19431	-26.40437984	5.8904643	-4.482563	7.38E-06	0.00120048
MA_206815g0010	AT2G29690	ANTHRANILATE SYNTHASE 2 (AS	Encode a functional anthranilate synthase prot	shade67<shade56	140.64084	-1.878720942	0.4452341	-4.219625	2.45E-05	0.00322627
MA_279076g0010	AT2G30933		Carbohydrate-binding X8 domain superfamily p	shade67<shade56	57.035001	-19.49922833	4.3004765	-4.534202	5.78E-06	0.00100058
MA_10436215g0020	AT2G31750	UDP-GLUCOSYL TRANSFERASE 7	Encodes an auxin glycosyltransferase that is lik	shade67<shade56	305.02062	-12.53572629	2.8605809	-4.382231	1.17E-05	0.00180033
MA_564543g0010	AT2G31900	MYOSIN-LIKE PROTEIN XIF (XIF)	Encodes an novel myosin isoform.	shade67<shade56	76.765086	-10.83430494	2.767198	-3.915262	9.03E-05	0.00843675
MA_1160818g0020	AT2G32850		Protein kinase superfamily protein;(source:Ara	shade67<shade56	88.015676	-21.26675086	4.2646005	-4.98681	6.14E-07	0.00015463
MA_960018g0020	AT2G34050		ATP synthase F1 complex assembly factor;(sou	shade67<shade56	371.48094	-13.99385857	2.3307011	-6.004141	1.92E-09	1.66E-06
MA_834070g0010	AT2G35430		Zinc finger C-x8-C-x5-C-x3-H type family protein	shade67<shade56	89.347434	-21.25102673	4.3980546	-4.831915	1.35E-06	0.00028904
MA_10351640g0010	AT2G35800	S-ADENOSYL METHIONINE TRAN	Encodes a predicted calcium-dependent S-ade	shade67<shade56	41.524372	-23.62600107	4.2967167	-5.498617	3.83E-08	2.31E-05
MA_122962g0010	AT2G38120	AUXIN RESISTANT 1 (AUX1)	Encodes an auxin influx transporter. AUX1 resi	shade67<shade56	592.19336	-1.797521412	0.3642197	-4.935267	8.00E-07	0.00019484
MA_95687g0010	AT2G38940	PHOSPHATE TRANSPORTER 1;4	Encodes Pht1;4, a member of the Pht1 family o	shade67<shade56	134.32187	-25.46575205	5.893003	-4.321354	1.55E-05	0.00223228
MA_10428530g0010	AT2G39480	ATP-BINDING CASSETTE B6 (ABC	P-glycoprotein 6;(source:Araport11)	shade67<shade56	46.515492	-9.455428442	1.7703551	-5.340979	9.24E-08	5.08E-05
MA_418372g0010	AT2G40610	EXPANSIN A8 (EXPA8)	member of Alpha-Expansin Gene Family. Nami	shade67<shade56	61.370234	-23.02511844	5.8923518	-3.907628	9.32E-05	0.00866945
MA_634100g0010	AT2G42360		RING/U-box superfamily protein;(source:Arapo	shade67<shade56	174.80972	-23.15469167	5.7896012	-3.999359	6.35E-05	0.00672822
MA_3206652g0010	AT2G42850	CYTOCHROME P450, FAMILY 71	cytochrome P450, family 718;(source:Araport1	shade67<shade56	42.720765	-30	5.3576359	-5.599485	2.15E-08	1.38E-05
MA_253584g0010	AT2G45550	CYTOCHROME P450, FAMILY 76	member of CYP76C	shade67<shade56	156.33341	-3.129882756	0.7288422	-4.294321	1.75E-05	0.00244747
MA_10427075g0010	AT2G45560	CYTOCHROME P450, FAMILY 76	cytochrome P450 monooxygenase	shade67<shade56	59.97644	-2.642012325	0.4197184	-6.294726	3.08E-10	3.72E-07
MA_1925g0010	AT2G48130	(LTPG15)	Encodes a plasma membrane-localized glycosy	shade67<shade56	196.7309	-1.606474795	0.3603114	-4.458573	8.25E-06	0.00132264
MA_7555671g0010	AT3G02470	S-ADENOSYLMETHIONINE DECA	Encodes a S-adenosylmethionine decarboxylas	shade67<shade56	91.999685	-22.6108394	5.6409809	-4.008317	6.12E-05	0.00652739
MA_80461g0010	AT3G04220		Disease resistance protein (TIR-NBS-LRR class)	shade67<shade56	39.54445	-30	6.0078069	-4.993503	5.93E-07	0.00015401
MA_909045g0010	AT3G07880	SUPERCENTIPEDE1 (SCN1)	RhoGTPase GDP dissociation inhibitor (RhoGDI	shade67<shade56	96.913487	-21.61953728	4.3552138	-4.964059	6.90E-07	0.00016986
MA_10429484g0010	AT3G14075		Mono-/di-acylglycerol lipase, N-terminal;(sourc	shade67<shade56	60.455908	-23.47161132	5.8943013	-3.982085	6.83E-05	0.0071034
MA_5681263g0010	AT3G14360	OIL BODY LIPASE 1 (ATOBL1)	alpha/beta-Hydrolases superfamily protein;(so	shade67<shade56	146.95975	-21.7894401	3.6013222	-6.0504	1.44E-09	1.32E-06
MA_403867g0010	AT3G14820		GDSL-motif esterase/acyltransferase/lipase. En	shade67<shade56	239.45738	-26.41920125	5.893083	-4.483087	7.36E-06	0.00120048
MA_16493g0010	AT3G16520	UDP-GLUCOSYL TRANSFERASE 8	UDP-glucosyl transferase 88A1;(source:Araport	shade67<shade56	195.61151	-27.88664738	5.8930942	-4.732089	2.22E-06	0.00044153
MA_9959752g0010	AT3G20630	UBIQUITIN-SPECIFIC PROTEASE	Encodes a ubiquitin-specific protease. Identica	shade67<shade56	65.003028	-30	6.0070734	-4.994112	5.91E-07	0.00015401
MA_10428105g0010	AT3G22060		contains Pfam profile: PF01657 Domain of unk	shade67<shade56	46.626838	-18.84022474	3.4184677	-5.511307	3.56E-08	2.18E-05
MA_10367103g0010	AT3G22220		hAT transposon superfamily;(source:Araport11	shade67<shade56	206.49539	-23.52400821	3.6648839	-6.418759	1.37E-10	2.15E-07
MA_51552g0010	AT3G24503	ALDEHYDE DEHYDROGENASE 2C	Arabidopsis thaliana aldehyde dehydrogenase	shade67<shade56	45.01806	-22.24427383	4.4359404	-5.014557	5.32E-07	0.00015401
MA_12314g0010	AT3G28917	MINI ZINC FINGER 2 (MIF2)	mini zinc finger 2;(source:Araport11)	shade67<shade56	123.11633	-13.05871064	3.2825001	-3.978282	6.94E-05	0.0071539
MA_10426995g0010	AT3G43550		GDSL-motif esterase/acyltransferase/lipase. En	shade67<shade56	139.89151	-23.49012238	5.4596462	-4.302499	1.69E-05	0.00239039
MA_10428913g0020	AT3G44630		Disease resistance protein (TIR-NBS-LRR class)	shade67<shade56	41.328588	-3.626809213	0.895874	-4.048347	5.16E-05	0.00577497
MA_9820002g0010	AT3G45970	EXPANSIN-LIKE A1 (EXLA1)	member of EXPANSIN-LIKE. Naming convention	shade67<shade56	135.13285	-23.59508938	5.8868045	-4.008132	6.12E-05	0.00652739
MA_4364416g0010	AT3G46520	ACTIN-12 (ACT12)	Member of actin subclass composed of ACT12	shade67<shade56	67.344876	-19.21487284	3.9109052	-4.913152	8.96E-07	0.00021201
MA_9934639g0010	AT3G46960	(SKI2)	The gene encodes a DExD⁄H box RNA h	shade67<shade56	85.589589	-2.565890682	0.6452093	-3.976835	6.98E-05	0.00715652
MA_10132652g0010	AT3G47110		Leucine-rich repeat protein kinase family prote	shade67<shade56	301.15243	-19.51797408	4.3330031	-4.504491	6.65E-06	0.00111734
MA_673539g0010	AT3G48140		B12D protein;(source:Araport11)	shade67<shade56	99.159008	-21.24768603	4.3594317	-4.873958	1.09E-06	0.00024624
MA_5530991g0010	AT3G48280	CYTOCHROME P450, FAMILY 71	putative cytochrome P450	shade67<shade56	394.25791	-30	4.1804968	-7.176181	7.17E-13	3.03E-09
MA_9494886g0010	AT3G48320	CYTOCHROME P450, FAMILY 71	putative cytochrome P450	shade67<shade56	81.257292	-22.44025384	4.4949674	-4.992306	5.97E-07	0.00015401
MA_8080485g0010	AT3G49260	IQ-DOMAIN 21 (iqd21)	IQ-domain 21;(source:Araport11)	shade67<shade56	189.49709	-24.63962602	5.8903625	-4.183041	2.88E-05	0.00365557
MA_8912495g0010	AT3G49990		Low temperature viability protein;(source:Arap	shade67<shade56	69.482362	-23.5724293	5.8945255	-3.999038	6.36E-05	0.00672822
MA_499152g0010	AT3G50780		BTB/POZ domain protein;(source:Araport11)	shade67<shade56	181.36179	-17.95607005	4.3590214	-4.119289	3.80E-05	0.00453065
MA_7310594g0010	AT3G51130		transmembrane protein;(source:Araport11)	shade67<shade56	166.2113	-22.51264882	4.4382384	-5.072429	3.93E-07	0.0001471
MA_179915g0020	AT3G52500		Eukaryotic aspartyl protease family protein;(so	shade67<shade56	85.077405	-21.10809367	4.2982352	-4.910874	9.07E-07	0.00021201
MA_918643g0010	AT3G57120		Protein kinase superfamily protein;(source:Ara	shade67<shade56	38.232762	-23.74454222	5.4693731	-4.341365	1.42E-05	0.00208079
MA_10058810g0010	AT3G61680	PLASTID LIPASE1 (PLIP1)	PLIP1 encodes a plastid localized phospholipas	shade67<shade56	311.26525	-24.11401178	5.8901635	-4.093946	4.24E-05	0.00494951

MA_111976g0010	AT4G04970	GLUCAN SYNTHASE-LIKE 1 (GSL1	encodes a gene similar to callose synthase The	shade67<shade56	111.36636	-4.093241089	0.7300074	-5.607123	2.06E-08	1.34E-05
MA_169781g0010	AT4G09340		SPla/RYanodine receptor (SPRY) domain-conta	shade67<shade56	55.161341	-19.17770461	4.0645158	-4.718325	2.38E-06	0.00046377
MA_10435813g0010	AT4G10310	HIGH-AFFINITY K+ TRANSPORTE	encodes a sodium transporter (HKT1) expresse	shade67<shade56	325.95917	-22.18232556	3.1536734	-7.033806	2.01E-12	6.54E-09
MA_179643g0010	AT4G10500	DMR6-LIKE OXYGENASE 1 (DLO1	2-oxoglutarate (2OG) and Fe(II)-dependent oxy	shade67<shade56	377.3487	-12.92130221	2.8225575	-4.57787	4.70E-06	0.00083178
MA_58710g0010	AT4G10550		Subtilase family protein;(source:Araport11)	shade67<shade56	284.90435	-1.187277451	0.3004241	-3.952005	7.75E-05	0.00778006
MA_423032g0010	AT4G10780		LRR and NB-ARC domains-containing disease re	shade67<shade56	327.37919	-15.04072897	3.6782994	-4.089044	4.33E-05	0.00500861
MA_92659g0010	AT4G18390	TEOSINTE BRANCHED 1, CYCLOI	TEOSINTE BRANCHED 1, cycloidea and PCF tran	shade67<shade56	81.598303	-22.31837131	4.4340774	-5.033374	4.82E-07	0.00015401
MA_456473g0010	AT4G23140	CYSTEINE-RICH RLK (RECEPTOR-	Arabidopsis thaliana receptor-like protein kina	shade67<shade56	98.792615	-19.52952353	4.4650202	-4.373894	1.22E-05	0.00185801
MA_945672g0010	AT4G23340		2-oxoglutarate (2OG) and Fe(II)-dependent oxy	shade67<shade56	172.16112	-24.69937417	5.8902701	-4.19325	2.75E-05	0.00353428
MA_128616g0010	AT4G26050	PLANT INTRACELLULAR RAS GR(	Encodes PIRL8, a member of the Plant Intracell	shade67<shade56	96.865151	-25.06625578	5.8910747	-4.254955	2.09E-05	0.00281814
MA_17530g0010	AT4G26890	MITOGEN-ACTIVATED PROTEIN	member of MEKK subfamily	shade67<shade56	338.17932	-24.49086995	5.8898861	-4.158123	3.21E-05	0.00391345
MA_31254g0010	AT4G28200		U3 small nucleolar RNA-associated-like protein	shade67<shade56	114.00041	-20.96888821	4.4917221	-4.668341	3.04E-06	0.0005661
MA_5708g0010	AT4G32300	S-DOMAIN-2 5 (SD2-5)	S-domain-2 5;(source:Araport11)	shade67<shade56	205.36017	-12.85435167	2.6516443	-4.847691	1.25E-06	0.0002739
MA_96242g0010	AT4G34160	CYCLIN D3;1 (CYCD3;1)	encodes a cyclin D-type protein involved in the	shade67<shade56	28.327684	-30	6.0098127	-4.991836	5.98E-07	0.00015401
MA_13722g0010	AT4G37520		Peroxidase superfamily protein;(source:Arapor	shade67<shade56	129.64975	-21.47274279	4.4832093	-4.789592	1.67E-06	0.00034841
MA_10357974g0010	AT4G38070		transcription factor bHLH131-like protein;(sour	shade67<shade56	147.69388	-2.410789851	0.6004615	-4.014895	5.95E-05	0.00640439
MA_351763g0010	AT5G02230		Haloacid dehalogenase-like hydrolase (HAD) su	shade67<shade56	75.443171	-18.97876163	3.3107345	-5.732493	9.90E-09	6.76E-06
MA_19871g0020	AT5G02960		Ribosomal protein S12/S23 family protein;(sou	shade67<shade56	188.10599	-25.08343565	5.8905345	-4.258261	2.06E-05	0.00278748
MA_96757g0010	AT5G05340	PEROXIDASE 52 (PRX52)	Encodes a protein with sequence similarity to p	shade67<shade56	157.26187	-1.263494658	0.3037252	-4.159993	3.18E-05	0.00389277
MA_10280064g0010	AT5G07990	TRANSPARENT TESTA 7 (TT7)	Required for flavonoid 3' hydroxylase activity. I	shade67<shade56	591.00163	-2.52383258	0.6503736	-3.880589	0.0001042	0.0094839
MA_10308567g0040	AT5G08020	RPA70-KDA SUBUNIT B (RPA70B	Encodes a homolog of Replication Protein A. rp	shade67<shade56	235.74736	-1.878923288	0.3928419	-4.782899	1.73E-06	0.0003567
MA_93721g0020	AT5G09810	ACTIN 7 (ACT7)	Member of Actin gene family.Mutants are defe	shade67<shade56	86.773394	-30	5.4063976	-5.548981	2.87E-08	1.79E-05
MA_9106g0010	AT5G13700	POLYAMINE OXIDASE 1 (PAO1)	Encodes a protein with polyamine oxidase acti	shade67<shade56	133.92391	-24.89671027	5.8905269	-4.226568	2.37E-05	0.00315788
MA_73048g0010	AT5G15780		Pollen Ole e 1 allergen and extensin family pro	shade67<shade56	3059.7929	-2.976854627	0.682619	-4.360931	1.30E-05	0.00193675
MA_7491716g0010	AT5G17680		disease resistance protein (TIR-NBS-LRR class);	shade67<shade56	155.4035	-21.7115806	5.5174828	-3.935052	8.32E-05	0.0080187
MA_8256257g0010	AT5G20680	TRICHOME BIREFRINGENCE-LIKE	Encodes a member of the TBL (TRICHOME BIRE	shade67<shade56	223.31806	-22.76446823	4.6127576	-4.93511	8.01E-07	0.00019484
MA_95738g0010	AT5G20700		senescence-associated family protein, putative	shade67<shade56	104.26097	-4.201242853	0.89282	-4.705588	2.53E-06	0.00048775
MA_19854g0020	AT5G21960		encodes a member of the DREB subfamily A-5	shade67<shade56	36.547943	-21.52738551	5.5151606	-3.903311	9.49E-05	0.008787
MA_10434800g0010	AT5G23230	NICOTINAMIDASE 2 (NIC2)	nicotinamidase 2;(source:Araport11)	shade67<shade56	23.253605	-16.06965962	3.6305816	-4.426194	9.59E-06	0.00152023
MA_59443g0010	AT5G26220	GAMMA-GLUTAMYL CYCLOTRA	ChaC-like family protein;(source:Araport11)	shade67<shade56	522.51603	-6.937424699	0.9367568	-7.40579	1.30E-13	1.83E-09
MA_7706481g0010	AT5G45050	(WRKY16)	Encodes a member of the WRKY Transcription	shade67<shade56	78.771933	-11.19808893	2.3505736	-4.763981	1.90E-06	0.0003862
MA_10434773g0030	AT5G47500	PECTIN METHYLESTERASE 5 (PM	predicted to encode a pectin methylesterase	shade67<shade56	31.715567	-18.86120083	3.5596116	-5.298668	1.17E-07	5.95E-05
MA_9957075g0010	AT5G48930	HYDROXYCINNAMOYL-COA SHIK	At5g48930 has been shown to encode for the I	shade67<shade56	22.009697	-30	6.0103004	-4.991431	5.99E-07	0.00015401
MA_29890g0010	AT5G49890	CHLORIDE CHANNEL C (CLC-C)	member of Anion channel protein family	shade67<shade56	191.2228	-22.09201482	3.634185	-6.078946	1.21E-09	1.19E-06
MA_838949g0010	AT5G53350	CLP PROTEASE REGULATORY SU	CLP protease regulatory subunit CLPX mRNA, n	shade67<shade56	206.92373	-10.40859199	2.6518131	-3.925085	8.67E-05	0.00824038
MA_652482g0010	AT5G55490	GAMETE EXPRESSED PROTEIN 1	Encodes a transmembrane domain containing	shade67<shade56	61.204977	-24.14644217	5.8929358	-4.097523	4.18E-05	0.00490917
MA_10153873g0010	AT5G56150	UBIQUITIN-CONJUGATING ENZY	ubiquitin-conjugating enzyme 30;(source:Arap	shade67<shade56	135.84427	-20.51991807	4.4386793	-4.622978	3.78E-06	0.00067834
MA_23239g0010	AT5G58490		NAD(P)-binding Rossmann-fold superfamily pro	shade67<shade56	492.44562	-1.143730454	0.2256147	-5.069397	3.99E-07	0.00014815
MA_83574g0010	AT5G59970		Histone superfamily protein;(source:Araport11	shade67<shade56	223.28613	-25.64928987	3.5723895	-7.179869	6.98E-13	3.03E-09
MA_3003293g0010	AT5G60390	(EF1ALPHA)	GTP binding Elongation factor Tu family protei	shade67<shade56	52.962014	-22.97246948	5.8933811	-3.898012	9.70E-05	0.00894233
MA_183219g0010	AT5G63960	GIGANTEA SUPPRESSOR5 (GIS5)	Encodes the catalytic subunits of DNA polymer	shade67<shade56	72.570338	-23.25206085	5.7098909	-4.072243	4.66E-05	0.00529725
MA_10437110g0010	AT5G64790		O-Glycosyl hydrolases family 17 protein;(sourc	shade67<shade56	11.086688	-30	6.0115883	-4.990362	6.03E-07	0.00015401
MA_205355g0010	AT5G64950	MITOCHONDRIAL TRANSCRIPTI(	mTERF family protein which functions in the re	shade67<shade56	69.227533	-24.0330073	5.8934183	-4.07794	4.54E-05	0.00519707
MA_10238024g0010	AT5G66580		hypothetical protein;(source:Araport11)	shade67<shade56	13.514632	-30	6.0106723	-4.991122	6.00E-07	0.00015401
MA_904305g0010	AT4G02750		Tetratricopeptide repeat (TPR)-like superfamily	shade67<shade56	99.713923	-1.960704561	0.4126041	-4.752024	2.01E-06	0.00040586

Table S3 Allele and genotype frequencies of missense mutations in the nine candidate DEGs in response to SHADE in Norway spruce that show cline

**Gene name**	**SNP**	**Gene_ID**	**Reference allele frequency p-value**	**Alternate allele frequency p-value**	**Genotype frequency p-value**
MYB3	T132R	MA_7115g0010	**C: 2.98E-40**	**G: 1.64E-06**	**4.77E-52**
LOV1	R156K	MA_16619g0010	G: 0.424181	A: 0.139508	**8.94E-07**
LOV1	D190N	MA_16619g0010	**G: 0.036124**	**A: 0.016444**	**8.78E-11**
SCRM2	T208M	MA_10435231g0010	**C: 7.00E-11**	**T: 0.017001**	**3.59E-29**
SCRM2	S238N	MA_10435231g0010	**G: 2.38E-20**	**A: 0.014526**	**2.64E-39**
TCP2	F4S	MA_92659g0010	**T: 0.000215**	**C: 4.47E-27**	**9.13E-38**
TCP2	V25A	MA_92659g0010	**T: 6.18E-07**	**C: 7.93E-11**	**1.87E-12**
TCP2	E32G	MA_92659g0010	**A: 5.11E-07**	**G: 5.81E-11**	**1.31E-12**
TCP2	E51K	MA_92659g0010	**G: 3.03E-23**	**A: 0.001302**	**6.37E-36**
TCP2	I53F	MA_92659g0010	**A: 0.001336**	**T: 1.93E-22**	**5.44E-34**
NAC036	D206E	MA_101849g0010	**T: 8.36E-05**	**A: 3.48E-09**	**1.13E-09**
NAC036	T237M	MA_101849g0010	C: 0.758596	T: 0.090354	**0.008645**
EXPB3	K3R	MA_7354451g0010	**A: 1.14E-06**	G: 0.358795	**4.10E-09**
EXPB3	R6S	MA_7354451g0010	**G: 4.67E-11**	T: 0.317117	**4.71E-16**
EXPB3	C32R	MA_7354451g0010	**T: 3.02E-09**	**C: 3.02E-09**	**3.02E-09**
FLZ6	L88V	MA_14341g0010	**C: 7.35E-06**	G: 0.434854	**8.07E-10**
VRLK1	A265V	MA_587505g0010	C: 0.160674	**T: 0.005841**	**0.001393**
VRLK1	V283L	MA_587505g0010	**G: 1.85E-07**	**C: 0.020671**	**4.64E-08**
RPS2	S123N	MA_475302g0010	**G: 1.24E-27**	A: 0.35738	**1.36E-37**
RPS2	E166A	MA_475302g0010	**A: 1.43E-22**	C: 0.590028	**2.69E-29**

Significant p-values (p-value<0.05) are in bold

Table S4 Tukey's post-hoc test for genotype frequencies of missense mutations in the nine candidate DEGs in response to SHADE in Norway spruce that show cline

Gene name	SNP	Gene_ID	Tukey's p-value S1 vs S2	Bonferroni p-value S1 vs S2	Tukey's p-value S1 vs S3	Bonferroni p-value S1 vs S3	Tukey's p-value S1 vs S4	Bonferroni p-value S1 vs S4	Tukey's p-value S1 vs S5	Bonferroni p-value S1 vs S5	Tukey's p-value S1 vs S6	Bonferroni p-value S1 vs S6	Tukey's p-value S2 vs S3	Bonferroni p-value S2 vs S3
MYB3_T132R	T132R	MA_7115g0010	0.9	4.438543	0.3809	0.7933012	0.00101	8.41E-07	0.00101	0.00E+00	0.00101	0.00E+00	0.89999	5.5308979
LOV1_R156K	R156K	MA_16619g0010	0.7743	3.0534676	0.89999	9.9629893	0.89999	8.96E+00	0.53796	1.39E+00	0.00101	1.58E-06	0.89999	6.6357244
LOV1_D190N	D190N	MA_16619g0010	0.6904	2.3489015	0.89999	10.477417	0.21985	3.78E-01	0.00101	4.49E-04	0.00101	1.92E-09	0.89999	5.1359826
SCRM2_T208M	T208M	MA_10435231g0010	0.9	6.6801453	0.0351	0.0444439	0.00101	6.92E-05	0.00101	0.00E+00	0.00101	0.00E+00	0.25272	0.4531943
SCRM2_S238N	S238N	MA_10435231g0010	0.9	6.9390908	0.05953	0.0798303	0.00101	2.51E-05	0.00101	0.00E+00	0.00101	0.00E+00	0.00911	0.0104674
TCP2_F4S	F4S	MA_92659g0010	0.2461	0.4377259	0.00321	0.00353	0.00101	1.20E-10	0.00101	0.00E+00	0.00101	0.00E+00	0.63803	1.9760238
TCP2_V25A	V25A	MA_92659g0010	0.1101	0.1619267	0.00829	0.0094848	0.00101	2.74E-06	0.00101	2.18E-10	0.00101	8.44E-10	0.89999	5.3953807
TCP2_E32G	E32G	MA_92659g0010	0.0808	0.1127966	0.0067	0.0075799	0.00101	1.99E-06	0.00101	1.32E-10	0.00101	5.73E-10	0.89999	5.7775225
TCP2_E51K	E51K	MA_92659g0010	0.9	8.2469537	0.00101	0.0001536	0.00101	3.33E-13	0.00101	0.00E+00	0.00101	4.33E-14	0.00264	0.002883
TCP2_I53F	I53F	MA_92659g0010	0.7579	2.9050206	0.00101	0.0002009	0.00101	2.23E-13	0.00101	0.00E+00	0.00101	2.33E-14	0.03086	0.0386213
NAC036_D206E	D206E	MA_101849g0010	0.9	7.1059258	0.56717	1.5466731	0.02361	2.89E-02	0.00101	6.94E-07	0.00101	4.23E-06	0.89999	5.4945844
NAC036_T237M	T237M	MA_101849g0010	0.9	5.9875681	0.89999	5.2107547	0.79673	3.27E+00	0.09522	1.37E-01	0.00663	7.51E-03	0.89999	13.521499
EXPB3_K3R	K3R	MA_7354451g0010	0.9	12.011758	0.89999	4.9009516	0.00942	1.08E-02	0.00101	2.56E-05	0.00101	1.63E-05	0.89999	7.1191219
EXPB3_R6S	R6S	MA_7354451g0010	0.8347	3.6497194	0.48448	1.1474042	0.00101	1.97E-04	0.00101	9.30E-11	0.00101	4.42E-10	0.89999	7.9913455
EXPB3_C32R	C32R	MA_7354451g0010	0.9	4.6678307	0.00522	0.0058414	0.00359	3.97E-03	1.01E-03	3.56E-08	0.00101	1.07E-04	0.12507	0.1892671
FLZ6_L88V	L88V	MA_14341g0010	0.9	11.374357	0.01786	0.0214026	0.01842	2.21E-02	0.00101	5.09E-07	0.00101	7.60E-06	0.06004	0.0806038
VRLK1_A265V	A265V	MA_587505g0010	0.9	11.032665	0.89999	5.6330672	0.88508	4.2049855	0.04405	0.0570795	0.31754	0.6161669	0.83226	3.6237187
VRLK1_V283L	V283L	MA_587505g0010	0.9	13.19395	0.89999	10.859847	0.0046	5.12E-03	0.00101	8.20E-05	0.00267	2.91E-03	0.89999	12.620119
RPS2_S123N	S123N	MA_475302g0010	0.9	6.875651	0.00974	0.0112368	0.00101	1.61E-11	0.00101	0.00E+00	0.00101	0.00E+00	0.00119	0.0012757
RPS2_E166A	E166A	MA_475302g0010	0.9	11.677818	0.00448	0.0049875	0.00101	6.33E-13	0.00101	3.33E-15	0.00101	3.06E-13	0.00233	0.0025296

Gene name	Tukey's p-value S2 vs S4	Bonferroni p-value S2 vs S4	Tukey's p-value S2 vs S5	Bonferroni p-value S2 vs S5	Tukey's p-value S2 vs S6	Bonferroni p-value S2 vs S6	Tukey's p-value S3 vs S4	Bonferroni p-value S3 vs S4	Tukey's p-value S3 vs S5	Bonferroni p-value S3 vs S5	Tukey's p-value S3 vs S6	Bonferroni p-value S3 vs S6	Tukey's p-value S4 vs S5	Bonferroni p-value S4 vs S5	Tukey's p-value S4 vs S6	Bonferroni p-value S4 vs S6	Tukey's p-value S5 vs S6	Bonferroni p-value S5 vs S6
MYB3_T132R	0.00101	0.0002866	0.00101	0.00E+00	0.00101	0.00E+00	0.01416	0.0167036	0.00101	9.99E-15	0.00101	0.00E+00	0.00101	0.0001467	0.001	1.44E-08	0.03717	0.047318
LOV1_R156K	0.89999	7.0910793	0.89999	1.37E+01	0.00137	1.46E-03	0.89999	14.124039	0.89999	4.60E+00	0.00101	8.42E-05	0.89999	4.9076422	0.001	5.80E-05	0.00101	5.79E-05
LOV1_D190N	0.89999	6.2452656	0.18041	2.95E-01	0.00101	1.80E-05	0.51207	1.2670546	0.01062	1.23E-02	0.00101	2.59E-07	0.74445	2.7870975	0.001	9.97E-04	0.00571	0.0064232
SCRM2_T208M	0.00288	0.0031488	0.00101	2.97E-12	0.00101	6.66E-15	0.67395	2.2262969	0.00101	1.06E-04	0.00101	1.12E-07	0.0466	0.0607834	0.001	1.33E-04	0.12701	0.1927665
SCRM2_S238N	0.00101	1.41E-06	0.00101	0.00E+00	0.00101	0.00E+00	0.45082	1.0198796	0.00101	3.44E-07	0.00101	1.45E-08	0.00534	0.0059807	0.001	1.74E-04	0.51272	1.2701007
TCP2_F4S	0.00101	9.49E-05	0.00101	5.59E-08	0.00101	0.00E+00	0.0431	0.0557163	0.00139	1.50E-03	0.00101	1.03E-13	0.89999	9.9550509	0.001	7.58E-06	0.00101	1.87E-07
TCP2_V25A	0.10321	0.150322	0.00763	8.69E-03	0.00174	1.88E-03	0.59302	1.693677	0.23279	4.07E-01	0.05774	7.72E-02	0.89999	11.063088	0.7961	3.26E+00	0.83888	3.693338
TCP2_E32G	0.11513	0.1705974	0.00884	1.02E-02	0.00205	2.22E-03	0.59257	1.691056	0.22973	4.00E-01	0.05752	7.68E-02	0.89999	10.999381	0.7957	3.26E+00	0.84157	3.7218412
TCP2_E51K	0.00101	1.17E-10	0.00101	0.00E+00	0.00101	2.27E-11	0.03883	0.0496521	0.00101	5.35E-05	0.02435	2.99E-02	0.82337	3.5318492	0.9	1.37E+01	0.87929	4.1384781
TCP2_I53F	0.00101	7.27E-09	0.00101	0.00E+00	0.00101	1.49E-09	0.02786	0.0345557	0.00101	2.71E-05	0.01593	1.89E-02	0.81671	3.4639986	0.9	1.35E+01	0.88391	4.1916593
NAC036_D206E	0.19113	0.3168462	0.00101	2.10E-04	0.00101	3.24E-04	0.73195	2.680765	0.02404	2.95E-02	0.01654	1.97E-02	0.62414	1.8852176	0.4121	8.89E-01	0.89999	6.9681642
NAC036_T237M	0.89999	10.60103	0.64544	2.03E+00	0.11318	1.67E-01	0.89999	12.162953	0.77311	3.04E+00	0.18419	3.03E-01	0.89999	4.4701953	0.2571	4.63E-01	0.60649	1.7746104
EXPB3_K3R	0.03005	0.0375134	0.00101	3.17E-04	0.00101	1.23E-04	0.2375	0.417652	0.01826	2.19E-02	0.0048	5.36E-03	0.89999	8.2555587	0.7049	2.46E+00	0.88391	4.1915974
EXPB3_R6S	0.02506	0.0308244	0.00101	3.37E-06	0.00101	1.90E-06	0.17021	0.2737278	0.00101	3.89E-04	0.00101	1.09E-04	0.6542	2.0857852	0.2455	4.36E-01	0.85008	3.813265
EXPB3_C32R	0.10978	0.1614217	0.00101	9.58E-05	0.01019	1.18E-02	0.89999	14.621978	0.73263	2.69E+00	0.89999	6.94E+00	0.66994	2.1971697	0.9	6.41E+00	0.89999	9.1855594
FLZ6_L88V	0.06363	0.0860072	0.00101	1.82E-05	0.00101	9.26E-05	0.89999	14.028657	0.71543	2.54E+00	0.60262	1.75E+00	0.63245	1.9391767	0.5326	1.37E+00	0.89999	9.3941199
VRLK1_A265V	0.89999	7.1254889	0.175	0.2836421	0.56364	1.5273451	0.42623	0.9351913	0.00381	0.0042099	0.06251	0.0843104	0.64787	2.0422428	0.9	5.4142459	0.89999	10.204831
VRLK1_V283L	0.01182	0.0137868	0.00101	5.13E-04	0.00743	8.45E-03	0.03127	0.03918	0.00274	3.00E-03	0.02127	2.58E-02	0.89999	13.291233	0.9	1.39E+01	0.89999	14.528704
RPS2_S123N	0.00101	7.76E-13	0.00101	0.00E+00	0.00101	0.00E+00	0.00772	0.0088055	0.00101	1.25E-05	0.00101	2.90E-05	0.89999	5.6641515	0.7174	2.56E+00	0.89999	6.5902257
RPS2_E166A	0.00101	3.73E-13	0.00101	3.33E-15	0.00101	1.87E-13	0.00376	0.0041582	0.01089	1.26E-02	0.0032	3.51E-03	0.89999	4.3988266	0.9	1.49E+01	0.88747	4.2329841

Significant p-values (p-value<0.05) are highlighted

Table S5 Hardy Weinberg Equilibrium(HWE) for genotype frequencies of missense mutations in the nine candidate DEGs in response to SHADE in Norway spruce that show cline

Gene name	SNP	Gene_Id	S1 (lat 55-57) HWE p-value	S2 (lat 58) HWE p-value	S3 (lat 59-60) HWE p-value	S4 (lat 61-62) HWE p-value	S5 (lat 63-64) HWE p-value	S6 (lat 65-67) HWE p-value
MYB3_T132R	T132R	MA_7115g0010	0.6305073	0.7079489	0.09879334	0.8456276	0.5690967	0.2102694
LOV1_R156K	R156K	MA_16619g0010	0.5444456	0.7613816	0.7349385	0.8755682	0.3548293	0.418793
LOV1_D190N	D190N	MA_16619g0010	0.5665429	**0.00543298**	0.420296	**0.01394043**	**0.001147745**	**0.02871752**
SCRM2_T208M	T208M	MA_10435231g0010	0.5657677	0.85023	0.05895691	0.05068066	0.4412498	0.2792934
SCRM2_S238N	S238N	MA_10435231g0010	0.08930325	0.2158439	**0.01860447**	0.1180562	0.4398115	0.1794888
TCP2_F4S	F4S	MA_92659g0010	**0.001170066**	**0.0171661**	**0.003380215**	0.4064667	**0.01912685**	**0.000335543**
TCP2_V25A	V25A	MA_92659g0010	0.7798684	0.6814202	0.2724744	1	0.6166626	0.1421518
TCP2_E32G	E32G	MA_92659g0010	0.7805884	0.6788518	0.2724744	1	0.6166373	0.1421518
TCP2_E51K	E51K	MA_92659g0010	0.3382155	**0.00067806**	0.1981032	1	0.7584974	0.1178837
TCP2_I53F	I53F	MA_92659g0010	0.3402181	**0.00071409**	0.1205355	1	0.758248	0.1163396
NAC036_D206E	D206E	MA_101849g0010	0.07351378	1	0.3554533	0.08390553	0.05158337	**0.03029547**
NAC036_T237M	T237M	MA_101849g0010	0.3934845	0.4284897	0.5779759	0.3071086	0.6738657	0.8415818
EXPB3_K3R	K3R	MA_7354451g0010	0.07548853	0.2311453	0.1825768	1	**0.000137283**	**0.01557581**
EXPB3_R6S	R6S	MA_7354451g0010	0.1956392	0.4769058	**0.0299857**	0.8717671	**4.78E-06**	**0.01553658**
EXPB3_C32R	C32R	MA_7354451g0010	1	1	1	1	1	1
FLZ6_L88V	L88V	MA_14341g0010	**7.04E-08**	**0.00107311**	0.05147672	**0.02159798**	**1.80E-05**	**0.002130486**
VRLK1_A265V	A265V	MA_587505g0010	0.1128199	0.2850072	0.338132	1	0.1760774	0.3675938
VRLK1_V283L	V283L	MA_587505g0010	**0.0179411**	0.4710266	**0.001521785**	**0.01064617**	**2.25E-06**	**0.03230442**
RPS2_S123N	S123N	MA_475302g0010	**3.62E-09**	**0.00015184**	**0.000284223**	**2.97E-09**	**7.97E-18**	**3.74E-08**
RPS2_E166A	E166A	MA_475302g0010	**2.82E-10**	**0.00047369**	**0.000236755**	**6.31E-06**	**5.82E-11**	**5.95E-06**

Significant p-values (p-value<0.05) are highlighted

Table S6 Allele frequencies of missense mutations in the nine candidate DEGs in response to SHADE in Norway spruce that do not show cline

					Population-wise allele frequency					
**ContigID:SnpPosition**	**Gene_ID**	**Gene name**	**Amino acid change**	**Allele**	**S1**	**S2**	**S3**	**S4**	**S5**	**S6**
MA_7115:46740	MA_7115g0010	MYB3	Arg162Gln	**Reference (G)**	0.995851	0.992788	0.989247	0.990148	0.99195	0.99095
				**Alternate (A)**	0.00414938	0.00721154	0.0107527	0.00985222	0.00805009	0.00904977
MA_7115:46905	MA_7115g0010	MYB3	Pro107Leu	**Reference (C)**	0.985356	0.992857	0.989247	0.987923	0.992958	0.988636
				**Alternate (T)**	0.0146444	0.00714286	0.0107527	0.0120773	0.00704225	0.0113636
MA_7115:46944	MA_7115g0010	MYB3	His94Leu	**Reference (A)**	0.642857	0.617925	0.6	0.611111	0.653641	0.702703
				**Alternate (T)**	0.357143	0.382075	0.4	0.388889	0.346359	0.297297
MA_7115:46972	MA_7115g0010	MYB3	Pro85Thr	**Reference (C)**	0.82582	0.82783	0.818182	0.85782	0.879159	0.847534
				**Alternate (A)**	0.17418	0.17217	0.181818	0.14218	0.120841	0.152466
MA_7115:47037	MA_7115g0010	MYB3	His63Arg	**Reference (A)**	0.806122	0.775943	0.794118	0.754717	0.769702	0.860987
				**Alternate (G)**	0.193878	0.224057	0.205882	0.245283	0.230298	0.139013
MA_7115:47067	MA_7115g0010	MYB3	Pro53Arg	**Reference (C)**	0.975309	0.96934	0.975806	0.99763	0.999123	0.997738
				**Alternate (G)**	0.0246914	0.0306604	0.0241935	0.00236967	0.000877193	0.00226244
MA_16619:4719	MA_16619g0010	LOV1	Glu41Lys	**Reference (G)**	0.940816	0.924528	0.927807	0.898058	0.914613	0.842342
				**Alternate (A)**	0.0591837	0.0754717	0.0721925	0.101942	0.0853873	0.157658
MA_16619:4858	MA_16619g0010	LOV1	Asp87Val	**Reference (A)**	0.984375	0.992308	0.983051	0.992063	0.98	0.983333
				**Alternate (T)**	0.015625	0.00769231	0.0169492	0.00793651	0.02	0.0166667
MA_10435231:4572	MA_10435231g0010	SCRM2	Thr26Ala	**Reference (A)**	0.962963	0.950472	0.981183	0.992788	0.996466	0.993243
				**Alternate (G)**	0.037037	0.0495283	0.0188172	0.00721154	0.00353357	0.00675676
MA_10435231:4578	MA_10435231g0010	SCRM2	Leu28Val	**Reference (C)**	0.95082	0.931604	0.882353	0.879808	0.838028	0.80543
				**Alternate (G)**	0.0491803	0.0683962	0.117647	0.120192	0.161972	0.19457
MA_92659:15358	MA_92659g0010	TCP2	Gly364Val	**Reference (G)**	0.955752	0.97	0.945355	0.968599	0.936364	0.956019
				**Alternate (T)**	0.0442478	0.03	0.0546448	0.031401	0.0636364	0.0439815
MA_101849:25488	MA_101849g0010	NAC036	Ser56Leu	**Reference (C)**	0.835417	0.832524	0.808743	0.84	0.795495	0.819635
				**Alternate (T)**	0.164583	0.167476	0.191257	0.16	0.204505	0.180365
MA_101849:25632	MA_101849g0010	NAC036	Thr104Ile	**Reference (C)**	0.668724	0.670616	0.693548	0.71875	0.737676	0.702703
				**Alternate (T)**	0.331276	0.329384	0.306452	0.28125	0.262324	0.297297
MA_101849:25646	MA_101849g0010	NAC036	Ser109Pro	**Reference (T)**	0.199588	0.130332	0.172043	0.18599	0.149558	0.144796
				**Alternate (C)**	0.800412	0.869668	0.827957	0.81401	0.850442	0.855204
MA_101849:25689	MA_101849g0010	NAC036	Lys123Arg	**Reference (A)**	0.971193	0.966667	0.97043	0.983333	0.977837	0.988636
				**Alternate (G)**	0.0288066	0.0333333	0.0295699	0.0166667	0.0221631	0.0113636
MA_14341:1048	MA_14341g0010	FLZ6	Val14Ile	**Reference (G)**	0.781633	0.739437	0.666667	0.71934	0.757867	0.578829
				**Alternate (A)**	0.218367	0.260563	0.333333	0.28066	0.242133	0.421171
MA_14341:1231	MA_14341g0010	FLZ6	Ser75Pro	**Reference (T)**	0.161088	0.135468	0.181564	0.232673	0.272556	0.172018
				**Alternate (C)**	0.838912	0.864532	0.818436	0.767327	0.727444	0.827982
MA_14341:1244	MA_14341g0010	FLZ6	Gly79Val	**Reference (G)**	0.975104	0.985714	0.983696	0.995215	0.987365	0.99095
				**Alternate (T)**	0.0248963	0.0142857	0.0163043	0.00478469	0.0126354	0.00904977
MA_14341:1265	MA_14341g0010	FLZ6	Ala86Val	**Reference (C)**	0.981481	0.975962	0.964088	0.907317	0.871087	0.945701
				**Alternate (T)**	0.0185185	0.0240385	0.0359116	0.0926829	0.128913	0.0542986
MA_587505:1178	MA_587505g0010	VRLK1	Thr271Pro	**Reference (A)**	0.959016	0.959716	0.948925	0.956522	0.953488	0.948198
				**Alternate (C)**	0.0409836	0.0402844	0.0510753	0.0434783	0.0465116	0.0518018
MA_587505:1244	MA_587505g0010	VRLK1	Pro249Ala	**Reference (C)**	0.97479	0.956311	0.945355	0.974874	0.973535	0.953917
				**Alternate (G)**	0.0252101	0.0436893	0.0546448	0.0251256	0.026465	0.0460829
MA_475302:3505	MA_475302g0010	RPS2	Glu150Asp	**Reference (G)**	0.932773	0.936893	0.909341	0.8225	0.76588	0.873832
				**Alternate (T)**	0.0672269	0.0631068	0.0906593	0.1775	0.23412	0.126168
MA_475302:3524	MA_475302g0010	RPS2	Gly144Asp	**Reference (G)**	0.981172	0.985366	0.958791	0.938424	0.950271	0.943662
				**Alternate (A)**	0.0188285	0.0146341	0.0412088	0.0615764	0.0497288	0.056338
MA_475302:3557	MA_475302g0010	RPS2	Val133Ala	**Reference (T)**	0.42766	0.376238	0.544199	0.733831	0.758272	0.68599
				**Alternate (C)**	0.57234	0.623762	0.455801	0.266169	0.241728	0.31401

Table S7 Allele frequencies of synonymous mutations in the nine candidate DEGs in response to SHADE in Norway spruce that show mild cline

					Population-wise allele frequency					
**ContigID:SnpPosition**	**Gene_ID**	**Gene name**	**Amino acid change**	**Allele**	**S1**	**S2**	**S3**	**S4**	**S5**	**S6**
MA_92659:14474	MA_92659g0010	TCP2	Tyr69Tyr	**Reference (T)**	T:0.964912	T:0.958115	T:0.985955	T:0.992537	T:0.992767	T:0.992991
				**Alternate (C)**	C:0.0350877	C:0.0418848	C:0.0140449	C:0.00746269	C:0.00723327	C:0.00700935
MA_92659:15344	MA_92659g0010	TCP2	Arg359Arg	**Reference (C)**	C:0.948661	C:0.8925	C:0.826705	C:0.728856	C:0.70941	C:0.564286
				**Alternate (A)**	A:0.0513393	A:0.1075	A:0.173295	A:0.271144	A:0.29059	A:0.435714
MA_16619:4700	MA_16619g0010	LOV1	Ala34Ala	**Reference (G)**	G:0.979508	G:0.992925	G:0.967914	G:0.954327	G:0.939367	G:0.91704
				**Alternate (A)**	A:0.0204918	A:0.00707547	A:0.0320856	A:0.0456731	A:0.0606327	A:0.0829596
MA_14341:1032	MA_14341g0010	FLZ6	Phe8Phe	**Reference (T)**	T:0.963265	T:0.959906	T:0.938172	T:0.915094	T:0.925569	T:0.849099
				**Alternate (C)**	C:0.0367347	C:0.0400943	C:0.061828	C:0.0849057	C:0.0744308	C:0.150901
MA_101849:25612	MA_101849g0010	NAC036	Phe97Phe	**Reference (T)**	T:0.884774	T:0.850711	T:0.859459	T:0.86019	T:0.847711	T:0.808559
				**Alternate (C)**	C:0.115226	C:0.149289	C:0.140541	C:0.13981	C:0.152289	C:0.191441
MA_101849:25633	MA_101849g0010	NAC036	Thr104Thr	**Reference (T)**	T:0.584362	T:0.535545	T:0.58871	T:0.581731	T:0.545936	T:0.513514
				**Alternate (G)**	G:0.415638	G:0.464455	G:0.41129	G:0.418269	G:0.454064	G:0.486486
MA_101849:25735	MA_101849g0010	NAC036	Tyr138Tyr	**Reference (T)**	T:0.965021	T:0.944175	T:0.951613	T:0.953431	T:0.928187	T:0.884091
				**Alternate (C)**	C:0.0349794	C:0.0558252	C:0.0483871	C:0.0465686	C:0.0718133	C:0.115909
MA_101849:25762	MA_101849g0010	NAC036	Phe147Phe	**Reference (C)**	C:0.944215	C:0.93	C:0.942935	C:0.936893	C:0.927549	C:0.974654
				**Alternate (T)**	T:0.0557851	T:0.07	T:0.0570652	T:0.0631068	T:0.0724508	T:0.0253456

Table S8 Allele frequencies of synonymous mutations in the nine candidate DEGs in response to SHADE in Norway spruce that do not show cline

					Population-wise allele frequency					
**ContigID:SnpPosition**	**Gene_ID**	**Gene name**	**Amino acid change**	**Allele**	**S1**	**S2**	**S3**	**S4**	**S5**	**S6**
MA_7115:46700	MA_7115g0010	MYB3	Leu175Leu	**Reference (A)**	0.914583	0.87799	0.863388	0.856061	0.85118	0.920814
				**Alternate (G)**	0.0854167	0.12201	0.136612	0.143939	0.14882	0.0791855
MA_16619:4622	MA_16619g0010	LOV1	Arg8Arg	**Reference (G)**	0.983607	0.988152	0.962366	0.963768	0.983186	0.986547
				**Alternate (A)**	0.0163934	0.0118483	0.0376344	0.0362319	0.0168142	0.0134529
MA_16619:4679	MA_16619g0010	LOV1	His27His	**Reference (C)**	0.979508	0.985782	0.983957	0.976077	0.972711	0.975336
				**Alternate (T)**	0.0204918	0.014218	0.0160428	0.0239234	0.0272887	0.0246637
MA_10435231:4637	MA_10435231g0010	SCRM2	Cys47Cys	**Reference (C)**	0.967347	0.978673	0.967914	0.96875	0.976232	0.975113
				**Alternate (T)**	0.0326531	0.021327	0.0320856	0.03125	0.0237676	0.0248869
MA_10435231:4643	MA_10435231g0010	SCRM2	Leu49Leu	**Reference (G)**	1	0.990521	0.989305	0.973558	0.970018	0.9819
				**Alternate (T)**	0	0.00947867	0.0106952	0.0264423	0.0299824	0.0180995
MA_92659:14336	MA_92659g0010	TCP2	Asn23Asn	**Reference (T)**	0.933884	0.914286	0.918919	0.910628	0.917845	0.909502
				**Alternate (C)**	0.0661157	0.0857143	0.0810811	0.089372	0.0821555	0.0904977
MA_101849:25543	MA_101849g0010	NAC036	Gly74Gly	**Reference (T)**	0.197531	0.135071	0.183784	0.193627	0.150995	0.145455
				**Alternate (C)**	0.802469	0.864929	0.816216	0.806373	0.849005	0.854545
MA_101849:25549	MA_101849g0010	NAC036	Cys76Cys	**Reference (T)**	0.981557	0.978774	0.964865	0.978365	0.992895	0.995495
				**Alternate (C)**	0.0184426	0.0212264	0.0351351	0.0216346	0.0071048	0.0045045
MA_7354451:133	MA_7354451g0010	EXPB3	Leu28Leu	**Reference (T)**	0.973361	0.966981	0.978495	0.938679	0.93838	0.929545
				**Alternate (C)**	0.0266393	0.0330189	0.0215054	0.0613208	0.0616197	0.0704545
MA_14341:1083	MA_14341g0010	FLZ6	Pro25Pro	**Reference (C)**	0.826531	0.800469	0.763441	0.816038	0.847636	0.754505
				**Alternate (T)**	0.173469	0.199531	0.236559	0.183962	0.152364	0.245495

Table S9 Allele frequencies of missense mutations in the DEGs other than nine candidate genes in response to SHADE in Norway spruce

					Population-wise allele frequency					
**ContigID:SnpPosition**	**Gene_ID**	**Gene name**	**Amino acid change**	**Allele**	**S1**	**S2**	**S3**	**S4**	**S5**	**S6**
MA_86183:6248	MA_86183g0010	BIG GRAIN 3 (BG3)	Ala30Val	**Reference (C)**<br>**Alternate (T)**	C:0.952869<br>T:0.0471311	C:0.95283<br>T:0.0471698	C:0.935135<br>T:0.0648649	C:0.884058<br>T:0.115942	C:0.864675<br>T:0.135325	C:0.819005<br>T:0.180995
MA_10351640:1873	MA_10351640g0010	S-ADENOSYL METHIONINE TRANSPORTER-LIKE (SAMTL)	Gln119Lys	**Reference (C)**<br>**Alternate (A)**	C:0.972803<br>A:0.0271967	C:0.971154<br>A:0.0288462	C:0.964865<br>A:0.0351351	C:0.927184<br>A:0.0728155	C:0.921147<br>A:0.078853	C:0.925676<br>A:0.0743243
MA_15920:34530	MA_15920g0010	MYB-like HTH transcriptional regulator family protein	Val9Met	**Reference (G)**<br>**Alternate (A)**	G:0.959916<br>A:0.0400844	G:0.954545<br>A:0.0454545	G:0.949153<br>A:0.0508475	G:0.914216<br>A:0.085784	G:0.869091<br>A:0.130909	G:0.815421<br>A:0.184579
MA_20782:9490	MA_20782g0010	Tetratricopeptide repeat (TPR)-like superfamily protein	Ala180Val	**Reference (C)**<br>**Alternate (T)**	C:0.212185<br>T:0.787815	C:0.220588<br>T:0.779412	C:0.287634<br>T:0.712366	C:0.355392<br>T:0.644608	C:0.366667<br>T:0.633333	C:0.372146<br>T:0.627854
MA_80461:1299	MA_80461g0010	Disease resistance protein	Asp154Ala	**Reference (A)**<br>**Alternate (C)**	A:0.991416<br>C:0.00858369	A:0.995074<br>C:0.00492611	A:0.991279<br>C:0.00872093	A:0.992105<br>C:0.00789474	A:0.985102<br>C:0.0148976	A:0.987805<br>C:0.0121951
MA_227006:3453	MA_227006g0010	Diphthamide synthesis DPH2 family protein	Arg21Trp	**Reference (C)**<br>**Alternate (T)**	C:0.946721<br>T:0.0532787	C:0.933962<br>T:0.0660377	C:0.884409<br>T:0.115591	C:0.866995<br>T:0.133005	C:0.859929<br>T:0.140071	C:0.777027<br>T:0.222973
MA_423032:1006	MA_423032g0010	Disease resistance protein	Ala51Ser	**Reference (G)**<br>**Alternate (T)**	G:0.981405<br>T:0.018595	G:0.981221<br>T:0.0187793	G:0.986559<br>T:0.0134409	G:0.973171<br>T:0.0268293	G:0.968582<br>T:0.0314183	G:0.965438<br>T:0.0345622
MA_447489:1980	MA_447489g0010	RING-H2 FINGER A2A (RHA2A)	Ser92Thr	**Reference (T)**<br>**Alternate (A)**	T:0.995885<br>A:0.00411523	T:0.995283<br>A:0.00471698	T:0.986559<br>A:0.0134409	T:0.983412<br>A:0.0165877	T:0.956714<br>A:0.0432862	T:0.945946<br>A:0.0540541
MA_904305:7873	MA_904305g0010	Tetratricopeptide repeat (TPR)-like superfamily protein	Arg55Ser	**Reference (G)**<br>**Alternate (C)**	G:0.973333<br>C:0.0266667	G:0.976923<br>C:0.0230769	G:0.956395<br>C:0.0436047	G:0.937173<br>C:0.0628272	G:0.913424<br>C:0.0865759	G:0.917073<br>C:0.0829268
MA_904305:8387	MA_904305g0010	Tetratricopeptide repeat (TPR)-like superfamily protein	Ala227Thr	**Reference (G)**<br>**Alternate (A)**	G:0.971311<br>A:0.0286885	G:0.973934<br>A:0.0260664	G:0.957219<br>A:0.0427807	G:0.949761<br>A:0.0502392	G:0.938163<br>A:0.0618375	G:0.938914<br>A:0.061086
MA_918643:2547	MA_918643g0010	Protein kinase superfamily protein	Try87His	**Reference (T)**<br>**Alternate (C)**	T:0.653061<br>C:0.346939	T:0.601415<br>C:0.398585	T:0.521505<br>C:0.478495	T:0.464789<br>C:0.535211	T:0.367544<br>C:0.632456	T:0.335586<br>C:0.664414
MA_945672:6851	MA_945672g0010	Oxygenase superfamily protein	Asp71Val	**Reference (A)**<br>**Alternate (T)**	A:0.900826<br>T:0.0991736	A:0.887019<br>T:0.112981	A:0.852151<br>T:0.147849	A:0.816425<br>T:0.183575	A:0.833037<br>T:0.166963	A:0.718182<br>T:0.281818
MA_945672:7014	MA_945672g0010	Oxygenase superfamily protein	Ser17Gly	**Reference (A)**<br>**Alternate (G)**	A:0.904564<br>G:0.0954357	A:0.889952<br>G:0.110048	A:0.860215<br>G:0.139785	A:0.822967<br>G:0.177033	A:0.831541<br>G:0.168459	A:0.726852<br>G:0.273148
MA_945672:7341	MA_945672g0010	Oxygenase superfamily protein	Arg115Ser	**Reference (C)**<br>**Alternate (A)**	C:0.971074<br>A:0.0289256	C:0.978469<br>A:0.0215311	C:0.959677<br>A:0.0403226	C:0.934466<br>A:0.065534	C:0.935599<br>A:0.0644007	C:0.936364<br>A:0.0636364
MA_10430253:15461	MA_10430253g0010	Alpha/beta-Hydrolases superfamily protein	Phe61Tyr	**Reference (T)**<br>**Alternate (A)**	T:0.168103<br>A:0.831897	T:0.207729<br>A:0.792271	T:0.265537<br>A:0.734463	T:0.308081<br>A:0.691919	T:0.313528<br>A:0.686472	T:0.375<br>A:0.625
MA_10432704:73817	MA_10432704g0020	CHLORORESPIRATORY REDUCTION22 (CRR22)	Ala219Val	**Reference (C)**<br>**Alternate (T)**	C:0.997908<br>T:0.00209205	C:0.992857<br>T:0.00714286	C:0.991848<br>T:0.00815217	C:0.9875<br>T:0.0125	C:0.979204<br>T:0.0207957	C:0.96789<br>T:0.0321101
MA_96757:12081	MA_96757g0010	PEROXIDASE 52 (PRX52)	Ala55Thr	**Reference (G)**<br>**Alternate (A)**	G:0.993878<br>A:0.00612245	G:0.992891<br>A:0.007109	G:0.997312<br>A:0.00268817	G:0.978673<br>A:0.021327	G:0.975177<br>A:0.0248227	G:0.975225<br>A:0.0247748
MA_90634:8021	MA_90634g0010	Aluminum activated malate transporter family protein	Pro43Ser	**Reference (C)**<br>**Alternate (T)**	C:0.997925<br>T:0.00207469	C:0.987923<br>T:0.0120773	C:0.986486<br>T:0.0135135	C:0.981043<br>T:0.0189573	C:0.97331<br>T:0.0266904	C:0.958904<br>T:0.0410959
MA_89572:19598	MA_89572g0010	SET DOMAIN PROTEIN 35 (SDG35)	Leu35Ser	**Reference (A)**<br>**Alternate (G)**	A:0.738589<br>G:0.261411	A:0.722222<br>G:0.277778	A:0.765027<br>G:0.234973	A:0.77451<br>G:0.22549	A:0.852415<br>G:0.147585	A:0.844037<br>G:0.155963
MA_8433628:1321	MA_8433628g0010	Plant invertase/pectin methylesterase inhibitor superfamily protein	Met85Leu	**Reference (T)**<br>**Alternate (A)**	T:0.970464<br>A:0.0295359	T:0.953202<br>A:0.046798	T:0.980447<br>A:0.0195531	T:0.987113<br>A:0.0128866	T:0.996176<br>A:0.00382409	T:0.995327<br>A:0.0046729
MA_83446:30566	MA_83446g0020	purple acid phosphatase 27;(source:Araport11)	Trp38Ser	**Reference (G)**<br>**Alternate (C)**	G:0.868313<br>C:0.131687	G:0.848341<br>C:0.151659	G:0.892473<br>C:0.107527	G:0.929612<br>C:0.0703883	G:0.974245<br>C:0.0257549	G:0.979638<br>C:0.020362
MA_8344:30649	MA_83446g0020	purple acid phosphatase 27;(source:Araport11)	Glu66Lys	**Reference (G)**<br>**Alternate (A)**	G:0.865702<br>A:0.134298	G:0.841346<br>A:0.158654	G:0.895161<br>A:0.104839	G:0.919903<br>A:0.0800971	G:0.975979<br>A:0.0240214	G:0.972727<br>A:0.0272727
MA_8344:30651	MA_83446g0020	purple acid phosphatase 27;(source:Araport11)	Glu66Asp	**Reference (G)**<br>**Alternate (T)**	G:0.865702<br>T:0.134298	G:0.841346<br>T:0.158654	G:0.895161<br>T:0.104839	G:0.92029<br>T:0.0797101	G:0.975133<br>T:0.0248668	G:0.972851<br>T:0.0271493
MA_8344:30830	MA_83446g0020	purple acid phosphatase 27;(source:Araport11)	Leu126His	**Reference (T)**<br>**Alternate (A)**	T:0.880753<br>A:0.119247	T:0.885167<br>A:0.114833	T:0.905405<br>A:0.0945946	T:0.871287<br>A:0.128713	T:0.857923<br>A:0.142077	T:0.818605<br>A:0.181395
MA_58710:10558	MA_58710g0010	Subtilase family protein	Cys24Tyr	**Reference (G)**<br>**Alternate (A)**	G:0.761224<br>A:0.238776	G:0.755924<br>A:0.244076	G:0.737838<br>A:0.262162	G:0.71327<br>A:0.28673	G:0.738011<br>A:0.261989	G:0.717489<br>A:0.282511
MA_5587:58879	MA_5587g0010	glycosyltransferase family protein 2	Cys294Ser	**Reference (A)**<br>**Alternate (T)**	A:0.932735<br>T:0.0672646	A:0.957286<br>T:0.0427136	A:0.921512<br>T:0.0784884	A:0.959459<br>T:0.0405405	A:0.954373<br>T:0.0456274	A:0.987923<br>T:0.0120773
MA_5587:58921	MA_5587g0010	glycosyltransferase family protein 2	Llu280Lys	**Reference (C)**<br>**Alternate (T)**	C:0.886076<br>T:0.113924	C:0.90625<br>T:0.09375	C:0.879121<br>T:0.120879	C:0.935<br>T:0.065	C:0.933028<br>T:0.0669725	C:0.962441<br>T:0.0375587

MA_5587:58936	MA_5587g0010	glycosyltransferase family protein 2	Gln275Lys	**Reference (G)**<br>**Alternate (T)**	G:0.866109<br>T:0.133891	G:0.900474<br>T:0.0995261	G:0.863388<br>T:0.136612	G:0.921182<br>T:0.0788177	G:0.925551<br>T:0.0744485	G:0.949074<br>T:0.0509259
MA_5587:58946	MA_5587g0010	glycosyltransferase family protein 2	Gln271His	**Reference (C)**<br>**Alternate (A)**	C:0.983402<br>A:0.0165975	C:0.973934<br>A:0.0260664	C:0.978261<br>A:0.0217391	C:0.987745<br>A:0.0122549	C:0.99543<br>A:0.00457038	C:0.997706<br>A:0.0022935<br>8
MA_5587:58965	MA_5587g0010	glycosyltransferase family protein 2	Ala265Val	**Reference (G)**<br>**Alternate (A)**	G:0.968487<br>A:0.0315126	MG:0.966667<br>A:0.0333333	G:0.983784<br>A:0.0162162	G:0.97<br>A:0.03	G:0.979204<br>A:0.0207957	G:0.990868<br>A:0.0091324<br>2
MA_5587:59010	MA_5587g0010	glycosyltransferase family protein 2	Ile250Thr	**Reference (A)**<br>**Alternate (G)**	A:0.968619<br>G:0.0313808	A:0.968447<br>G:0.0315534	A:0.989011<br>G:0.010989	A:0.9675<br>G:0.0325	A:0.974074<br>G:0.0259259	A:0.979167<br>G:0.0208333
MA_5587:59031	MA_5587g0010	glycosyltransferase family protein 2	Pro243Leu	**Reference (G)**<br>**Alternate (A)**	G:0.968354<br>A:0.0316456	G:0.968293<br>A:0.0317073	G:0.986111<br>A:0.0138889	G:0.972081<br>A:0.0279188	G:0.975791<br>A:0.0242086	G:0.981132<br>A:0.0188679
MA_52897:17848	MA_52897g0010	Protein kinase superfamily protein	Gly194Arg	**Reference (C)**<br>**Alternate (T)**	C:0.57377<br>T:0.42623	C:0.561905<br>T:0.438095	C:0.601093<br>T:0.398907	C:0.629268<br>T:0.370732	C:0.590909<br>T:0.409091	C:0.625<br>T:0.375
MA_52897:18331	MA_52897g0010	Protein kinase superfamily protein	Val33Ile	**Reference (C)**<br>**Alternate (T)**	C:0.89959<br>T:0.10041	C:0.899522<br>T:0.100478	C:0.948925<br>T:0.0510753	C:0.939904<br>T:0.0600962	C:0.949558<br>T:0.0504425	C:0.958904<br>T:0.0410959
MA_510832:1492	MA_510832g0010	hAT dimerization domain-containing protein / transposase-like protein	Ala52Thr	**Reference (C)**<br>**Alternate (T)**	C:0.985656<br>T:0.0143443	C:0.971564<br>T:0.028436	C:0.973118<br>T:0.0268817	C:0.898551<br>T:0.101449	C:0.919183<br>T:0.0808171	C:0.910314<br>T:0.0896861
MA_4984597:670	MA_4984597g0010	Eukaryotic aspartyl protease family protein	Val224Met	**Reference (G)**<br>**Alternate (A)**	G:0.983673<br>A:0.0163265	G:0.971698<br>A:0.0283019	G:0.97027<br>A:0.0297297	G:0.952381<br>A:0.047619	G:0.944938<br>A:0.0550622	G:0.950673<br>A:0.0493274
MA_38922:6655	MA_38922g0010	HEAVY METAL ASSOCIATED ISOPRENYLATED PLANT PROTEIN 20 (HIPP20)	Phe2Cys	**Reference (T)**<br>**Alternate (G)**	T:0.946721<br>G:0.0532787	T:0.940758<br>G:0.0592417	T:0.943243<br>G:0.0567568	T:0.969484<br>G:0.0305164	T:0.97242<br>G:0.0275801	T:0.974886<br>G:0.0251142
MA_31254:22109	MA_31254g0010	U3 small nucleolar RNA-associated-like protein	Glu30Asp	**Reference (T)**<br>**Alternate (G)**	T:0.724066<br>G:0.275934	T:0.758294<br>G:0.241706	T:0.76087<br>G:0.23913	T:0.806763<br>G:0.193237	T:0.814286<br>G:0.185714	T:0.809417<br>G:0.190583
MA_1925:30754	MA_1925g0010	LTPG15	Pro6Leu	**Reference (G)**<br>**Alternate (A)**	G:0.836735<br>A:0.163265	G:0.866197<br>A:0.133803	G:0.897849<br>A:0.102151	G:0.930622<br>A:0.069378	G:0.941441<br>A:0.0585586	G:0.928241<br>A:0.0717593
MA_17793:51035	MA_17793g0010	PLASMA MEMBRANE INTRINSIC PROTEIN 2;8 (PIP2;8)	Gly37Glu	**Reference (G)**<br>**Alternate (A)**	G:0.958678<br>A:0.0413223	G:0.913043<br>A:0.0869565	G:0.932432<br>A:0.0675676	G:0.874408<br>A:0.125592	G:0.855098<br>A:0.144902	G:0.772936<br>A:0.227064
MA_17793:51051	MA_17793g0010	PLASMA MEMBRANE INTRINSIC PROTEIN 2;8 (PIP2;8)	Lys42Asn	**Reference (G)**<br>**Alternate (T)**	G:0.966805<br>T:0.033195	G:0.978049<br>T:0.0219512	G:0.991892<br>T:0.00810811	G:0.990431<br>T:0.00956938	G:0.985714<br>T:0.0142857	G:0.990826<br>T:0.0091743<br>1
MA_169781:13449	MA_169781g0010	SPla/RYanodine receptor (SPRY) domain-containing protein	Arg46Ile	**Reference (C)**<br>**Alternate (A)**	C:0.997826<br>A:0.00217391	C:0.976923<br>A:0.0230769	C:0.988827<br>A:0.0111732	C:0.974619<br>A:0.0253807	C:0.943916<br>A:0.0560837	C:0.947005<br>A:0.0529954
MA_15382:33084	MA_15382g0010	F-box/RNI-like superfamily protein	Leu249Gln	**Reference (T)**<br>**Alternate (A)**	T:0.991632<br>A:0.0083682	T:0.990476<br>A:0.00952381	T:0.98913<br>A:0.0108696	T:0.956731<br>A:0.0432692	T:0.925267<br>A:0.0747331	T:0.958716<br>A:0.0412844
MA_15382:33180	MA_15382g0010	F-box/RNI-like superfamily protein	Leu281Pro	**Reference (T)**<br>**Alternate (C)**	T:0.958678<br>C:0.0413223	T:0.976303<br>C:0.0236967	T:0.9<br>C:0.1	T:0.865385<br>C:0.134615	T:0.840708<br>C:0.159292	T:0.763636<br>C:0.236364
MA_15382:33267	MA_15382g0010	F-box/RNI-like superfamily protein	Gly310Glu	**Reference (G)**<br>**Alternate (A)**	G:0.689956<br>A:0.310044	G:0.711905<br>A:0.288095	G:0.585165<br>A:0.414835	G:0.556931<br>A:0.443069	G:0.567935<br>A:0.432065	G:0.461187<br>A:0.538813
MA_14234:43020	MA_14234g0010	Adenine nucleotide alpha hydrolases-like superfamily protein	Arg25Gly	**Reference (C)**<br>**Alternate (G)**	C:0.985417<br>G:0.0145833	C:0.995098<br>G:0.00490196	C:0.975543<br>G:0.0244565	C:0.977273<br>G:0.0227273	C:0.962523<br>G:0.0374771	C:0.956019<br>G:0.0439815
MA_129478:6147	MA_129478g0010	BRASSINOSTEROID-RESPONSIVE RING-H2 (BRH1)	Ile64Val	**Reference (A)**<br>**Alternate (G)**	A:0.103306<br>G:0.896694	A:0.11165<br>G:0.88835	A:0.145946<br>G:0.854054	A:0.141791<br>G:0.858209	A:0.138989<br>G:0.861011	A:0.334101<br>G:0.665899
MA_116552:11911	MA_116552g0010	CVP2 LIKE 1 (CVL1)	Arg141Thr	**Reference (G)**<br>**Alternate (C)**	G:0.993534<br>C:0.00646552	G:0.978155<br>C:0.0218447	G:0.988827<br>C:0.0111732	G:0.967822<br>C:0.0321782	G:0.969203<br>C:0.0307971	G:0.969626<br>C:0.0303738
MA_10265740:3728	MA_10265740g0010	lectin protein kinase family protein	Phe60Ser	**Reference (T)**<br>**Alternate (C)**	T:0.975207<br>C:0.0247934	T:0.983254<br>C:0.0167464	T:0.931319<br>C:0.0686813	T:0.930952<br>C:0.0690476	T:0.901596<br>C:0.0984043	T:0.905405<br>C:0.0945946
MA_10265740:5430	MA_10265740g0010	lectin protein kinase family protein	Asn627Lys	**Reference (C)**<br>**Alternate (G)**	C:1<br>G:0	C:0.992424<br>G:0.00757576	C:0.980114<br>G:0.0198864	C:0.976562<br>G:0.0234375	C:0.958333<br>G:0.0416667	C:0.960094<br>G:0.0399061
MA_10265740:5624	MA_10265740g0010	lectin protein kinase family protein	Arg692His	**Reference (G)**<br>**Alternate (A)**	G:0.959184<br>A:0.0408163	G:0.966981<br>A:0.0330189	G:0.882353<br>A:0.117647	G:0.866029<br>A:0.133971	G:0.826585<br>A:0.173415	G:0.830317<br>A:0.169683
MA_10265740:5737	MA_10265740g0010	lectin protein kinase family protein	Val730Leu	**Reference (G)**<br>**Alternate (T)**	G:0.971074<br>T:0.0289256	G:0.966667<br>T:0.0333333	G:0.895161<br>T:0.104839	G:0.877404<br>T:0.122596	G:0.83659<br>T:0.16341	G:0.844595<br>T:0.155405
MA_10265740:5784	MA_10265740g0010	lectin protein kinase family protein	Gln745His	**Reference (A)**<br>**Alternate (C)**	A:0.946502<br>C:0.0534979	A:0.96875<br>C:0.03125	A:0.893443<br>C:0.106557	A:0.855392<br>C:0.144608	A:0.832423<br>C:0.167577	A:0.845622<br>C:0.154378
MA_10238024:5310	MA_10238024g0010	hypothetical protein	Glu147Lys	**Reference (G)**<br>**Alternate (A)**	G:0.960905<br>A:0.0390947	G:0.95<br>A:0.05	G:0.932796<br>A:0.0672043	G:0.908213<br>A:0.0917874	G:0.922662<br>A:0.0773381	G:0.864679<br>A:0.135321

MA_10208460:670	MA_10208460g0010	NUCLEAR RNA POLYMERASE D2B (NRPD2B)	Val31Leu	**Reference (G)**<br>**Alternate (C)**	G:0.954918<br>C:0.045082	G:0.943128<br>C:0.056872	G:0.975806<br>C:0.0241935	G:0.971292<br>C:0.0287081	G:0.97043<br>C:0.0295699	G:0.984018<br>C:0.0159817
MA_388691:5378	MA_388691g0010	Photosystem II lipoprotein (PSB27)	Cys52Tyr	**Reference (G)**<br>**Alternate (A)**	G:0.246835<br>A:0.753165	G:0.256098<br>A:0.743902	G:0.23743<br>A:0.76257	G:0.275862<br>A:0.724138	G:0.27808<br>A:0.72192	G:0.3<br>A:0.7

Table S10 Gene expression of non-DEGs (controls) in response to SHADE in Norway spruce

Gene_ID	TAIR	Primary Gene Symbol	Gene Model Description	baseMean	log2FoldChange	lfcSE	stat	pvalue	padj
MA_10021g0010	AT5G07040	RING/U-box superfamily prot	RING/U-box superfamily protein	14.570416	1.938381436	0.7522544	2.5767632	0.00997303	0.27745053
MA_10061g0010	AT2G43120	PIRIN2, PRN2	Encodes a member of the functio	177.11837	-0.177315276	0.2075426	-0.854356	0.39290785	0.99992075
MA_100380g0010	AT1G24330	ARM repeat superfamily prot	ARM repeat superfamily protein	226.52852	0.361978156	0.2558102	1.4150264	0.15706076	0.94760895
MA_100868g0010	AT2G40610	ATEXP8	member of Alpha-Expansin Gene	17.060885	1.611278446	0.7906084	2.0380233	0.0415476	0.61826162
MA_101097g0010	AT3G11750	FOLB1	Encodes an enzyme that can act a	127.67383	0.103704993	0.29544	0.3510188	0.72557425	0.99992075
MA_10048552g0010	AT1G17650	ATGLYR2	Glyoxylate reductase located in ch	410.32766	-0.29535518	0.3698652	-0.798548	0.42455241	0.99992075
MA_10055565g0010	AT2G41475	ATS3A	Embryo-specific protein 3, (ATS3)	47.664244	0.006263711	0.3115459	0.0201053	0.98395941	0.99992075
MA_10085364g0010	AT3G48730	GSAM	glutamate-1-semialdehyde 2,1-an	309.56684	0.163151046	0.1892884	0.8619177	0.38873283	0.99992075
MA_10111432g0010	AT4G34050	CCOAOMT1	Methyltransferase in the lignin bi	54.675449	0.564759944	1.1107334	0.5084568	0.61113304	0.99992075
MA_10086651g0010	AT5G59250	HP59, PLASTIDIC SUGAR TRAN	Encodes a chloroplast localized H-	146.98322	0.077330586	0.348358	0.221986	0.8243248	0.99992075

Table S11 Allele frequencies of the non-DEGs (controls) in response to SHADE in Norway spruce

				Population-wise allele frequency						
**ContigID:SnpPosition**	**Gene_ID**	**Gene name**	**Amino acid change**	**Allele**	**S1**	**S2**	**S3**	**S4**	**S5**	**S6**
MA_10021:11111	MA_10021g0010	RING/U-box supe	Gln116Glu	**Reference (C)**	0.987552	0.968293	0.991892	0.972772	0.975655	0.984018
				**Reference (G)**	0.0124481	0.0317073	0.00810811	0.0272277	0.0243446	0.0159817
MA_10021:11118	MA_10021g0010	RING/U-box supe	Ser113Ser	**Reference (C)**	0.927083	0.939614	0.959239	0.970732	0.955473	0.942922
				**Reference (A)**	0.0729167	0.0603865	0.0407609	0.0292683	0.0445269	0.0570776
MA_10021:11258	MA_10021g0010	RING/U-box supe	Thr67Ala	**Reference (A)**	0.983673	0.957346	0.991935	0.97381	0.972855	0.982063
				**Reference (G)**	0.0163265	0.042654	0.00806452	0.0261905	0.0271454	0.0179372
MA_10021:11300	MA_10021g0010	RING/U-box supe	Gly53Ser	**Reference (G)**	0.863265	0.876777	0.787634	0.644231	0.6	0.671946
				**Reference (A)**	0.136735	0.123223	0.212366	0.355769	0.4	0.328054
MA_10021:11309	MA_10021g0010	RING/U-box supe	His50Tyr	**Reference (C)**	0.944898	0.947867	0.924731	0.890476	0.923759	0.905405
				**Reference (T)**	0.055102	0.0521327	0.0752688	0.109524	0.0762411	0.0945946
MA_10061:6620	MA_10061g0010	PIRIN2, PRN2	Val34Ala	**Reference (T)**	0.343621	0.345972	0.409836	0.368421	0.283036	0.266204
				**Reference (C)**	0.656379	0.654028	0.590164	0.631579	0.716964	0.733796
MA_10061:6652	MA_10061g0010	PIRIN2, PRN2	Pro23Pro	**Reference (C)**	0.936475	0.933649	0.921622	0.911905	0.884071	0.927928
				**Reference (G)**	0.0635246	0.0663507	0.0783784	0.0880952	0.115929	0.0720721
MA_10061:6670	MA_10061g0010	PIRIN2, PRN2	Leu17Leu	**Reference (T)**	0.887295	0.889151	0.856757	0.868421	0.867908	0.855204
				**Reference (A)**	0.112705	0.110849	0.143243	0.131579	0.132092	0.144796
MA_100380:7172	MA_100380g0010	ARM repeat supe	Val159Ile	**Reference (G)**	0.989451	0.980392	0.97541	1	0.999094	1
				**Reference (A)**	0.0105485	0.0196078	0.0245902	0	0.0009058	0
MA_100380:7185	MA_100380g0010	ARM repeat supe	Thr154Thr	**Reference (T)**	0.925847	0.948529	0.925824	0.878788	0.894737	0.857143
				**Reference (G)**	0.0741525	0.0514706	0.0741758	0.121212	0.105263	0.142857
MA_100380:7212	MA_100380g0010	ARM repeat supe	His145Gln	**Reference (T)**	0.783843	0.7725	0.798343	0.870558	0.864545	0.892523
				**Reference (G)**	0.216157	0.2275	0.201657	0.129442	0.135455	0.107477
MA_100868:5630	MA_100868g0010	ATEXP8	Glu104Lys	**Reference (G)**	0.910042	0.903382	0.895161	0.888614	0.933996	0.917431
				**Reference (A)**	0.0899582	0.0966184	0.104839	0.111386	0.0660036	0.0825688
MA_100868:5663	MA_100868g0010	ATEXP8	Ser93Cys	**Reference (A)**	0.954936	0.956311	0.940217	0.972772	0.971296	0.955399
				**Reference (T)**	0.0450644	0.0436893	0.0597826	0.0272277	0.0287037	0.0446009
MA_101097:19803	MA_101097g0010	FOLB1	Ser17Asn	**Reference (G)**	0.944215	0.971564	0.954054	0.978261	0.983186	0.984091
				**Reference (A)**	0.0557851	0.028436	0.0459459	0.0217391	0.0168142	0.0159091
MA_101097:19843	MA_101097g0010	FOLB1	Gly4Arg	**Reference (G)**	0.921488	0.954762	0.967391	0.97619	0.969697	0.961538
				**Reference (A)**	0.0785124	0.0452381	0.0326087	0.0238095	0.030303	0.0384615
MA_10048552:1277	MA_10048552g0010	ATGLYR2	Gly6Val	**Reference (G)**	0.975309	0.992925	0.986559	0.988038	0.975439	0.986486
				**Reference (T)**	0.0246914	0.00707547	0.0134409	0.0119617	0.0245614	0.0135135
MA_10048552:1293	MA_10048552g0010	ATGLYR2	Gly11Gly	**Reference (C)**	0.362705	0.375	0.400538	0.419431	0.490317	0.412162
				**Reference (A)**	0.637295	0.625	0.599462	0.580569	0.509683	0.587838
MA_10055565:3050	MA_10055565g0010	ATS3A	Asp21Asp	**Reference (C)**	0.969828	0.965517	0.983425	0.971939	0.982143	0.961905
				**Reference (T)**	0.0301724	0.0344828	0.0165746	0.0280612	0.0178571	0.0380952
MA_10055565:3055	MA_10055565g0010	ATS3A	Ala20Thr	**Reference (G)**	0.980851	0.985222	0.97486	0.94359	0.954545	0.943128
				**Reference (A)**	0.0191489	0.0147783	0.0251397	0.0564103	0.0454545	0.056872
MA_10085364:3481	MA_10085364g0010	GSAM	Glu211Glu	**Reference (A)**	0.863043	0.893564	0.840782	0.914508	0.925414	0.908451
				**Reference (G)**	0.136957	0.106436	0.159218	0.0854922	0.0745856	0.0915493
MA_10085364:3493	MA_10085364g0010	GSAM	Gly207Gly	**Reference (A)**	0.978992	0.985646	0.983607	0.972222	0.979167	0.972222
				**Reference (T)**	0.0210084	0.0143541	0.0163934	0.0277778	0.0208333	0.0277778
MA_10085364:3886	MA_10085364g0010	GSAM	Ala76Ala	**Reference (A)**	0.973361	0.980952	0.981183	0.962085	0.976357	0.961712
				**Reference (T)**	0.0266393	0.0190476	0.0188172	0.0379147	0.0236427	0.0382883
MA_10085364:3907	MA_10085364g0010	GSAM	Asp69Glu	**Reference (T)**	0.985656	0.995238	0.983871	0.988152	0.987741	0.997758
				**Reference (A)**	0.0143443	0.0047619	0.016129	0.0118483	0.0122592	0.00224215
MA_10085364:3926	MA_10085364g0010	GSAM	Glu63Gly	**Reference (A)**	0.985714	0.995238	0.983871	0.988152	0.988616	0.997748
				**Reference (G)**	0.0142857	0.0047619	0.016129	0.0118483	0.0113835	0.00225225
MA_10111432:302	MA_10111432g0010	CCOAOMT1	Val53Asp	**Reference (T)**	0.983607	0.969194	0.978495	0.938095	0.920635	0.959459
				**Reference (A)**	0.0163934	0.0308057	0.0215054	0.0619048	0.0793651	0.0405405
MA_10111432:322	MA_10111432g0010	CCOAOMT1	Arg60Arg	**Reference (A)**	0.967078	0.936019	0.886486	0.858173	0.794484	0.790541
				**Reference (C)**	0.0329218	0.063981	0.113514	0.141827	0.205516	0.209459
MA_10111432:336	MA_10111432g0010	CCOAOMT1	Glu64Asp	**Reference (G)**	0.843621	0.851415	0.857527	0.787081	0.740675	0.791855
				**Reference (T)**	0.156379	0.148585	0.142473	0.212919	0.259325	0.208145
MA_10111432:340	MA_10111432g0010	CCOAOMT1	Ala66Thr	**Reference (G)**	0.738683	0.787736	0.798387	0.695238	0.633628	0.710407
				**Reference (A)**	0.261317	0.212264	0.201613	0.304762	0.366372	0.289593
MA_10086651:5150	MA_10086651g0010	HP59, PLASTIDIC	Pro41Pro	**Reference (A)**	0.945607	0.939904	0.928962	0.908213	0.956912	0.961009
				**Reference (G)**	0.0543933	0.0600962	0.0710383	0.0917874	0.043088	0.0389908
MA_10086651:5166	MA_10086651g0010	HP59, PLASTIDIC	Trp36Ser	**Reference (G)**	0.943983	0.940476	0.92973	0.903846	0.952509	0.961187
				**Reference (C)**	0.0560166	0.0595238	0.0702703	0.0961538	0.047491	0.0388128

Fig.S1 Developmental stage of the Norway spruce seedlings with fully developed hypocotyls when the seedlings were harvested for all the experiments included in the study.

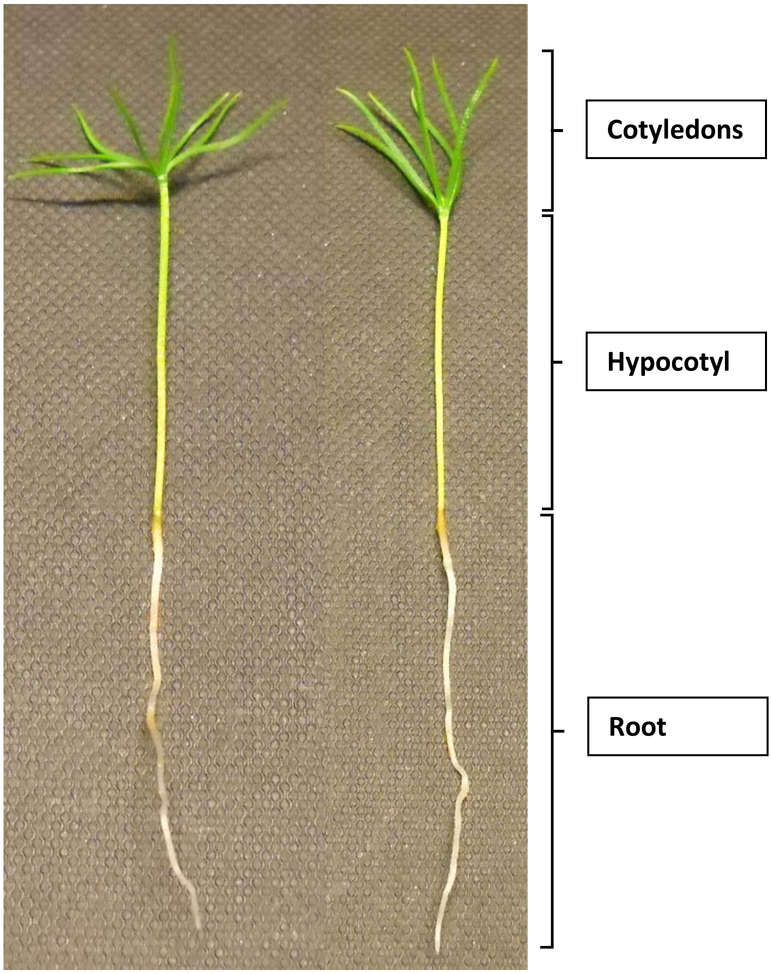

Fig.S2 Metabolism overview with the DEGs in response to shade in the northern and southern populations of Norway spruce. The network map represents the gene regulation in the northern population as compared to the southern one

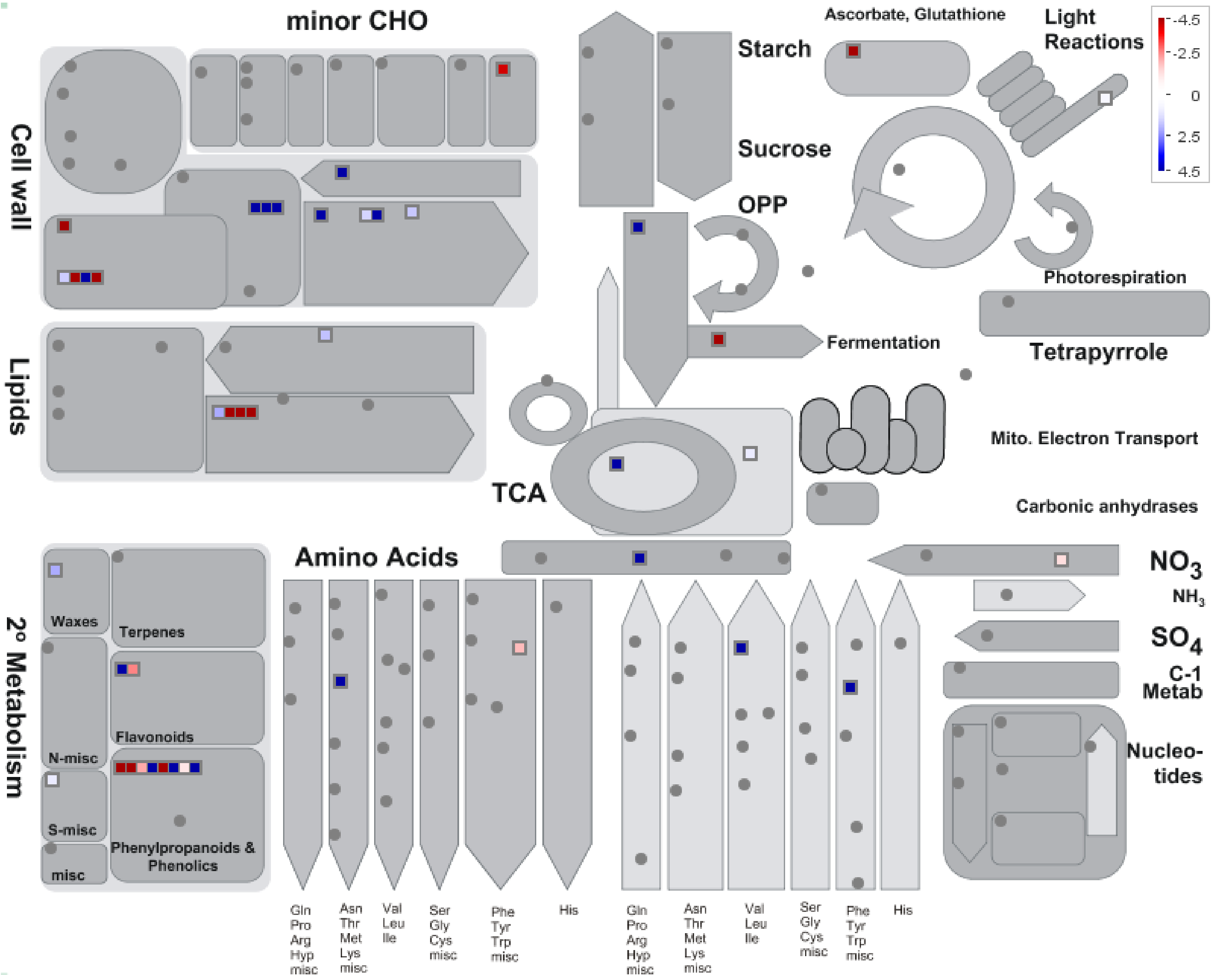

Fig.S3 Secondary metabolism with the DEGs in response to shade in the northern and southern populations of Norway spruce. The network map represents the gene regulation in the northern population as compared to the southern one

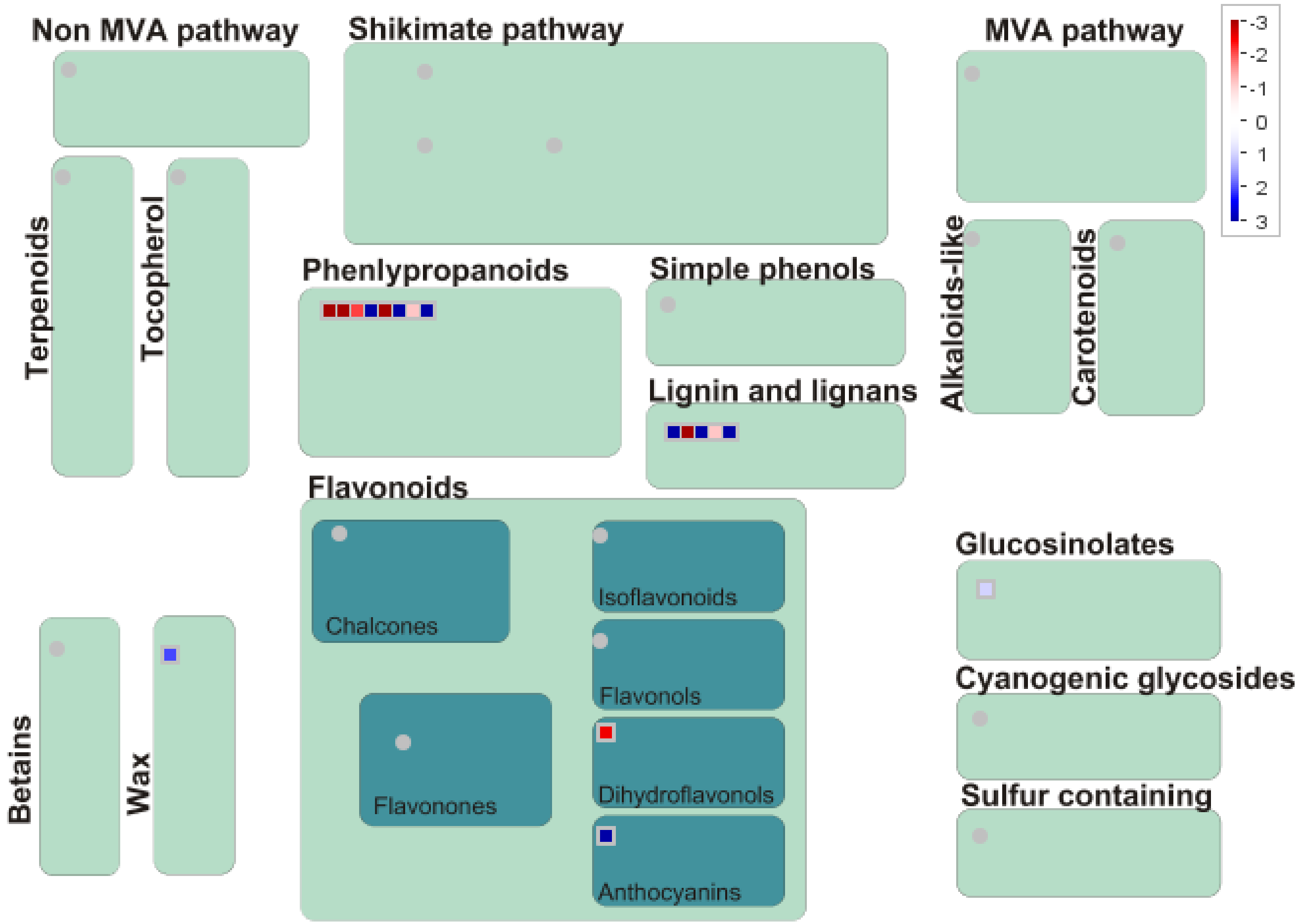

Fig.S4 Phenylpropanoid pathway with the DEGs in response to shade in the northern and southern populations of Norway spruce. The network map represents the gene regulation in the northern population as compared to the southern one

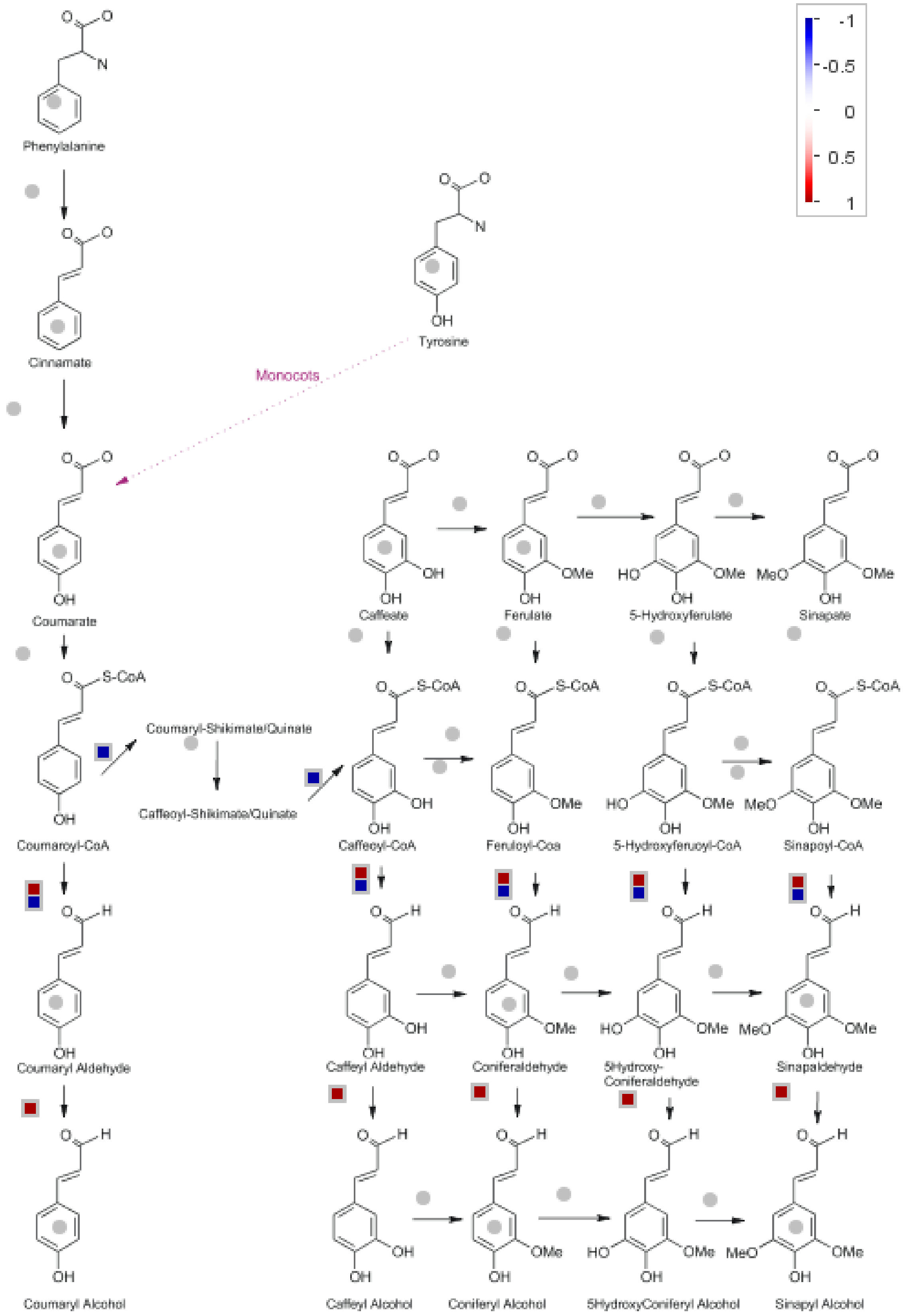

Fig.S5 Regulation overview with the DEGs in response to shade in the northern and southern populations of Norway spruce. The network map represents the gene regulation in the northern population as compared to the southern one

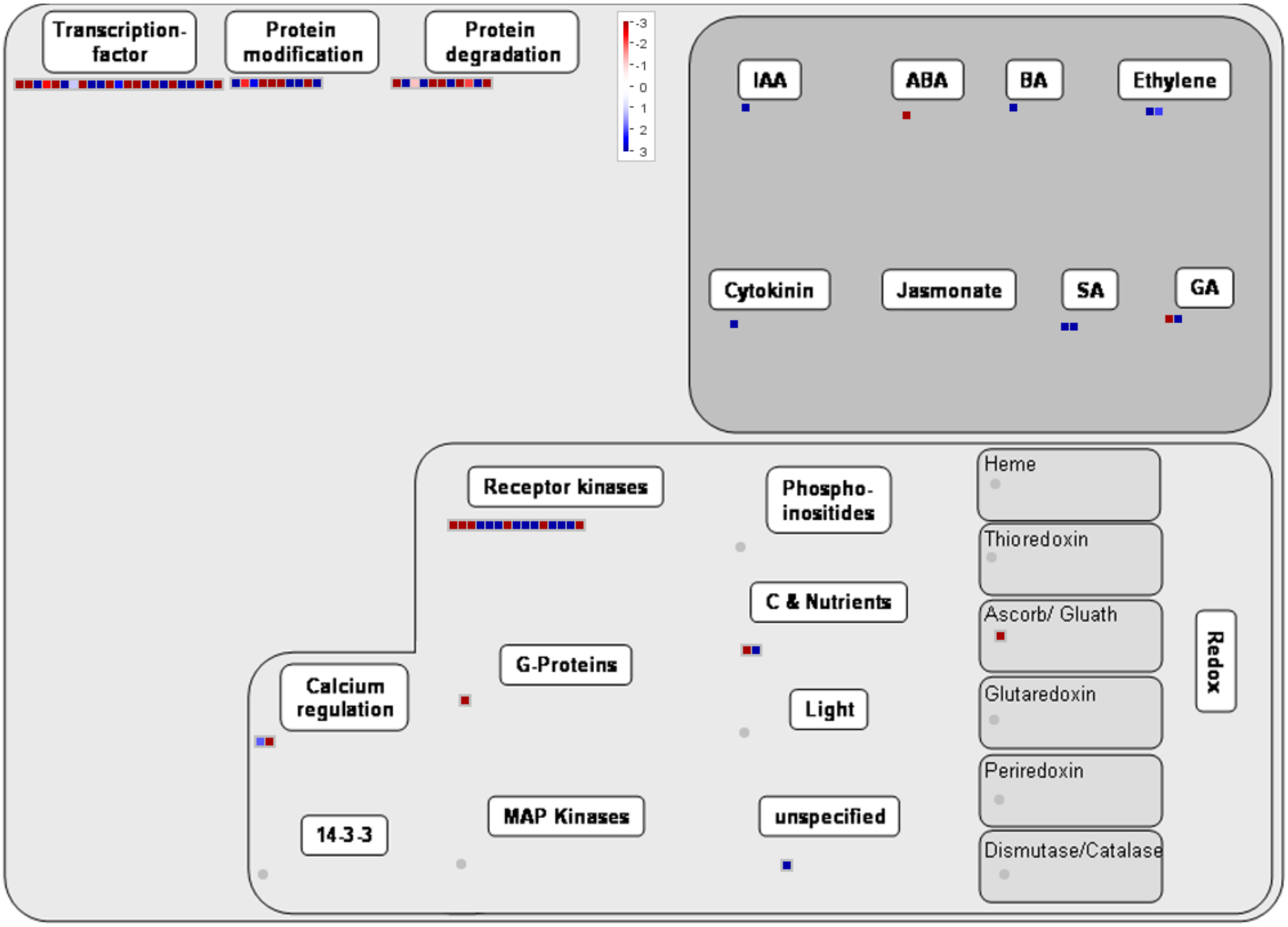

Fig.S6 Cellular response overview with the DEGs in response to shade in the northern and southern populations of Norway spruce. The network map represents the gene regulation in the northern population as compared to the southern one

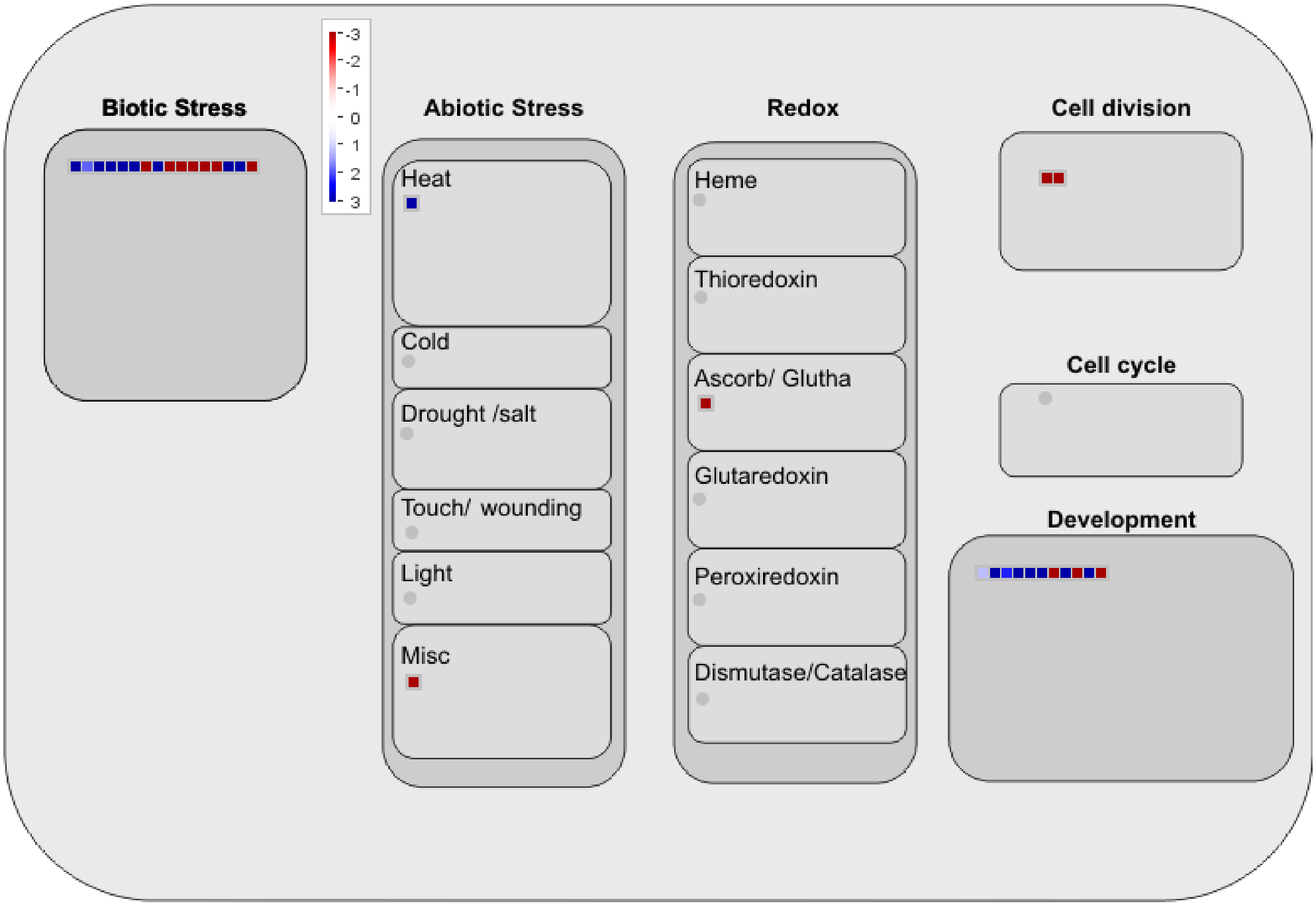

Fig.S7 Cline with reference to variation in allele and genotype frequencies of SNPs in the *LOV1* gene in Norway spruce populations across Sweden

a and c: allele frequencies of R156K and D190N respectively

b and d: genotype frequencies of R156K and D190N respectively, Tukey's *post-hoc* categorization is indicated above the bars

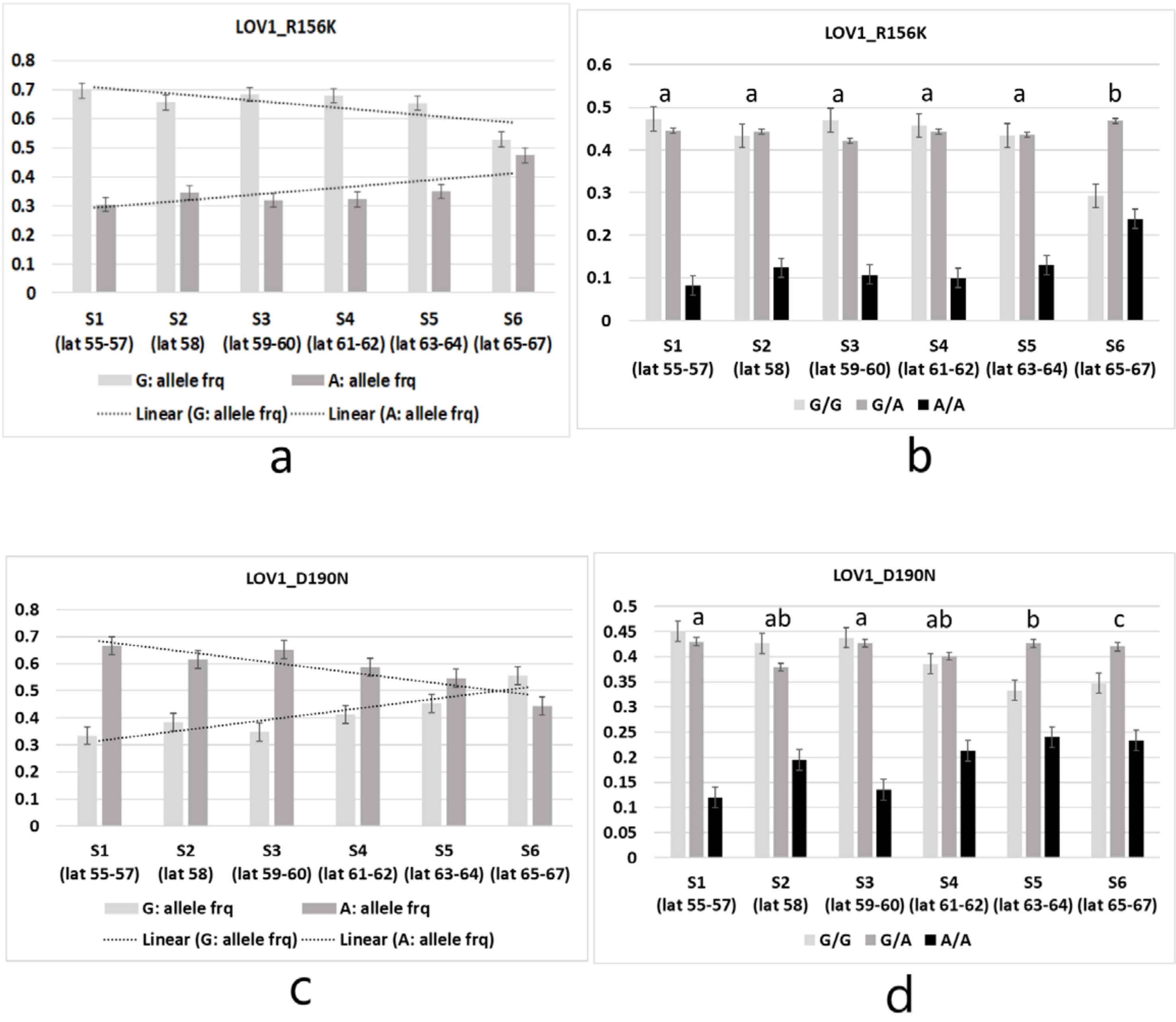

Fig.S8 Cline with reference to variation in allele and genotype frequencies of SNPs in the *SCRM2* gene in Norway spruce populations across Sweden

a and c: allele frequencies of T208M and S238N respectively

b and d: genotype frequencies of T208M and S238N respectively, Tukey's *post-hoc* categorization is indicated above the bars

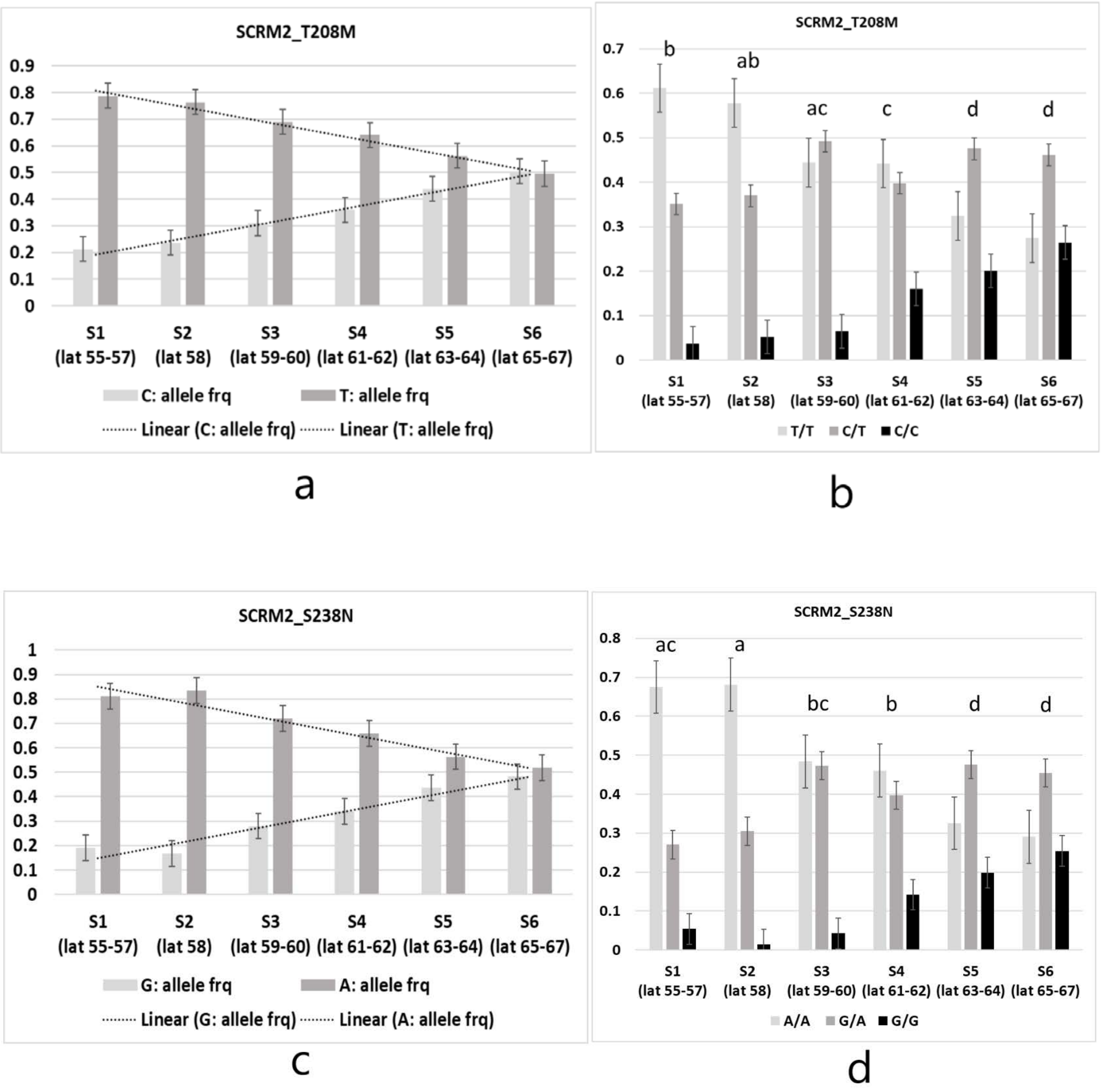

Fig.S9 Cline with reference to variation in allele and genotype frequencies of SNPs in the *TCP2* gene in Norway spruce populations across Sweden

a c e g and i: allele frequencies of F4S, V25A, E32G, E51K and I53F respectively

b d f h and j: genotype frequencies of F4S, V25A, E32G, E51K and I53F respectively, Tukey's *post-hoc* categorization is indicated above the bars

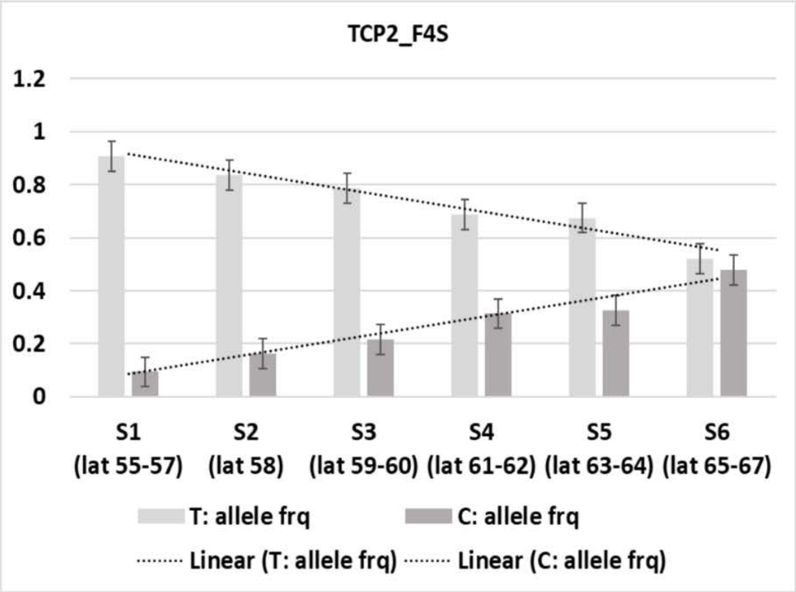

a

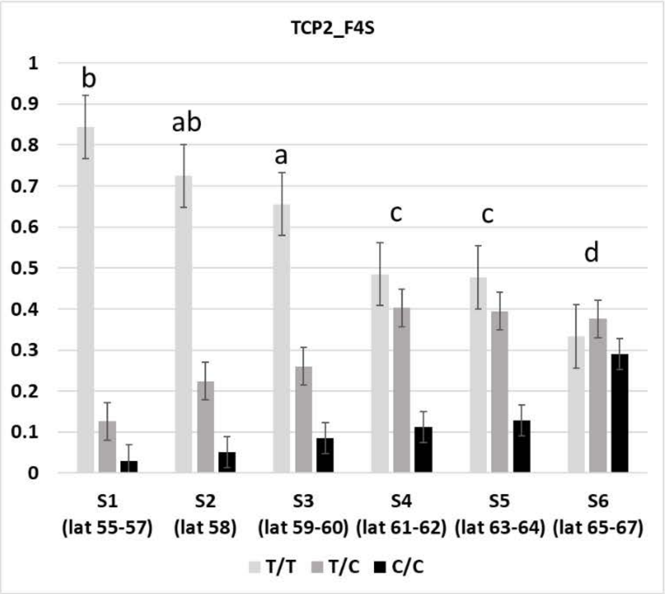

b

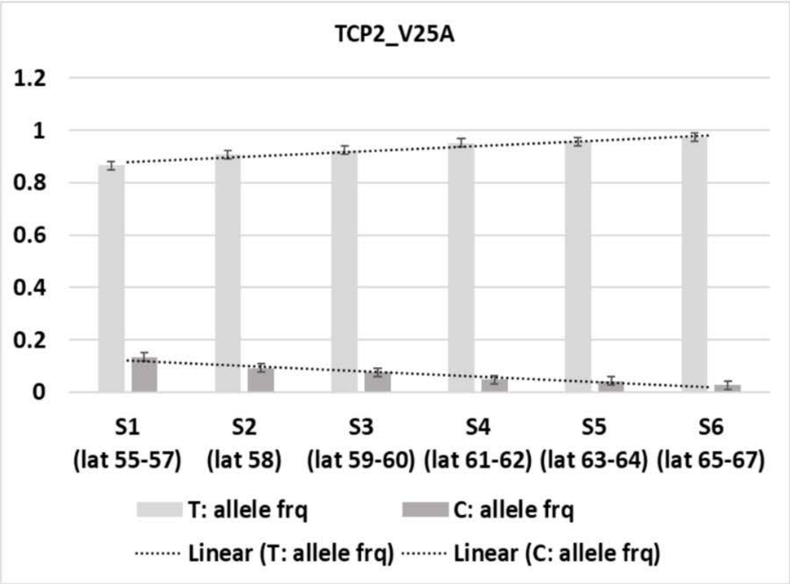

c

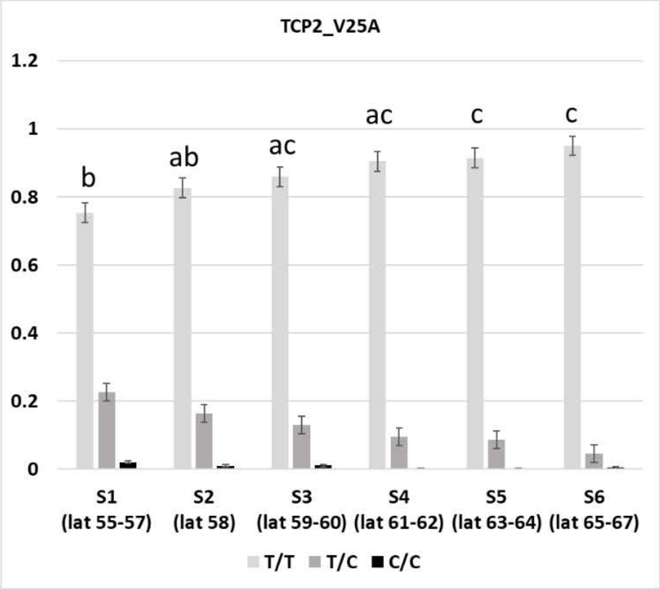

d

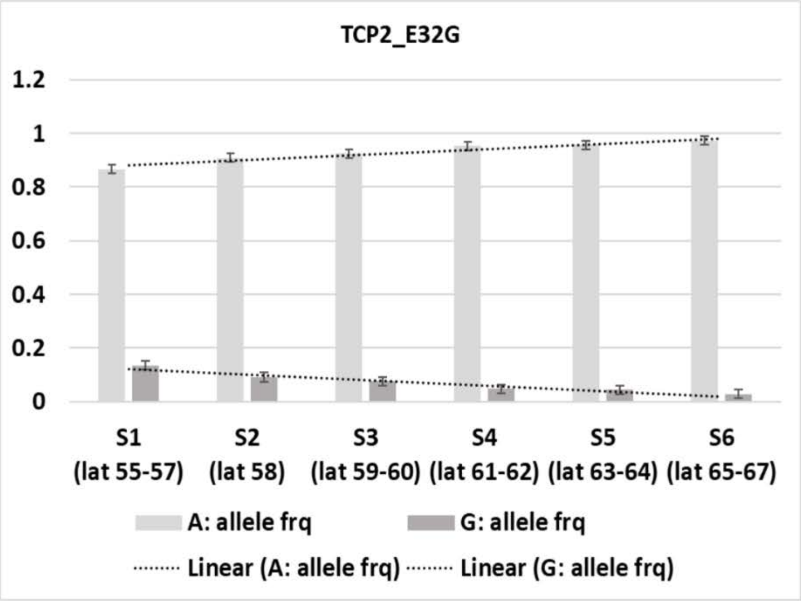

TCP2_E32G
1.2
1
0.8
0.6
0.4
0.2
0
S1 (lat 55-57)
S2 (lat 58)
S3 (lat 59-60)
S4 (lat 61-62)
S5 (lat 63-64)
S6 (lat 65-67)
A: allele frq
G: allele frq
Linear (A: allele frq)
Linear (G: allele frq)

e

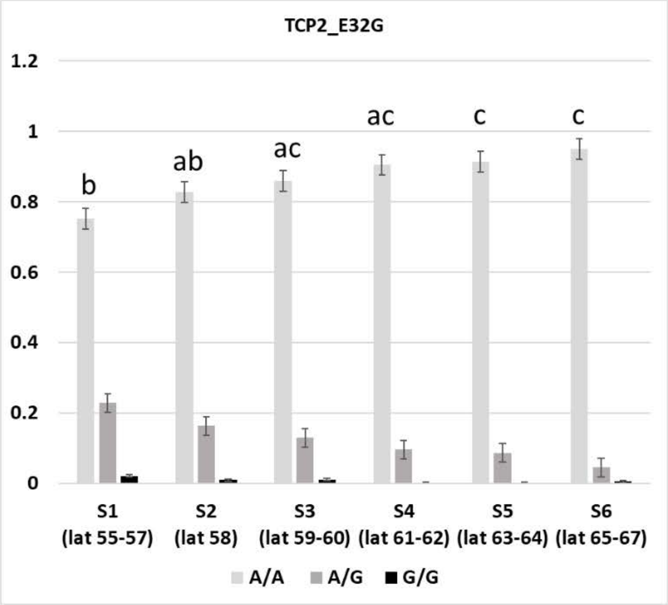

TCP2_E32G
1.2
1
0.8
0.6
0.4
0.2
0
b
ab
ac
ac
c
c
S1 (lat 55-57)
S2 (lat 58)
S3 (lat 59-60)
S4 (lat 61-62)
S5 (lat 63-64)
S6 (lat 65-67)
A/A
A/G
G/G

f

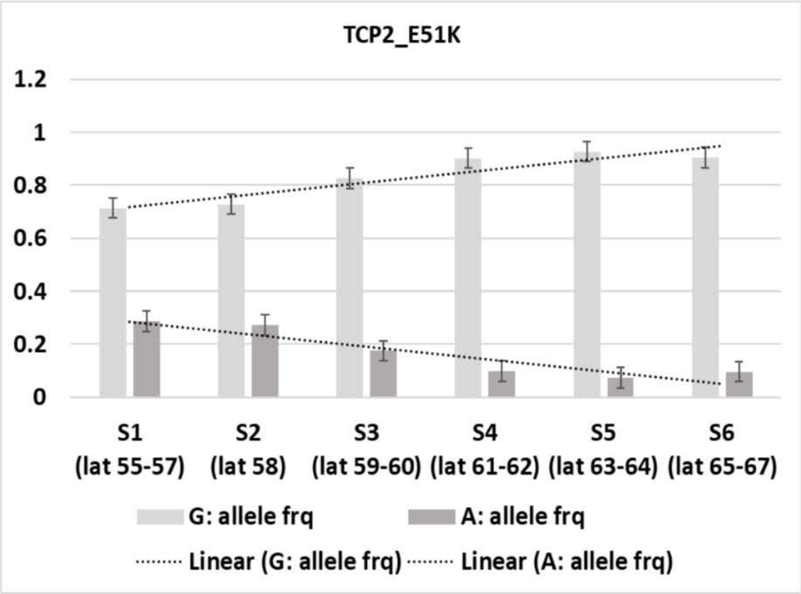

TCP2_E51K
1.2
1
0.8
0.6
0.4
0.2
0
S1 (lat 55-57)
S2 (lat 58)
S3 (lat 59-60)
S4 (lat 61-62)
S5 (lat 63-64)
S6 (lat 65-67)
G: allele frq
A: allele frq
Linear (G: allele frq)
Linear (A: allele frq)

g

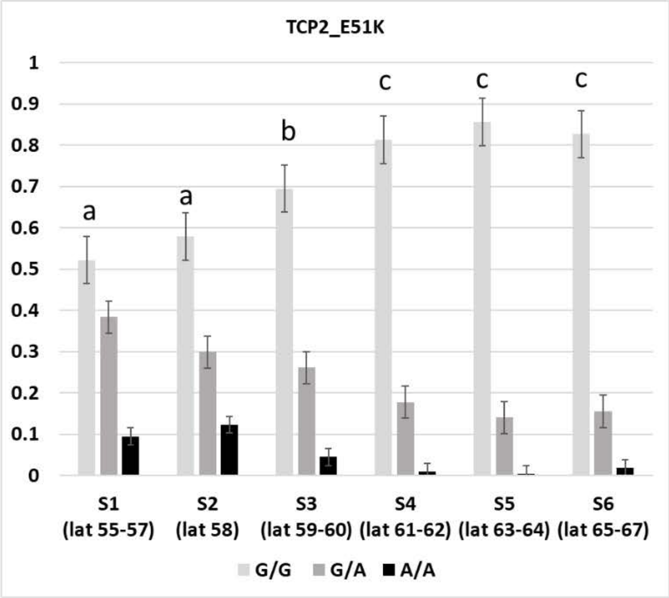

TCP2_E51K
1
0.9
0.8
0.7
0.6
0.5
0.4
0.3
0.2
0.1
0
a
a
b
c
c
c
S1 (lat 55-57)
S2 (lat 58)
S3 (lat 59-60)
S4 (lat 61-62)
S5 (lat 63-64)
S6 (lat 65-67)
G/G
G/A
A/A

h

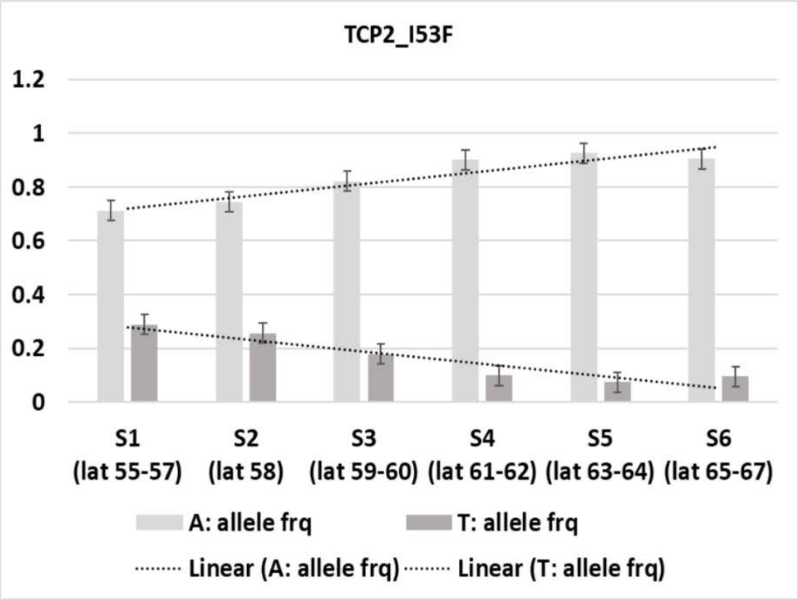
TCP2_I53F
1.2
1
0.8
0.6
0.4
0.2
0
S1 (lat 55-57)
S2 (lat 58)
S3 (lat 59-60)
S4 (lat 61-62)
S5 (lat 63-64)
S6 (lat 65-67)
A: allele frq
T: allele frq
Linear (A: allele frq)
Linear (T: allele frq)

i

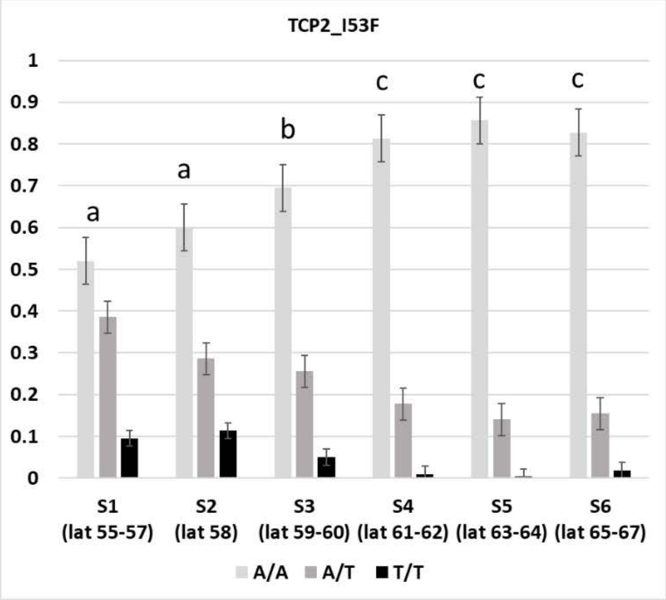
TCP2_I53F
1
0.9
0.8
0.7
0.6
0.5
0.4
0.3
0.2
0.1
0
a
a
b
c
c
c
S1 (lat 55-57)
S2 (lat 58)
S3 (lat 59-60)
S4 (lat 61-62)
S5 (lat 63-64)
S6 (lat 65-67)
A/A
A/T
T/T

j

Fig.S10 Cline with reference to variation in allele and genotype frequencies of SNPs in the *NAC036* gene in Norway spruce populations across Sweden

a and c: allele frequencies of D206E and T237M respectively

b and d: genotype frequencies of D206E and T237M respectively, Tukey's *post-hoc* categorization is indicated above the bars

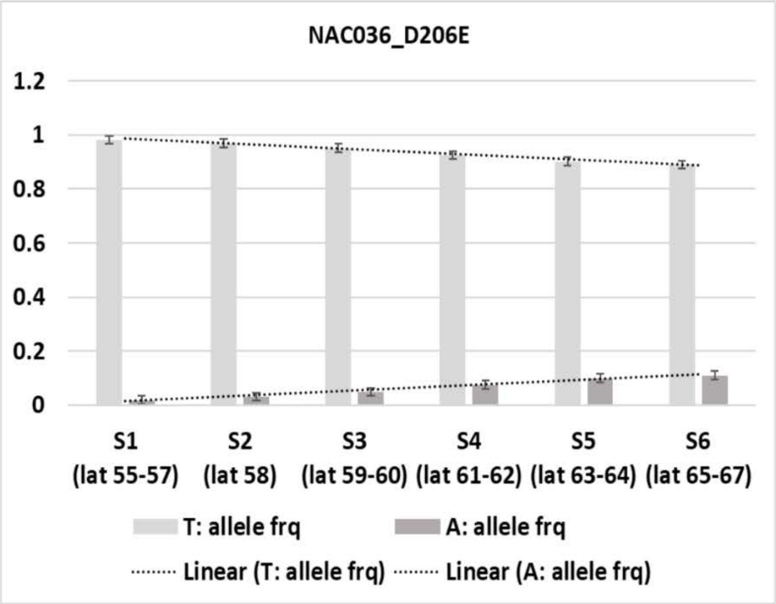

a

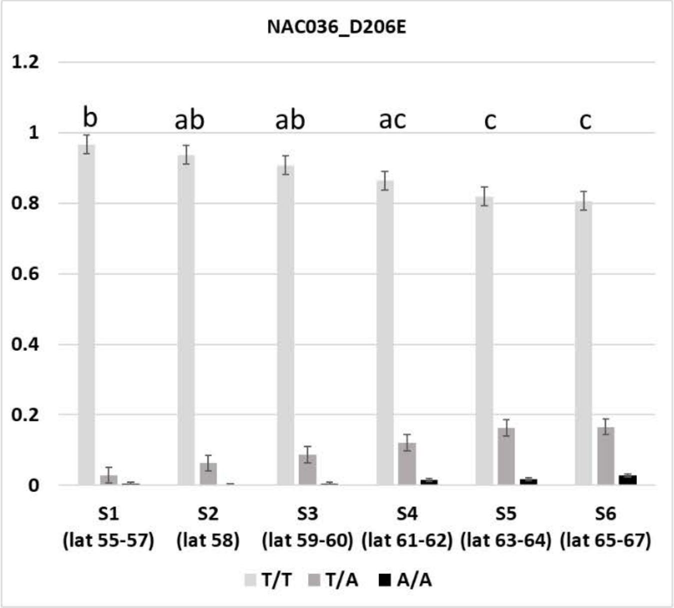

b

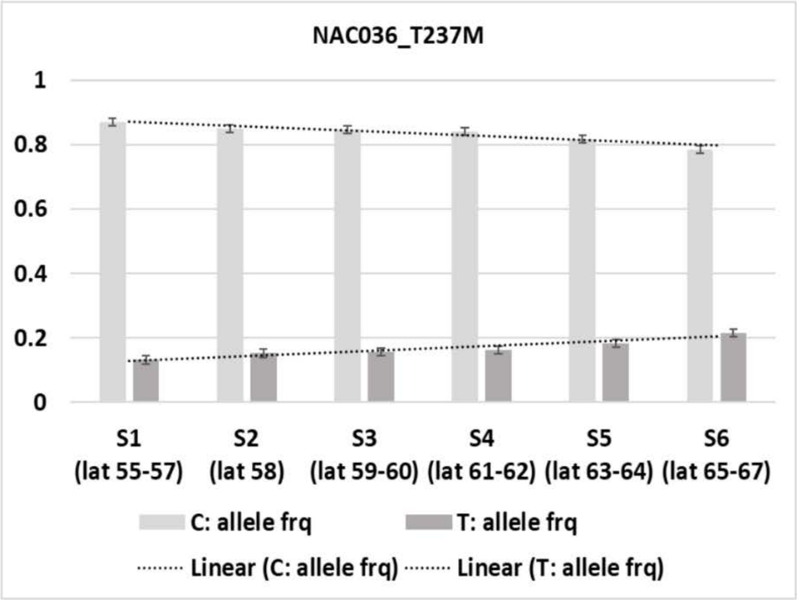

c

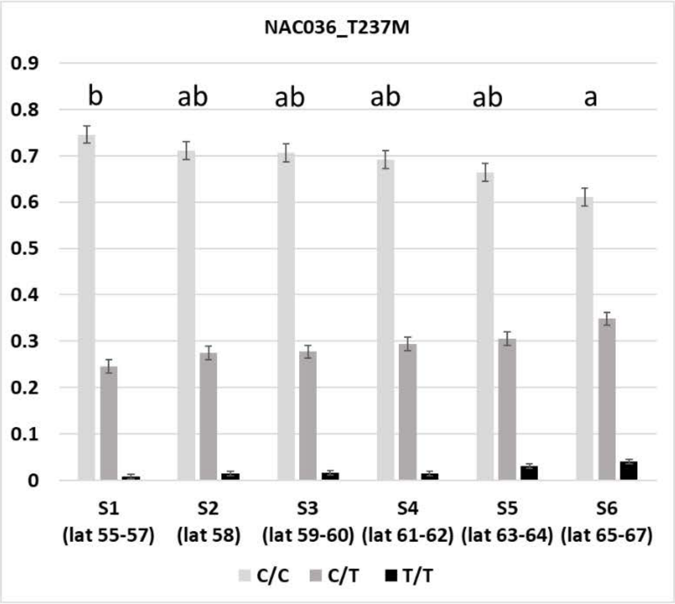

d

Fig.S11 Cline with reference to variation in allele and genotype frequencies of SNPs in the *EXPB3* gene in Norway spruce populations across Sweden

a c and e: allele frequencies of K3R, R6S and C32R respectively

b d and f: genotype frequencies of K3R, R6S and C32R respectively, Tukey's *post-hoc* categorization is indicated above the bars

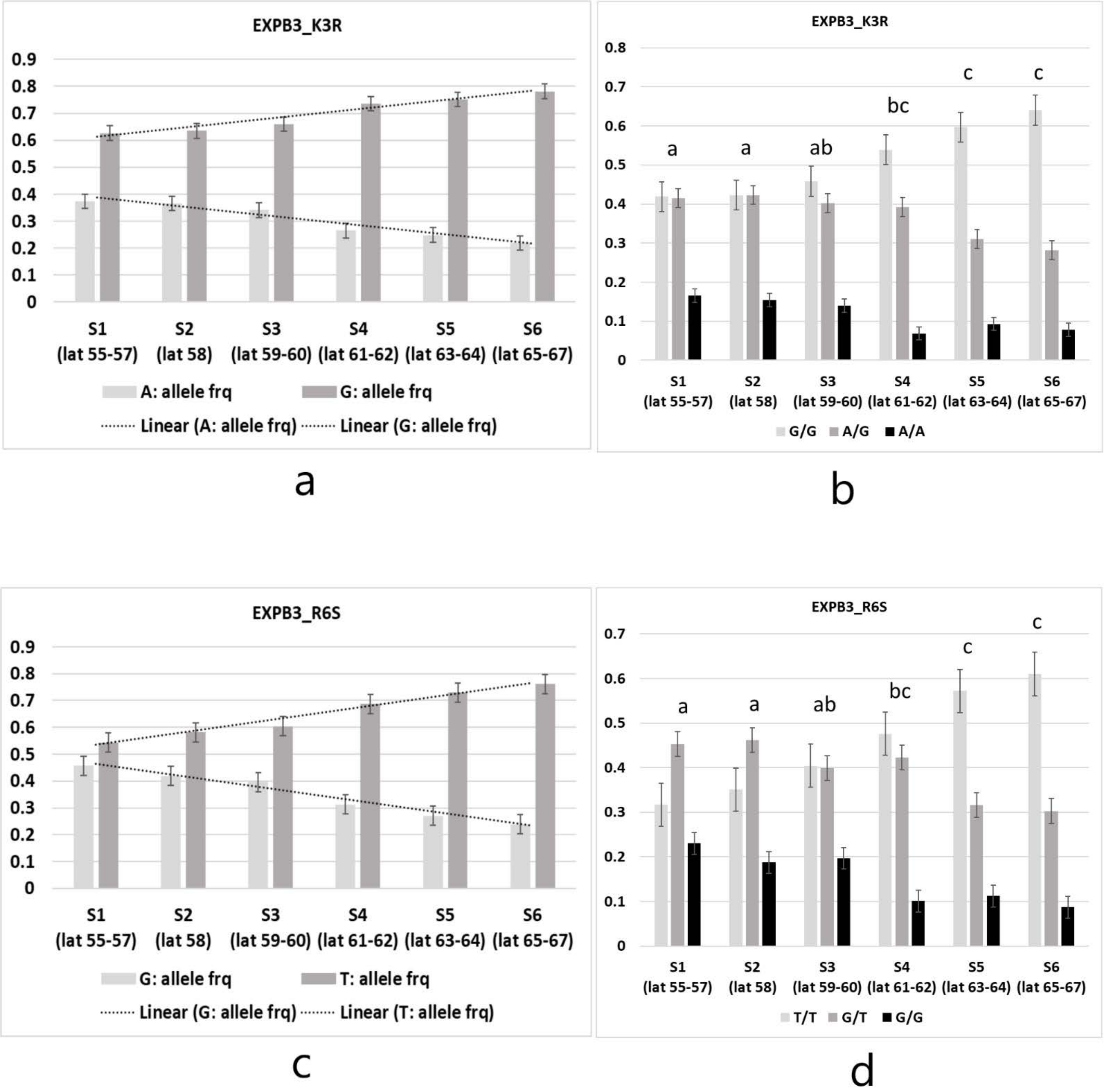

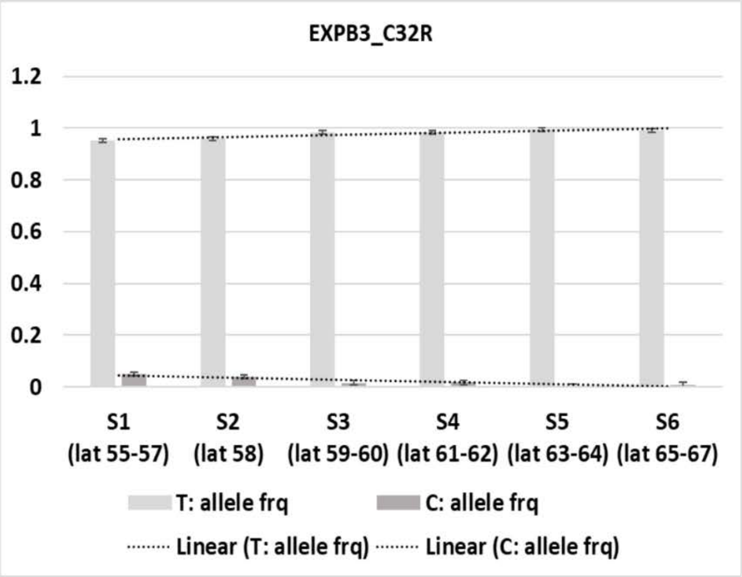
EXPB3_C32R
1.2
1
0.8
0.6
0.4
0.2
0
S1 (lat 55-57)
S2 (lat 58)
S3 (lat 59-60)
S4 (lat 61-62)
S5 (lat 63-64)
S6 (lat 65-67)
T: allele frq
C: allele frq
Linear (T: allele frq)
Linear (C: allele frq)

e

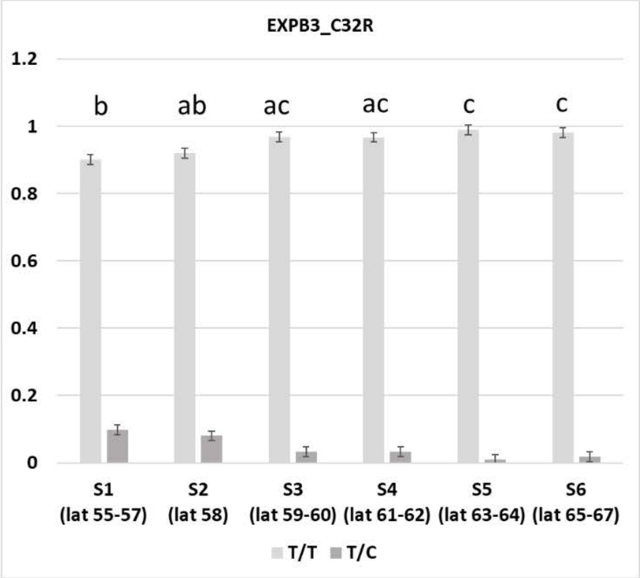
EXPB3_C32R
1.2
1
0.8
0.6
0.4
0.2
0
b
ab
ac
ac
c
c
S1 (lat 55-57)
S2 (lat 58)
S3 (lat 59-60)
S4 (lat 61-62)
S5 (lat 63-64)
S6 (lat 65-67)
T/T
T/C

f

Fig.S12 Cline with reference to variation in allele and genotype frequencies of SNP in the *FLZ6* gene in Norway spruce populations across Sweden

a allele frequencies of L88V

b genotype frequencies of L88V, Tukey's *post-hoc* categorization is indicated above the bars

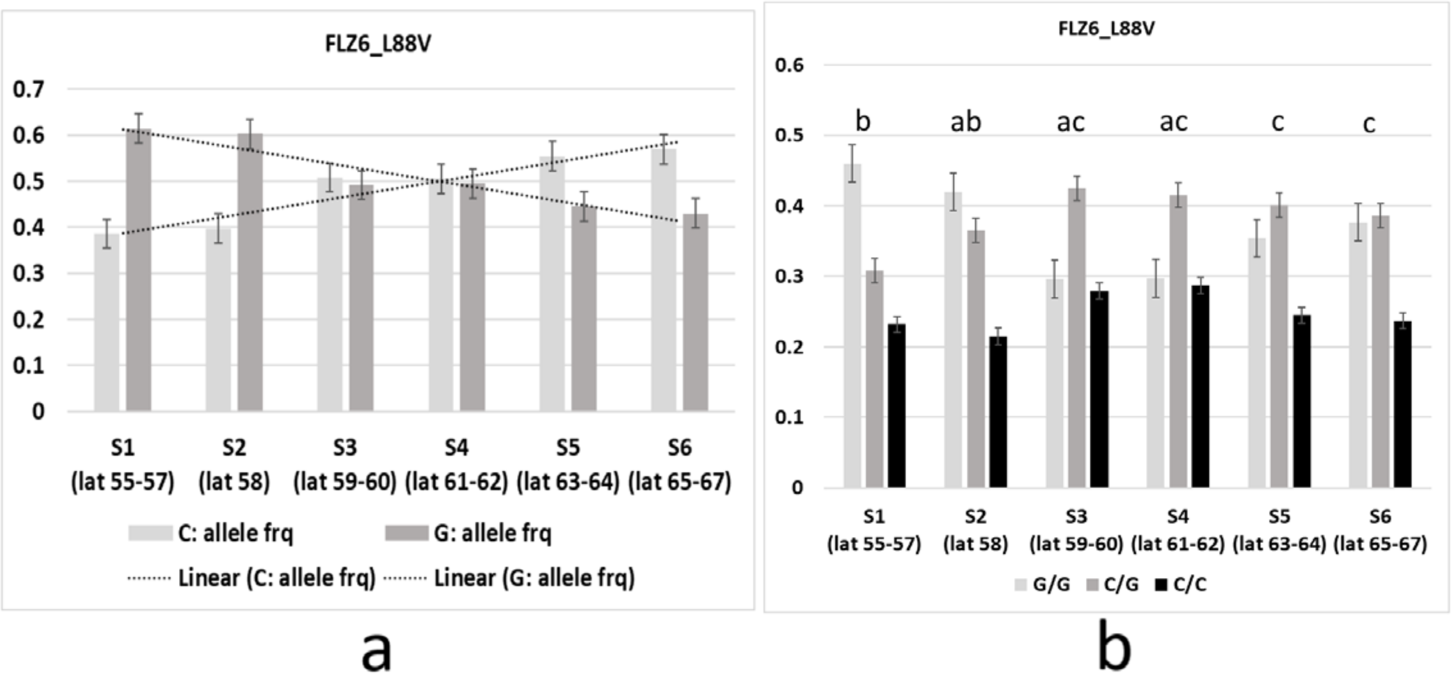

Fig.S13 Cline with reference to variation in allele and genotype frequencies of SNPs in the *VRLK1* gene in Norway spruce populations across Sweden

- **a and c:** allele frequencies of A265V and V283L respectively
- **b and d:** genotype frequencies of A265V and V283L respectively, Tukey's *post-hoc* categorization is indicated above the bars

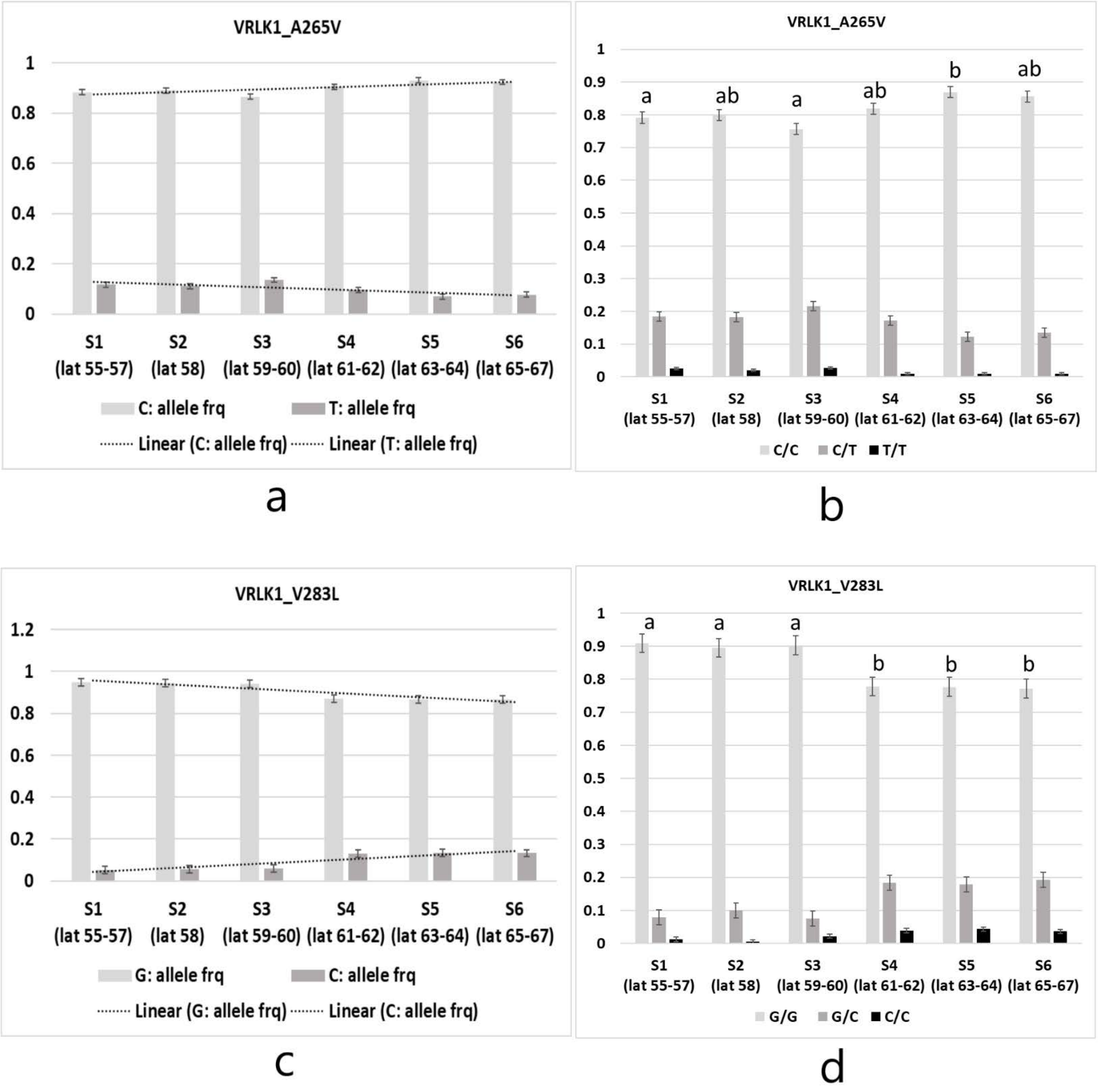

Fig.S14 Cline with reference to variation in allele and genotype frequencies of SNPs in the *RPS2* gene in Norway spruce populations across Sweden

a and c: allele frequencies of S123N and E166A respectively

b and d: genotype frequencies of S123N and E166A respectively, Tukey's *post-hoc* categorization is indicated above the bars

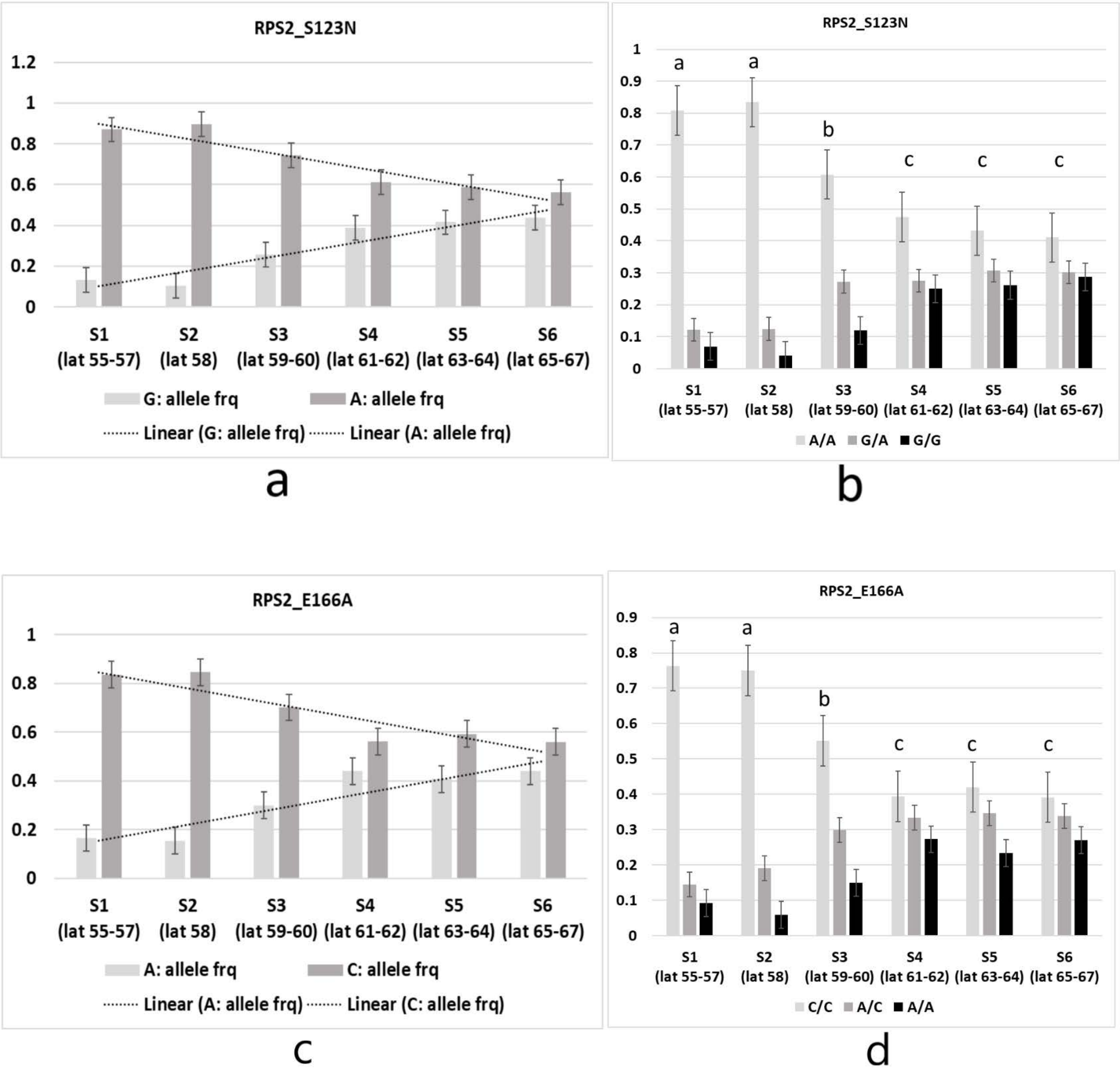

Fig.S15 Domains of TEOSINTE BRANCHED 1, CYCLOIDEA AND PCF TRANSCRIPTION FACTOR 2 (TCP2)
Missense polymorphism sites are highlighted in green in the Norway spruce gene sequences
Reference and alternative amino acids are displayed in dotted box
Well characterized domains of the genes are highlighted in other colors in the *Arabidopsis thaliana* gene sequences
MA_92659g0010 [551 residues] Norway spruce (http://congenie.org/)
AT4G18390 [365 residues] *Arabidopsis thaliana* (https://www.arabidopsis.org)

```
CLUSTAL O(1.2.4) multiple sequence alignment
                        F or S                  V or A   E or G              E or K   I or F
MA_92659p0010      MDSFEEDSDYLEEDNDHFGFNENAVPTEAKDENCKALYEGDLFSSIKPHSEYISVEQERK      60
AT4G18390          ------------------------------------------------------------      0


MA_92659p0010      YGHQGPTAYSYACCCYSSPFSVGHETTGCKKPTNDPAAYPLNGSHKASIADSSPRHMPPG      120
AT4G18390          ------------------------------------------------------------      0


MA_92659p0010      RPCSLIAQHIDPNPCEDEVPVNLMSDMFQFPDFLETYAQSAEWEKKNTLQRKQENNPKRS      180
AT4G18390          ----------------------MIGDLM------KNNNNG-------DVV-DNEVNNRLS      24
                                         ::.*::      :.  :.        :  .:* * : *

MA_92659p0010      RLQFQQPEKSSGNMNSSGFMEPQKIRRHSGNSDRHRKVVTSRGPRDRRMRLSIETAIKFF      240
AT4G18390          RW----------HHN---SSRIIRVSRASGGKDRHSKVLTSKGPRDRRVRLSVSTALQFY      71
                   *           : *     .  :: * **..*** **:**:******:***:.**::*:
42-100: TCP domain: Probably involved in DNA-binding and protein-protein interactions

MA_92659p0010      EIQDRLGFDQPSKAVEWLIQNSRSAIDQLPAISQSALDISSHLKSNSDSLSPFLHRDTDI      300
AT4G18390          DLQDRLGYDQPSKAVEWLIKAAEDSISELPSLNNTHFPTDDENHQNQT-LTTVA------      124
                   ::*****:***********: :...:*.:**::.:: :  ... :.*.   *: .

MA_92659p0010      SGADFVTGFEENMGIADFLSPSLFTEPSILKEQSRSKRKKYNSRKNSVKSALVNSTGRRR      360
AT4G18390          ---------------ANSLSKS---------------------------ACSSNSDTSK      141
                                  *: ** *                            *  ..:.  :

MA_92659p0010      HIAGLKENRS----KARERARERTKEKSCARVKATS-PSPHMNHTRAPEIKALSFQENTA      415
AT4G18390          NSSGLSLSRSELRDKARERARERTAKETKERDHNHTSFTDLLNSGSDPVNSNRQWMASAP      201
                   : :**. .**    ********** :::  * :  :  :  :*    *   .  .:  .:
                         151-172: R domain: May mediate protein-protein interactions

MA_92659p0010      S------ASSGNTLNDRMEASISQLYCVAPTLKIGVQEKSLNRRFHHHGCSLTAQDSVKE      469
AT4G18390          SSSPMEYFSSGLILGSGQQTHFPISTNSHPF-SSISDHHHHHPHHQHQEFSFVPDHLI--      258
                   *       ***  *..  :: :        *  .   :.:  : :.:*:  *:. :. :

MA_92659p0010      LAENSHLSGHSNLSFNYSQSQIQSSVQTVAQRYKHQPEDRSMESTGSFTGLCSTTFPPAV      529
AT4G18390          -SPAESNGGAFNLDFNMSTPSGAG------------AAVSAASGGGFSGFNRGTLQSNS      304
                    :  .  .*  **.** *   .  .                *  * *.*:*:   *:

MA_92659p0010      ------------------------------PAMDVDLQSRISDLYVDYSLLQ--------      551
AT4G18390          TNQHQSFLANLQRFPTSESGGGPQFLFGALPAENHHHN-HQFQLYYENGCRNSSEHKGKG      363
                                                 ** : . : :  :** : .  :

MA_92659p0010      --      551
AT4G18390          KN      365
```

Fig.S16 Domains of LONG VEGETATIVE PHASE 1 (LOV1)

Missense polymorphism sites are highlighted in green in the Norway spruce gene sequences

Reference and alternative amino acids are displayed in dotted box

Well characterized domains of the genes are highlighted in other colors in the *Arabidopsis thaliana* gene sequences

MA_16619g0010 [288 residues] Norway spruce (http://congenie.org/)

AT2G02450 [414 residues] *Arabidopsis thaliana* (https://www.arabidopsis.org)

```
CLUSTAL O(1.2.4) multiple sequence alignment


MA_16619p0010       -------------------------------------------------MDLPGFRFYPT      11
AT2G02450           MAIVSSTTSIIPMSNQVNNNEKGIEDNDHRGGQESHVQNEDEADDHDHDMVMPGFRFHPT      60
                                                                     * :*****:**

MA_16619p0010       EEELVGFYLKKRIQGSHPMNFDIVIPTLDLYRYDPCELPSLAHDVEERQWFFFVPRDHKN      71
AT2G02450           EEELIEFYLRRKVEGKRFN--VELITFLDLYRYDPWELPAMAA-IGEKEWYFYVPRDRKY      117
                    ****: ***:::::*.:      :*  ******** ***::*  : *::*:*:****:*

MA_16619p0010       SS-GRPNRLTKSGYWKATGSDRAIRNELLQCIGLKKILVFYKGKAPYGQKTDWIMNEYRL      130
AT2G02450           RNGDRPNRVTTSGYWKATGADRMIRSETSRPIGLKKTLVFYSGKAPKGTRTSWIMNEYRL      177
                     . .****:*.********:** **.*  : ***** ****.**** * :*.********
                           51-198: NAC domain: DNA-binding and dimerization domain
                                                      R or K
MA_16619p0010       PDLRSSSVKVRDIVLCRIYRKAASQRSMELQAKPD--HVVKEE-----TAVSGDEYEDTS      183
AT2G02450           PHHETEKYQKAEISLCRVYKRPGVEDHPSVPRSLSTRHHNHNSSTSSRLALRQQQHHSSS      237
                    *. .:.. :  :* ***:*:: . :   .:  . .  *  ::.      *:  :::..:*
                                  D or N
MA_16619p0010       ---YSTTLNDGGQLSNPMTEIEVSSLYYQKRERVCS-S-----DQSKTNNGDSLYECL--      232
AT2G02450           SNHSDNNLNNNN----NINNLEKLSTEYSGDGSTTTTTTNSNSDVTIALANQNIYRPMPY      293
                        ...**:..     :.::*  *  *.    . : :     * : :  .:.:*. :

MA_16619p0010       ---E-PIMLSEETRVLKNPKACKTALLELPNMSL-------------------------N      263
AT2G02450           DTSNNTLIVSTRNHQDDDETAIVDDLQRLVNYQISDGGNINHQYFQIAQQFHHTQQQNAN      353
                       :  :::* ..:  .: .*    * .* * .:                         *

MA_16619p0010       CPLSQPIATPPALLSTPPPFQPSL----I-------------------------------      288
AT2G02450           ANALQLVAAATTATTLMPQTQAALAMNMIPAGTIPNNALWDMWNPIVPDGNRDHYTNIPF      413
                    .   * :*:  :  :  *  * :*    *

MA_16619p0010       -      288
AT2G02450           K      414
```

Fig.S17 Domains of SCREAM 2 (SCRM2)

Missense polymorphism sites are highlighted in green in the Norway spruce gene sequences

Reference and alternative amino acids are displayed in dotted box

Well characterized domains of the genes are highlighted in other colors in the *Arabidopsis thaliana* gene sequences

MA_10435231g0010 [327 residues] Norway spruce (http://congenie.org/)

AT1G12860 [450 residues] *Arabidopsis thaliana* (https://www.arabidopsis.org)

```
CLUSTAL O(1.2.4) multiple sequence alignment


MA_10435231p0010      ------------------------------------------------------------   0
AT1G12860             MNSDGVWLDGSGESPEVNNGEAASWVRNPDEDWFNNPPPPQHTNQNDFRFNGGFPLNPSE   60


MA_10435231p0010      -------------------------------------------MEMEMAWTGLLDKNFSS   17
AT1G12860             NLLLLLQQSIDSSSSSSPLLHPFTLDAASQQQQQQQQQQEQSFLATKACIVSLLNVPTIN   120
                                                                 :  :  .  ..**:     .

MA_10435231p0010      LRAMLDGGGEDGFYATMDANGWTLEALLGSSLSSPGGSVLSNVGGSI----NA--SHHLS   71
AT1G12860             NNTFDDFGFDSGFLGQQ---------FHGN-HQSPNSMNFTGLNHSVPDFLPAPENSSGS   170
                       .:: *  * :.** .           : *.   .**..  ::.:. *:     *   .   *

MA_10435231p0010      CSMSTS----------VHTLEGPGGSDQLGLLHRRDVIESGIHKNGGNQKPNG----GVG   117
AT1G12860             CGLSPLFSNRAKVLKPLQVMASS-GS-QPTLFQKRAAMRQSSSSKMCNSESSSEMRKSSY   228
                      *.:*             ::.: .   ** *   *:::* .:...  .:   *.: ..     .

MA_10435231p0010      ERGA----DSVVAV-DSNNNKYVNAANAGRGKKNGLPAKNLMAERRRRKKLNDRLLMLRS   172
AT1G12860             EREIDDTSTGIIDISGLNYESDDHNTNNNKGKKKGMPAKNLMAERRRRKKLNDRLYMLRS   288
                      **        .:: :  .  *  :.   : :*  .:***.*:****************** ****

                                                            T or M
MA_10435231p0010      VVPKVSKMDRSSILGDAAEYLQELLQRIGDLHTELTSGSCNSK---PLADTLADFPCRLN   229
AT1G12860             VVPKISKMDRASILGDAIDYLKELLQRINDLHTELESTPPSSSSLHPLTPTPQTLSYRVK   348
                      ****:*****:****** :**:******.****** *    .*.    **: *    :   *::
                         263-312: BHLH domain

                                S or N
MA_10435231p0010      GELQASF---SSTVEPAKVEVRSREGKSLHINMFCSEKPGLLLSTIRALDELGLDVKQAV   286
AT1G12860             EELCPSSSLPSPKGQQPRVEVRLREGKAVNIHMFCGRRPGLLLSTMRALDNLGLDVQQAV   408
                       **   *     *  .  :   :**** ****:::*:***  ..:*******:****:*****:***
                         378-450: ACT domain: involved in the binding of small ligands

MA_10435231p0010      ISCLNGFALDVLQEKQSM-GGDVTAEEIKTLLLHTAGYQAGL    327
AT1G12860             ISCFNGFALDVFRAEQCQEDHDVLPEQIKAVLLDTAGYAGLV    450
                      ***:*******::  :*.   .  **   *:**::**.****  .  :
```

Fig.S18 Domains of NAC DOMAIN CONTAINING PROTEIN 36 (NAC036)
Missense polymorphism sites are highlighted in green in the Norway spruce gene sequences
Reference and alternative amino acids are displayed in dotted box
Well characterized domains of the genes are highlighted in other colors in the *Arabidopsis thaliana* gene sequences
MA_101849g0010 [306 residues] Norway spruce (http://congenie.org/)
AT2G17040 [276 residues] *Arabidopsis thaliana* (https://www.arabidopsis.org)

```
CLUSTAL O(1.2.4) multiple sequence alignment


MA_101849p0010      -------------------------MAMPDKICN------DGVVQGELMDLPGFRFYPTE     29
AT2G17040           MNSDGVWLDGSGESPEVNNGEAASWVRNPDEDWFNNPPPPQHTNQNDFRFNGGFPLNPSE     60
                                             :  **:         : . *.::    ** : *:*

MA_101849p0010      EELLSFYMKKKIQAGHS-------LN-----------------F---------DKIIPT     55
AT2G17040           NLLLLL--QQSIDSSSSSSPLLHPFTLDAASQQQQQQQQQQEQSFLATKACIVSLLNVPT     118
                    : ** :  ::.*::. *       :.                  *            :**
                         6-152: NAC domain: DNA-binding and dimerization domain

MA_101849p0010      MDLYQYDPWELPGFAHDAGEQQWFFFVPRYHNKRARPNRLAVSGYWKATGSDRAVRNELL     115
AT2G17040           INNNTFDD-----FGFDSG-----FLGQQFHGNHQSPNSMNFTGLNHSVPDFLPAPENSS     168
                    ::   :*      *..*:*     *:  ::*.::  ** : .:*  ::. .   . ::

MA_101849p0010      QCIGLKKILV-------FYKGKSPYGQKTDWIMNEYRMPEFSSSAPKKKMDMVLCRIYRK     168
AT2G17040           GSCGLSPLFSNRAKVLKPLQVMASSGSQPTLFQKRAAMRQ--------SSSSKMCNSESS     220
                     . **. ::          :  :  *.:   : :.  * :        . .  :*.   .

MA_101849p0010      TATRKSLEQRAKTNGVDKEEPVVSSDVYEDKSHSLT----------------------V     205
AT2G17040           SEMRKSSYEREIDDT-STGIIDISGLNYESDDHNTNNNKGKKKGMPAKNLMAERRRRKKL     279
                    :  ***  :*   :  ..    :*.  **...*. .                       :

                    D or E
MA_101849p0010      DVRCLSN----PM-------------------------------------------IEAK     218
AT2G17040           NDRLYMLRSVVPKISKMDRASILGDAIDYLKELLQRINDLHTELESTPPSSSSLHPLTPT     339
                    : *        *                                            :  .

                                      T or M
MA_101849p0010      VSSVQYLDDEGVCSSRSKTKDESS---------LFDCLEPIMFCEDEETTVLKTSK----     265
AT2G17040           PQTLSYRVKEELCPSSSLPSPKGQQPRVEVRLREGKAVNIHMFCGRRPGLLLSTMRALDN     399
                     .::.*  .* :* * *  . :..           ..::  ***  .   :*.* :

MA_101849p0010      ----------------------PKACKPPQLELPKLPMDFLFGQQITTPYALLATPSPFQ     303
AT2G17040           LGLDVQQAVISCFNGFALDVFRAEQCQEDHDVLPEQIKAVLLD---TAGYAGLV------     450
                                           : *:  :  **:    .*:.   *: ** *.

MA_101849p0010      IPL   306
AT2G17040           ---   450
```

Fig.S19 Domains of EXPANSIN B3 (EXPB3)

Missense polymorphism sites are highlighted in green in the Norway spruce gene sequences

Reference and alternative amino acids are displayed in dotted box

Well characterized domains of the genes are highlighted in other colors in the *Arabidopsis thaliana* gene sequences

MA_7354451g0010 [71 residues] Norway spruce (http://congenie.org/)

AT4G28250 [264 residues] *Arabidopsis thaliana* (https://www.arabidopsis.org)

```
CLUSTAL O(1.2.4) multiple sequence alignment
                            K or R  R or S                      C or R

MA_7354451p0010      MRKTVRLQWPLVPNFCLDVTAFCFVLLLLFSCKEPALGLEHTELNRDDLEWRPATATWYG    60
AT4G28250            -----------MQLFPVMLATLCIVLQLLIGSS----ALATTNRHVSNSHWLPAVATWYG    45
                                :  *  : ::::*:** **:...    .*   *: :  .: .* **.*****

MA_7354451p0010      SPDGDGSDGIL-------------------------------------------------    71
AT4G28250            SPNGDGSDGGACGYGTLVDVKPLHARVGAVNPILFKNGEGCGACYKVRCLDKSICSRRAV    105
                     **:******

MA_7354451p0010      ------------------------------------------------------------    71
AT4G28250            TVIITDECPGCSKTSTHFDLSGAVFGRLAIAGESGPLRNRGLIPVIYRRTACKYRGKNIA    165

                         54-162: Domain: Expansin-like EG45

MA_7354451p0010      ------------------------------------------------------------    71
AT4G28250            FHVNEGSTDFWLSLLVEFEDGEGDIGSMHIRQAGAREWLEMKHVWGANWCIIGGPLKGPF    225

                         175-256: Domain: Expansin-like CBD

MA_7354451p0010      ---------------------------------------         71
AT4G28250            SIKLTTLSAGKTLSATDVVPRNWAPKATYSSRLNFSPVL         264
```

Fig.S20 Domains of FCS LIKE ZINC FINGER 6 (FLZ6)

Missense polymorphism sites are highlighted in green in the Norway spruce gene sequences

Reference and alternative amino acids are displayed in dotted box

Well characterized domains of the genes are highlighted in other colors in the *Arabidopsis thaliana* gene sequences

MA_14341g0010 [180 residues] Norway spruce (http://congenie.org/)

AT1G78020 [162 residues] *Arabidopsis thaliana* (https://www.arabidopsis.org)

```
CLUSTAL O(1.2.4) multiple sequence alignment


MA_14341p0010      MFEDHLRFDNLAAVPLDLEVELAFPEILSEEMMRYPGKRPCRPRPAAAPIRRTSSTAML      60
AT1G78020          ---------------------------------MLLGKRQ-------RPPINRTTSLSEI      20
                                                   ***          **.**:* : :

                                                                   L or V
MA_14341p0010      DSALNV-----------------------------DHDDRYKQSPEDGGASRKAAALTSH      91
AT1G78020          KFDLNLPSESEPSNQQKPTVASPYGSNGQAVTAAVDQNRGFLDQR-LLSMVTPRGNLRRH      79
                   .  **:                             *::  : :.    .     . *  *

MA_14341p0010      VHVFPEVAYFLQACFLCKRRLGPDTDIYMYRGDAAFCSAECRHEQIVIDERKEKCSAEVR      151
AT1G78020          SGDFSDAGHFLRSCALCERLLVPGRDIYMYRGDKAFCSSECRQEQMAQDERKEKGKSAAP      139
                      * :..:**::* **:* * *. ******** ****:***:**:. ****** .: .
```

88-132: FLZ-type zinc finger: likely to be involved in protein-protein interaction.

```
MA_14341p0010      KMKE-SSAPANNRQSSSTNQSVRAGTVAAA- 180
AT1G78020          AKEPAVTAPARAKPG--------KGRAAAAV 162
                     :   :***. : .         * .***
```

Fig.S21 Domains of VASCULAR-RELATED RLK 1 (VRLK1)
Missense polymorphism sites are highlighted in green in the Norway spruce gene sequences
Reference and alternative amino acids are displayed in dotted box
Well characterized domains of the genes are highlighted in other colors in the *Arabidopsis thaliana* gene sequences
MA_587505g0010 [635 residues] Norway spruce (http://congenie.org/)
AT1G79620 [971 residues] *Arabidopsis thaliana* (https://www.arabidopsis.org)

```
CLUSTAL O(1.2.4) multiple sequence alignment


MA_587505p0010      ------------------------------------------------------------      0
AT1G79620           MVGSNTVTATSRLLLICFAYSFTVFSMISSVTDPRDAAALRSLMDQWDNTPPSWGGSDDP      60


MA_587505p0010      ------------------------------------------------------------      0
AT1G79620           CGTPWEGVSCNNSRITALGLSTMGLKGRLSGDIGELAELRSLDLSFNRGLTGSLTSRLGD      120


MA_587505p0010      ------------------------------------------------------------      0
AT1G79620           LQKLNILILAGCGFTGTIPNELGYLKDLSFLALNSNNFTGKIPASLGNLTKVYWLDLADN      180


MA_587505p0010      ---------------------------------------------------MGGNGFHLS      9
AT1G79620           QLTGPIPISSGSSPGLDLLLKAKHFHFNKNQLSGTIPPKLFSSEMILIHVLFDGNRFTGS      240
                                                                       :.** *  *

MA_587505p0010      LILPIAVIITIAG--VMATGMKGKDESKCPYDLTVASRMIP-------------------      48
AT1G79620           IPSTLGLIQTLEVLRLDRNTLTG----KVPENLSNLTNIIELNLAHNKLVGSLPDLSDMK      296
                    :   :.:* *:    :  . :.*    * * :*:  :.:*

MA_587505p0010      SSCYANNTDQTIE-KNTCCWS-VFASYVFSISLYANRTGTVLLDANGS------RHCTN-      99
AT1G79620           SMNYVDLSNNSFDPSESPLWFSTLPSLTTLVMEYGSLQGPLPNKLFGFPQLQQVRLKKNA      356
                    *  *.: :::::: .::  *  .: * .  :  *..  * :  .  *       *  .*

MA_587505p0010      -----------------------EFVRFVRSRGLVQPGFFSNNSSCAINPLIFTAHAGPC      136
AT1G79620           FNGTLSLGDTVGPELQLVDLQDNDISSVTLSSGYTNTLILEGNPVCTTA-------LSNT      409
                                           ::  .. * * .:  ::..*  *:          .

MA_587505p0010      RYATLGDISNVIDVGNASIACDGPSLN-----ESKACERC--QEK-VISKALELLNLTNS      188
AT1G79620           NYCQIQQQQVKRIYSTSLANCGGKSCPLDQKVSPQSCECAYPYEGTLYFRGPMFRDLSNV      469
                    .*. : : .     ..:   *.* *       . ::** .   *  :  :.  : :*:*

MA_587505p0010      KEFVPCGMAITMGIWGRDP---SEE-------RFGGYVGCVLQVLGNIG-----------      227
AT1G79620           NTY----HSLEMSLWVKLGLTPGSVSLQNPFFNNDDYLQIQLALFPPMGKYFNRTEVQRI      525
                    : :     :: *.:* :     ..        . ..*:   * ::  :*
                                                          A or V
MA_587505p0010      GLGTSSLIPSPPPP--PLPAS----HEPSGGNKK-GRGRVRIAVAAGIAATSVVLLVFAA      280
AT1G79620           GFDLSNQTYKPPPLFGPYYFIASPYTFPADGNGHSLSSRMVTGIITGCSALVLCLVALGI      585
                    *:. *.   .***   *          *:.** :   .*:  .: :* :*  : *:.:.
                    V or L
MA_587505p0010      LIVLKSKKTNGPSEESTASSSLQVLTRSLMGIDKRTETVAREPLPTEGLYLFTKRELQRA      340
AT1G79620           YAMWQKRRAEQA----------IGLSRPFVSWASSGKDS-GGAPQLKGARWFSYEELKKI      634
                      : :.::::              *:* ::.  .  :         :*   *: .**::

MA_587505p0010      TEDFSEARVLGTGGSGKVYLGKLPSGKQVAIKRIVQKRK--VDEFYKEVELLSRLRHPNL      398
AT1G79620           TNNFSVSSELGYGGYGKVYKGMLQDGHMVAIKRAQQGSTQGGLEFKTEIELLSRVHHKNL      694
                    *::** :  ** ** **** * * .*: *****  *  .    ** .*:*****::* **
                          638-912: Domain: Protein kinase

MA_587505p0010      ASLLGYCQHRREHILVYEYMPGGDLGCLLFRREDPVLPWDRRLRIALDCAEGLAHLHLFP      458
AT1G79620           VGLVGFCFEQGEQILVYEYMSNGSLKDSLTGRSGITLDWKRRLRVALGSARGLAYLHELA      754
                    ..*:*:* .: *:******* .*.*   *  *.. .* *.****:**..*.***:** :
```

```
MA_587505p0010      EGAIVHRDIKPTKLSDFGISKLIAPDISHASTEIKGTTGFLDPEYFFVGKLNILLNESGQ     518
AT1G79620           DPPIIHRDVKST---------------------------------------NILLDENLT     775
                    :  *:***:* *                                       ****:*.

MA_587505p0010      AKLSDFGVSKLIAP-DFSHASTEIKGTTGYVDPEYFTVGRLTDASDVYSFGVVLLQLISG     577
AT1G79620           AKVADFGLSKLVSDCTKGHVSTQVKGTLGYLDPEYYTTQKLTEKSDVYSFGVVMMELITA     835
                    **::***:***::    .*.**::*** **:****:*. :**: *********:::**:.

MA_587505p0010      QKAVISTPSGGADSVVYMAHVFMSGEDPDVRNLVDPRIADAMDARDLGSIK---MVLDIA     634
AT1G79620           KQPIEKG-----KYIVREIKLVMNKSDDDFYGLRDKMDR---SLRDVGTLPELGRYMELA     887
                    :: : .      . :*   ::.*. .* *. .* *       . **:*::      :::*

MA_587505p0010      Y-----------------------------------------------------------     635
AT1G79620           LKCVDETADERPTMSEVVKEIEIIIQNSGASSSSSASASSSATDFGEKLLYGGTLKKKEA     947


MA_587505p0010      ------------------------      635
AT1G79620           RDGDGGGAFDYSGGYSVPTKIEPK      971
```

Fig.S22 Domains of MYB DOMAIN PROTEIN 3 (MYB3)
Missense polymorphism sites are highlighted in green in the Norway spruce gene sequences
Reference and alternative amino acids are displayed in dotted box
Well characterized domains of the genes are highlighted in other colors in the *Arabidopsis thaliana* gene sequences
MA_7115g0010 [293 residues] Norway spruce (http://congenie.org/)
AT1G22640 [257 residues] *Arabidopsis thaliana* (https://www.arabidopsis.org)

```
CLUSTAL O(1.2.4) multiple sequence alignment


MA_7115p0010      MG-RSCCSKEGLNRGAWSRKEDMILSEYIRIHGDGGWTNLPQRAGLKRCGKSCRLRWTNY      59
AT1G22640         MGRSPCCEKAHMNKGAWTKEEDQLLVDYIRKHGEGCWRSLPRAAGLQRCGKSCRLRWMNY      60
                  **   **.*  :*:***:::** :* :*** **:* * .**: ***:********** **
                          9-61: Myb-type1 HTH DNA-binding domain

MA_7115p0010      LRPDIKLGNISPDEDELIIRMHRLLGNRWSLIAGRLPGRTDNEIKNYWNTRLSKKLLSID      119
AT1G22640         LRPDLKRGNFTEEEDELIIKLHSLLGNKWSLIAGRLPGRTDNEIKNYWNTHIKRKLLSRG      120
                  ****:* **:: :******::* ****:**********************:..:****  .
                          62-116: Myb-type2 HTH DNA-binding domain

MA_7115p0010      DSQSKSTARNLETSSKSPPPPPNHVFKTTPIKITTAVKFSETVGPKRCNG-YG-RSNCSS      177
AT1G22640         IDPNS--HRLINESVVSPSSLQNDVVETIHL------DFSGPVKPEPVREEIGMVNNCES      172
                   . ..   * ::  *  **    *.*.:*  :      .**  * *:  .   *  .**.*

MA_7115p0010      AEAIKLCNIKENTNSIPHDDSEHIVFDMT----------DVNLLETEAGNCTSAIWSLEE      227
AT1G22640         SGT---------TSEKDYGNEEDWVLNLELSVGPSYRYESTRKVSVVDSAESTRRWGSE-      222
                  : :         *..  :.:.*. *:::           ... :..  .  ::  *. *

MA_7115p0010      ERSPHSYYFAVDTATLDESLSELNVLSSPDCNLLYSASGSSTDFELEEFYREAATLATGI      287
AT1G22640         -------LFGAHESDAVCLCCRIGLFRNESCRNC----------RVSDVRTH--------      257
                          *... :      ..:.:: . .*.            .:.:.  .

MA_7115p0010      NDSVFT  293
AT1G22640         ------  257
```

Fig.S23 Domains of RESISTANT TO P. SYRINGAE 2 (RPS2)
Missense polymorphism sites are highlighted in green in the Norway spruce gene sequences
Reference and alternative amino acids are displayed in dotted box
Well characterized domains of the genes are highlighted in other colors in the *Arabidopsis thaliana* gene sequences
MA_475302g0010 [303 residues] Norway spruce (http://congenie.org/)
AT4G26090 [910 residues] *Arabidopsis thaliana* (https://www.arabidopsis.org)

```
CLUSTAL O(1.2.4) multiple sequence alignment


MA_475302p0010      ------------------------------------------------------------     0
AT4G26090           MDFISSLIVGCAQVLCESMNMAERRGHKTDLRQAITDLETAIGDLKAIRDDLTLRIQQDG     60

                      29-58: Coiled coil domain - essential for the resistance to AvrRpt2
MA_475302p0010      ------------------------------------------------------------     0
AT4G26090           LEGRSCSNRAREWLSAVQVTETKTALLLVRFRRREQRTRMRRRYLSCFGCADYKLCKKVS     120


MA_475302p0010      ------------------------------------------------------------     0
AT4G26090           AILKSIGELRERSEAIKTDGGSIQVTCREIPIKSVVGNTTMMEQVLEFLSEEEERGIIGV     180

                         135-440: NB-ARC domain - nucleotide-binding domain
MA_475302p0010      ------------------------------------------------------------     0
AT4G26090           YGPGGVGKTTLMQSINNELITKGHQYDVLIWVQMSREFGECTIQQAVGARLGLSWDEKET     240


MA_475302p0010      ------------------------------------------------------------     0
AT4G26090           GENRALKIYRALRQKRFLLLLDDVWEEIDLEKTGVPRPDRENKCKVMFTTRSIALCNNMG     300


MA_475302p0010      ------------------------------------------------------------     0
AT4G26090           AEYKLRVEFLEKKHAWELFCSKVWRKDLLESSSIRRLAEIIVSKCGGLPLALITLGGAMA     360


MA_475302p0010      ------------------------------------------------------------     0
AT4G26090           HRETEEEWIHASEVLTRFPAEMKGMNYVFALLKFSYDNLESDLLRSCFLYCALFPEEHSI     420


MA_475302p0010      ------------------------------------------------------------     0
AT4G26090           EIEQLVEYWVGEGFLTSSHGVNTIYKGYFLIGDLKAACLLETGDEKTQVKMHNVVRSFAL     480


MA_475302p0010      ------------------------------------------------------------     0
AT4G26090           WMASEQGTYKELILVEPSMGHTEAPKAENWRQALVISLLDNRIQTLPEKLICPKLTTLML     540

                  512-533: Leucine-rich repeat 1 (LRR) - probably act as specificity determinant
of pathogen recognition
MA_475302p0010      ---MKLKEIP-----------ALDIGNQAAHGGFPMSNAKTMLQLQILKIQGCRVLKTFR     46
AT4G26090           QQNSSLKKIPTGFFMHMPVLRVLDLSFTS-ITEIPLSI-KYLVELYHLSMSGTKIS--VL     596
                        .**:**           .**:.  :    :*:*  * :::*  *.:.* ::   .
                         534-556: LRR 2            559-580: LRR 3
MA_475302p0010      VEELPNLRKLG---ISHCAELKELSMES------------------------------GG     73
AT4G26090           PQELGNLRKLKHLDLQRTQFLQTIPRDAICWLSKLEVLNLYYSYAGWELQSFGEDEAEEL     656
                     :** *****    :.:   *: :  ::
                      582-604: LRR 4    605-627: LRR 5
MA_475302p0010      GLLRLERL--------DLWSLQRLESIVWNAKTMLQIQFFGISNCHVLKTFGVEELP---     122
AT4G26090           GFADLEYLENLTTLGITVLSLETLKTLFEFGALHKHIQHLHVEECNELLYFNLPSLTNHG     716
                    *:  ** *         : **: *:::.  .    :**.: :.:*: *  *.: .*


MA_475302p0010      -SLKEINISNCVELKESFM---ESGGSLPMLEILELYSLEIRSIVWNEKTM----LQLQS     174
AT4G26090           RNLRRLSIKSCHDLEYLVTPADFENDWLPSLEVLTLHSLHNLTRVWGNSVSQDCLRNIRC     776
                     .*:.:.*..* :*:  .     ... ** **:* *:**.  : **.:..      :::.


MA_475302p0010      FYISYCRVLKTF-RVEELPNLKEIEIKQCPELQVQIGS-----------VPMLKKLTLEG     222
```

```
AT4G26090           INISHCNKLKNVSWVQKLPKLEVIELFDCREIEELISEHESPSVEDPTLFPSLKTLRTRD     836
                    : **:*. **..  *::**:*: **: :* *::  *..           .* **.*  ..

MA_475302p0010      LKKSESIARASSVWNKETMPKLEYINITGCPLLRRLPMEMDK-LPNLKKIVGEVGWWEGI     281
AT4G26090           LPELNSI-----LPSRFSFQKVETLVITNCPRVKKLPFQERRTQMNLPTVYCEEKWWKAL     891
                    * : :**     : .: :: *:* : **.** :::**::  :   ** .:  *  **:.:

MA_475302p0010      NWEIDNVKIKLSELFRDEDEDD 303
AT4G26090           EKDQPNEELCYLPRFVPN---- 909
                    : :  * ::     *  :
```
